# Automated Active Space Selection with CASCI Dipole Moments

**DOI:** 10.1021/acs.jctc.6c00473

**Published:** 2026-07-24

**Authors:** Benjamin W. Kaufold, Sijia S. Dong

**Affiliations:** † Department of Chemistry and Chemical Biology, 1848 Northeastern University, Boston, Massachussetts 02115, United States; ‡ Department of Chemical Engineering, Northeastern University, Boston, Massachussetts 02115, United States; § Department of Physics, Northeastern University, Boston, Massachussetts 02115, United States

## Abstract

Multireference calculations are well-suited for describing properties of highly correlated systems. In order to realize the full potential of multireference methods, it is necessary to judiciously choose the active space. It is possible to do this manually, but an automatic procedure for selecting the active space can benefit high-throughput applications and is not susceptible to human error. We previously developed two protocols, GDM-AS and EDM-AS, where the dipole moment calculated at the complete active space self-consistent field (CASSCF) level of theory is used to select the active space, and demonstrated the effectiveness of these protocols when the chosen active spaces are used to find low-lying excitation energies for molecules with nonzero dipole moments. In this work, we demonstrate that using complete active space configuration interaction (CASCI) dipole moments, with the orbitals from the second-order Møller–Plesset perturbation theory, instead of CASSCF dipole moments in our dipole moment active space selection (DM-AS) protocols can significantly improve the efficiency of active space selection compared to our previous methods while maintaining reasonable complete active space second-order perturbation theory (CASPT2) and complete active space pair-density functional theory (CAS-PDFT) vertical excitation energy. We also present new protocols, CASCI-D^2^DM-AS and its directional version, designed for charge transfer states. We discuss the performance of our new protocols in their ability to describe excitation energy of not only small organic molecules tested in our previous work, but also larger conjugated polar molecules, nonpolar molecules, and bimolecular complexes, to describe the ground-state spin state of transition metal oxides, and to describe bond dissociation curves of small molecules.

## Graphical abstract

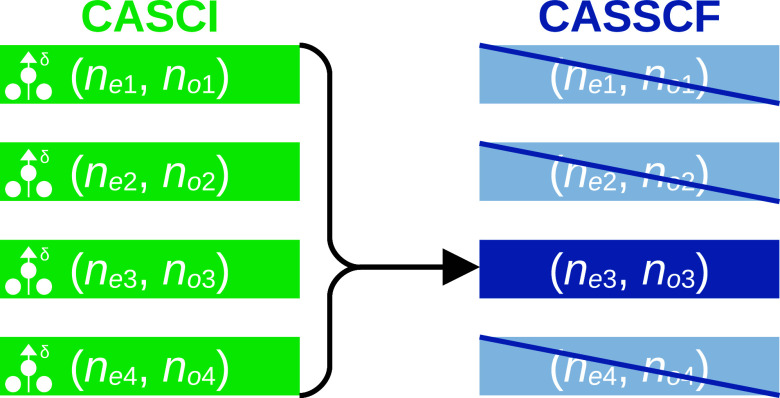

## Introduction

Multireference methods are essential for accurately describing chemical systems with strong correlation, including diradicals and transition metal complexes. Many multireference methods, such as those based on the complete active space self-consistent field (CASSCF)^1–3^ method, rely on the selection of an active space, which includes the electrons and orbitals that are more correlated. The active space dependence of multireference methods is both their greatest strength and their greatest weakness. When the active space is chosen well, electronic properties can be describes with a good balance of high accuracy and efficiency. When it is chosen poorly, results can be anything from inaccurate to physically meaningless.

It is possible to construct the active space manually, by analyzing the chemical system and deciding relevant orbitals to include. However, doing so has several drawbacks. First, the constructed active space is subject to human error. In addition, manual active space construction is a tedious process, and is not suitable for high-throughput applications where many chemical systems must be studied. Automated active space selection methods are preferable for applications where the user wishes to avoid these drawbacks. Here, the active space is built from information directly calculated from the chemical system itself, bypassing any human intervention. Numerous such schemes have been proposed.^4–16^

In a previous work,^17^ we devised two procedures which involved comparing the dipole moment calculated at the CASSCF level to a reference such as that from an experimental measurement or a calculation with density functional theory, provided that the system of interest was not highly symmetrical with zero dipole moment. We built these procedures on the premise that the accuracy of the dipole moment indicates how well the active space represents the electron density, and as such, its suitability for other calculations. We found that the active spaces selected with dipole moments performed well for obtaining low-lying excitation energies, potential energy curves and ground-state spin configurations.

The efficiency of these protocols is determined by the largest active space (in terms of the number of determinants) that the user is willing to scan. CASSCF must be run at all active spaces eligible for selection, since CASSCF dipole moments are compared at the active space selection step. Assuming the user has access to suitable computing resources, all of these CASSCF calculations may be run in parallel, so only the most computationally expensive CASSCF calculation determines the total run time. One way we have found to improve the efficiency of the protocols is to limit the maximum active space size. We demonstrated that, even if certain large active spaces are removed from consideration, the protocols still choose useful active spaces among the remaining candidates. Specifically, when we limit active spaces to be no larger than (8,14), the time to solution improves by a factor greater than 10 compared to when active spaces up to (14,14) are allowed, with a negligible increase in average excitation energy error.^17^

In this work, we present new active space selection protocols with even more improvements to the efficiency. The core of the improvement is to replace the use of CASSCF at the active space selection step with the much faster complete active space configuration interaction (CASCI)^18–20^ method. The improvement on the efficiency comes from the fact that CASCI requires only one iteration for optimizing the configuration interaction coefficients and none for optimizing orbitals, while CASSCF requires multiple iterations of optimization for the configuration interaction coefficients and the orbitals. As such, a CASCI calculation for a given input system and active space is almost always faster than CASSCF for the same system and active space, with the difference being more pronounced if more CASSCF iterations are required. By replacing the set of CASSCF calculations for candidate active spaces with CASCI, it is possible to further reduce the time-to-solution by preventing CASSCF from being run on a large active space which might not be selected.

Since orbitals are not optimized, CASCI may be less accurate than CASSCF for describing properties such as the dipole moment. However, our protocols do not require that the dipole moment is calculated with the highest accuracy possible at the active space selection phase. Due to the complex relationship between the accuracy of dipole moments and the accuracy of the excitation energy, our main goal is to identify active spaces that can generate reasonable excitation energy and to rule out the active spaces that give unphysical results. We found that using orbitals from the second-order Møller–Plesset perturbation theory (MP2)^21–26^ and incorporating new dipole moment evaluation schemes allow our protocols to achieve this goal. Our demonstration of the suitableness of the use of CASCI in place of CASSCF in the active space selection resonates with prior work in the literature that argues that CASCI, instead of or in addition to CASSCF, can be suitable for describing the electronic excited states in certain scenarios.^27^

In addition to demonstrating that using CASCI is sufficient for active space selection, we also demonstrate the effectiveness of using CASCI over CASSCF to reduce the run time of active space selection. As such, we present a set of protocols that make the active space selection process fast, flexible and simple to implement.

## Methods

### Molecular Data Set

To evaluate our active space selection protocols, we started from a set of 25 small organic molecules we analyzed in our previous publication^17^ concerning dipole moment-based active space selection as a starting point for assembling the data set for this work. These molecules were selected based on the following criteria, which follow our previous work:^17^

- The experimental ground-state dipole moment can be found in the NIST^28^ database.
- At least one reference excitation energy is available in the QUEST database (QUESTDB).^29–31^

- The ground state is singlet.
- The molecule is charge neutral.
- The total ground-state dipole moment must be nonzero. As with the protocols based on CASSCF, some of the protocols discussed in this work are designed to work with polar molecules. However, we have separately tested the performance of the protocols for nonpolar molecules as their excited states may have nonzero dipole moments. Some of the protocols do work with nonpolar molecules that have zero dipole moment in the ground state, which are discussed later in the article.

The main data set we use in this work is referred to as “NIST and QUEST” (NAQ) and shown in Figure 1. The following two molecules have been removed from the data set used in our previous work:

- Pyridine, as complete active space second-order perturbation theory (CASPT2)^32^ for some candidate active spaces failed to converge.
- Difluorocarbene, as we found unusually large S_1_ excitation energy errors (≈30 eV) for certain active spaces with CASPT2 using OpenMolcas, and as such we deemed this molecule an outlier. Notably, we did not find unusually high complete active space pair-density functional theory (CAS-PDFT)^33,34^ excitation energies for difluorocarbene, and we found more reasonable CASPT2 excitation energies with a different software package, ORCA,^35–37^ suggesting that the erroneous CASPT2 excitation energy errors are due to a software issue, likely numerical instability, rather than a poor choice in active space from our protocols. A comparison of the CASPT2 excitation energy from OpenMolcas and ORCA can be found in the Supporting Information Table S1.

**Figure 1:**
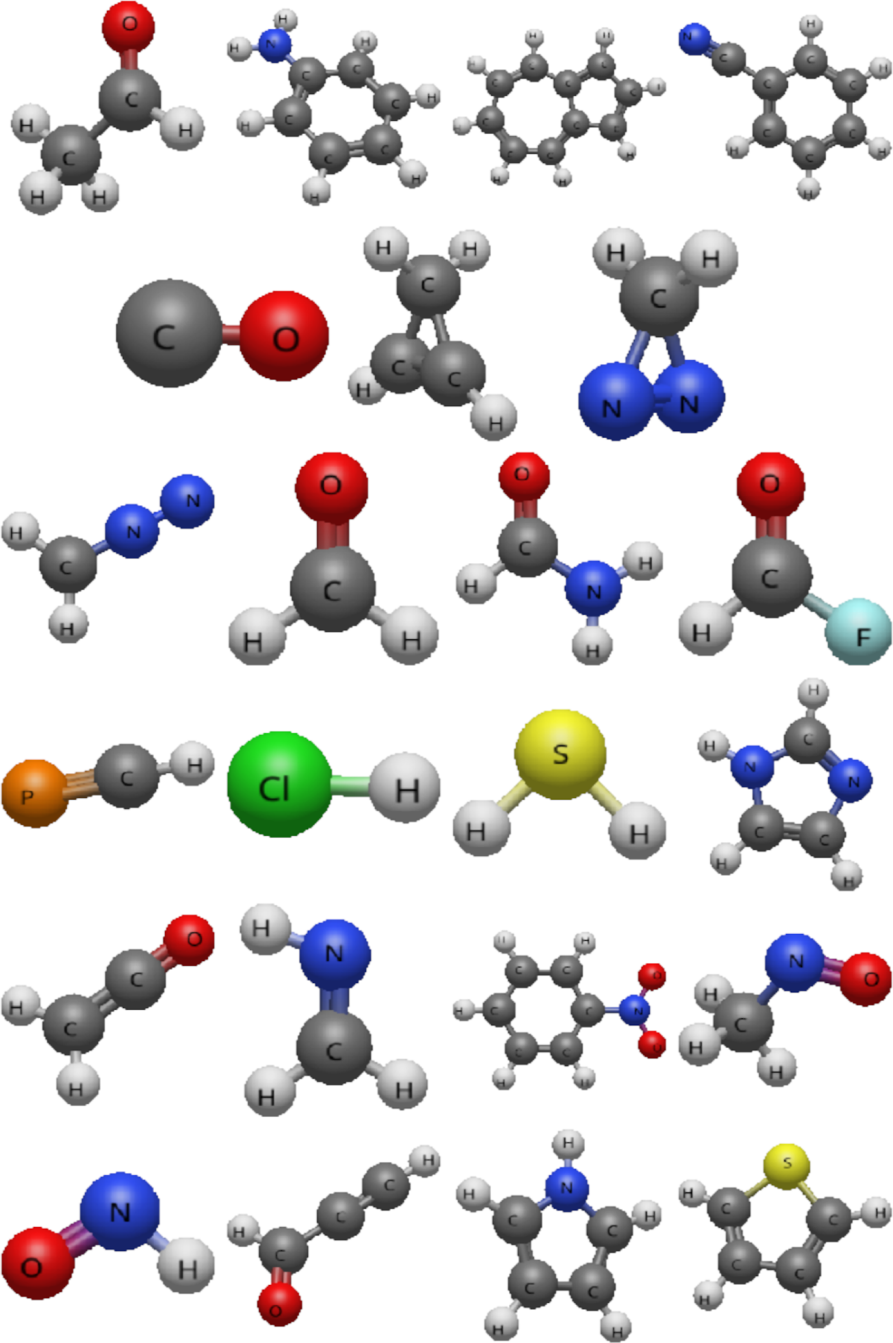
Molecules considered in this work, referred to as the “NAQ” data set, with geometries obtained from the Web site for the QUEST^29–31^ database. Coordinates of all molecules are presented in the Supporting Information section “Cartesian Coordinates”. Molecules are visualized with Avogadro2.^38^

The geometry for each molecule was obtained from the QUEST database as Cartesian coordinates. These coordinates are provided in the Supporting Information section “Cartesian Coordinates of Molecules”.

Reference excitation energies for these molecules can be found in the Supporting Information section “QUESTDB Reference Excitation Energies” and Table S2 in ref 17.

As a basic metric of the relevance of using multireference methods for electronic structure modeling of these molecules, we calculated the multireference diagnostic^39^ for each of the 23 molecules in NAQ in the ground state, averaged over all active spaces tested. We found that the majority of molecules (19) have a multireference diagnostic of at least 0.05, indicating modest^39^ multireference character; using multireference methods is likely useful for these molecules.

In order to determine the performance of the protocols for data sets beyond the initial set of small organic molecules (e.g., those in NAQ) the protocols were designed for, we have assembled the following additional data sets:

- A set of 13 polar molecules from QUEST with some degree of conjugation, most of which possess aromatic groups, in order to test the protocols’ ability to deal with systems more likely to have high multireference character. These molecules possess 8 to 21 atoms. These are represented in the Supporting Information Figure S1. The multireference diagnostic for this data set, averaged over all molecules and active spaces, is 0.11, indicating high multireference character^39^ and a strong dependence on multireference methods for proper modeling. The minimum multireference index for any molecule, averaged over all active spaces, is 0.07.
- A set of 5 molecules from QUEST that are nonpolar in the ground state, varying from 12 to 18 atoms. These are represented in the Supporting Information Figure S2.
- A set of 5 transition metal monoxides—ScO, TiO, VO, CrO and MnO—studied in the work of Wagner and Mitas.^40^
- A set of 7 bimolecular complexes studied in the work of Kozma et al.,^41^ to determine how the protocols handle systems that may exhibit intermolecular charge transfer excitations. These are represented in the Supporting Information Figure S3.

### Automated Active Space Selection Protocols

We tested three active space selection protocols in this work. Two of them, the ground-state dipole moment active space (GDM-AS) and the excited-state dipole moment active space (EDM-AS) protocols, were implemented similarly to our previous work, with modifications which are described in more detail below. The major differences are that CASCI was used instead of CASSCF at the active space selection phase and MP2 orbitals were used for CASCI calculations. A third protocol, the double differences in dipole moment active space (D^2^DM-AS) protocol, is a new protocol we developed in this work. Additionally, for each of these three protocols, we tested a variation where the direction of the dipole moment was considered in the active space selection phase, by using dipole moment vectors instead of scalar values.

To prevent confusion with the CASSCF-based active space selection protocols, we use the “CASCI-” prefix for all CASCI-based protocols from here onward, e.g., GDM-AS with CASCI dipole moments in this work is named CASCI-GDM-AS, and EDM-AS with CASCI dipole moments in this work is named CASCI-EDM-AS.

### Candidate Active Spaces

Prior to the execution of any protocol discussed in this work, the user needs to define a set of candidate active spaces. For each active space (*n*_e_, *n*_o_), the user only needs to define the number of electrons *n*_e_ and the number of orbitals *n*_o_ and does not need to specify the inclusion of specific orbitals in the active space. The protocol is then used to automatically choose one active space from the set of candidates for each molecule. The user should restrict the candidate active spaces to have a maximum size that is deemed computationally feasible given the system of interest and the user’s resources.

In this work, we chose to test the set of active spaces constrained by $\frac{n_{e}}{2}\in N$ , 6 ≤ *n*_e_ ≤ 14, $n_{o}\in N$ , $\frac{n_{e}}{2}+3\leq n_{o}\leq 14$ . This set was designated “PASS” in our previous work.^17^ In that work, we found that active spaces with *n*_e_ = 4 and those with $n_{o}=\frac{n_{e}}{2}+2$ were generally poor performers and could reasonably be dropped from consideration, and also that the protocols performed well even when large active spaces were also dropped from consideration.

Beyond this work, a user is free to define their own set of candidate active spaces. As an example, a reasonable procedure would be to determine the active space with the largest number of determinants which may provide a desired turnaround time, then add active spaces with the number of determinants at most this number to the set of candidates. The user may also add or omit active spaces from consideration if they are deemed to be (ir)­relevant based on physical principles. As an example, in a highly conjugated π system with 6 orbitals, the user might choose to exclude all active spaces with fewer than 6 orbitals. However, as the protocols are designed to minimize human intervention, it is not required or desired that users manually select the set of candidate active spaces. In any case, the protocols are designed to help select the active spaces within the set of candidate active spaces that are more likely to generate reasonable wave functions.

### CASCI-GDM-AS

With CASCI-GDM-AS, the active space is chosen according to the accuracy of the CASCI ground-state dipole moment, relative to a reference value. In this protocol, the accuracy is determined as the absolute difference between calculated and reference dipole moments. The protocol to select an active space with CASCI-GDM-AS for a given molecule is as follows:

1. Identify a set of candidate active spaces, here labeled A_*n*_. Note that they need not be the same set of active spaces tested in this paper.
2. Conduct a CASCI calculation, which includes computing the dipole moment for the ground state, S_0_, for each active space A_*n*_. Orbitals for each CASCI calculation are obtained from a prior MP2 calculation. We label the computed dipole moment for a given active space μ­(S_0_, A_*n*_).
3. Determine the reference ground-state dipole moment to be used as a point of comparison. We label the reference dipole moment μ­(S_0_, ref.). In this work, we have tested the use of dipole moments determined experimentally and computed using Kohn–Sham density functional theory (KS-DFT)^42–46^ with a variety of density functionals.
4. Take the absolute difference in the CASCI-calculated dipole moment values for each active space and the reference value. We define this as μ_diff.,*n*_ = |μ­(S_0_, A_*n*_) – μ­(S_0_, ref.)|.
5. Identify the active space that gives the smallest μ_diff.,*n*_ and choose this active space for follow-up calculations with CASSCF. If μ_diff.,*m*_ = min_*n*_(μ_diff.,*n*_), then A_*m*_ is the active space chosen by CASCI-GDM-AS. Calculations that correct for dynamic correlation can follow the CASSCF calculation with the chosen active space, such as CASPT2 or CAS-PDFT calculations in this work.

A flowchart representation of CASCI-GDM-AS can be found in Figure 2. In this example schematic, the active space A_2_ is the selected active space.

**Figure 2:**
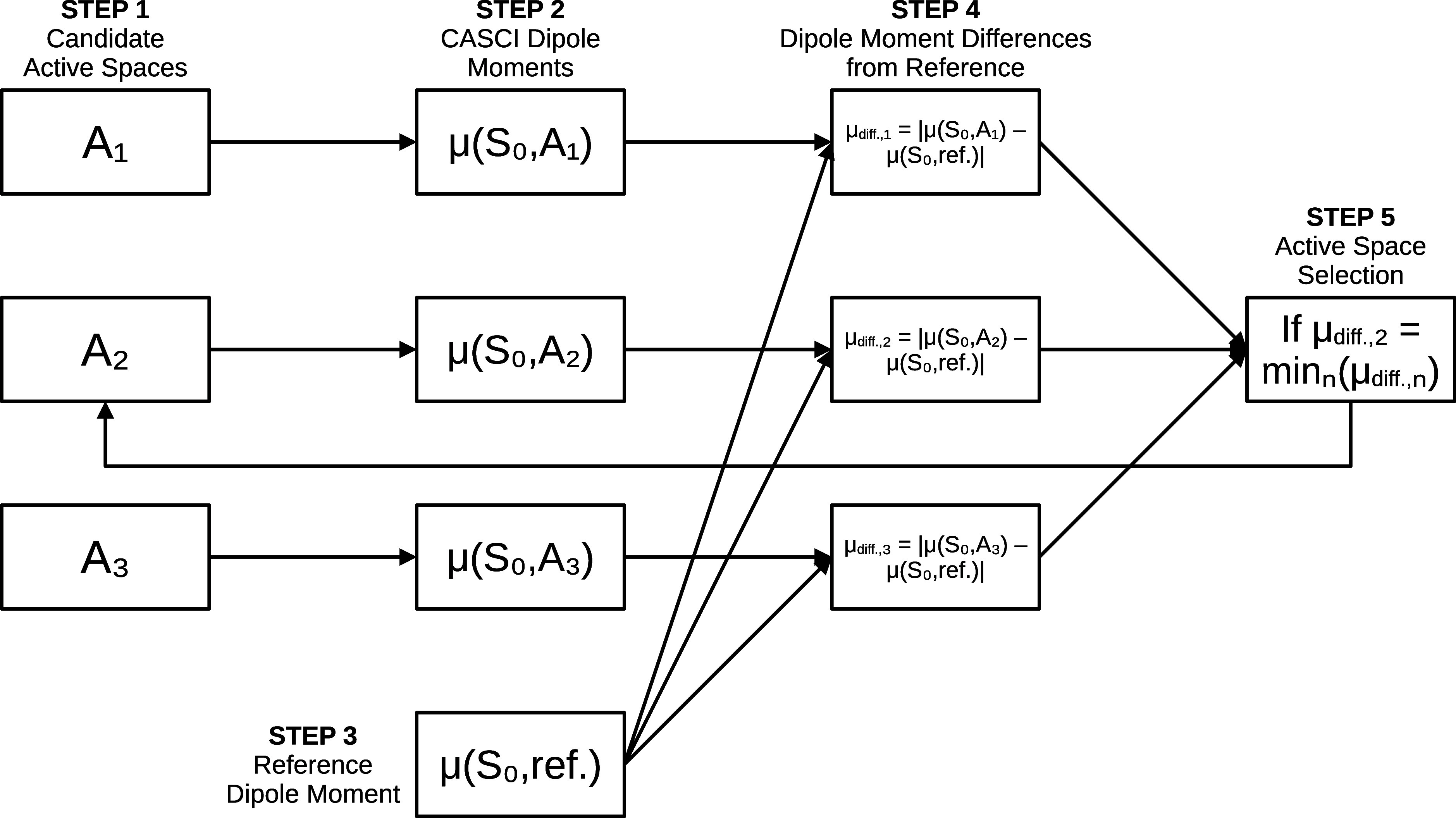
Flowchart representation of the CASCI-GDM-AS protocol.

### Directional CASCI-GDM-AS

Directional CASCI-GDM-AS (CASCI-vGDM-AS) is performed similarly to CASCI-GDM-AS, but the dipole moment difference in Step 4 is calculated using $\mu_{diff.,n}=∥\mathrm{\mu ⃗}\left(S_{0},A_{n}\right)-\mathrm{\mu ⃗}\left(S_{0},ref.\right)∥$ . This is similar to vGDM-AS in our previous publication but uses a simpler formula to define the difference of the dipole moment vectors.

### CASCI-EDM-AS

With CASCI-EDM-AS, the active space is chosen according to the accuracy of the CASCI excited-state dipole moments. All low-lying excitation energies are considered collectively. The protocol to select an active space with CASCI-EDM-AS for a given molecule is as follows:

1. Identify a set of candidate active spaces, here labeled A_*n*_. Note that they need not be the same set of active spaces tested in this paper.
2. Conduct a CASCI calculation, which includes computing the dipole moment for each excited state, S_*i*_, for each active space A_*n*_. Orbitals for each CASCI calculation are obtained from a prior MP2 calculation. We label the computed dipole moment for a given excited state and active space μ­(S_*i*_, A_*n*_).
3. Determine the reference excited-state dipole moments to be used as points of comparison. We label the reference dipole moments for the excited states μ­(S_*i*_, ref.). In this work, we have tested the use of dipole moments computed using time-dependent density functional theory (TD-DFT)^47,48^ with a variety of density functionals.
4. For each active space A_*n*_ and excited state S_*i*_, take the absolute difference in the CASCI-calculated dipole moment value and the reference value. We define this as μ_diff.,*ni*_ = |μ­(S_*i*_, A_*n*_) – μ­(S_*i*_, ref.)|.
5. For each active space A_*n*_, record the largest dipole moment difference μ_diff.,*ni*_ for any of the excited states. We define μ_diff.,*n*_ = max_*i*_(μ_diff.,*ni*_).
6. Identify the active space that gives the smallest μ_diff.,*n*_ and choose this active space for follow-up calculations with CASSCF, CASPT2 and CAS-PDFT. If μ_diff.,*m*_ = min_*n*_(μ_diff.,*n*_), then A_*m*_ is the active space chosen by CASCI-EDM-AS.

We also provide a flowchart representation of CASCI-EDM-AS in Figure 3. In this example schematic, the active space A_2_ is the selected active space.

**Figure 3:**
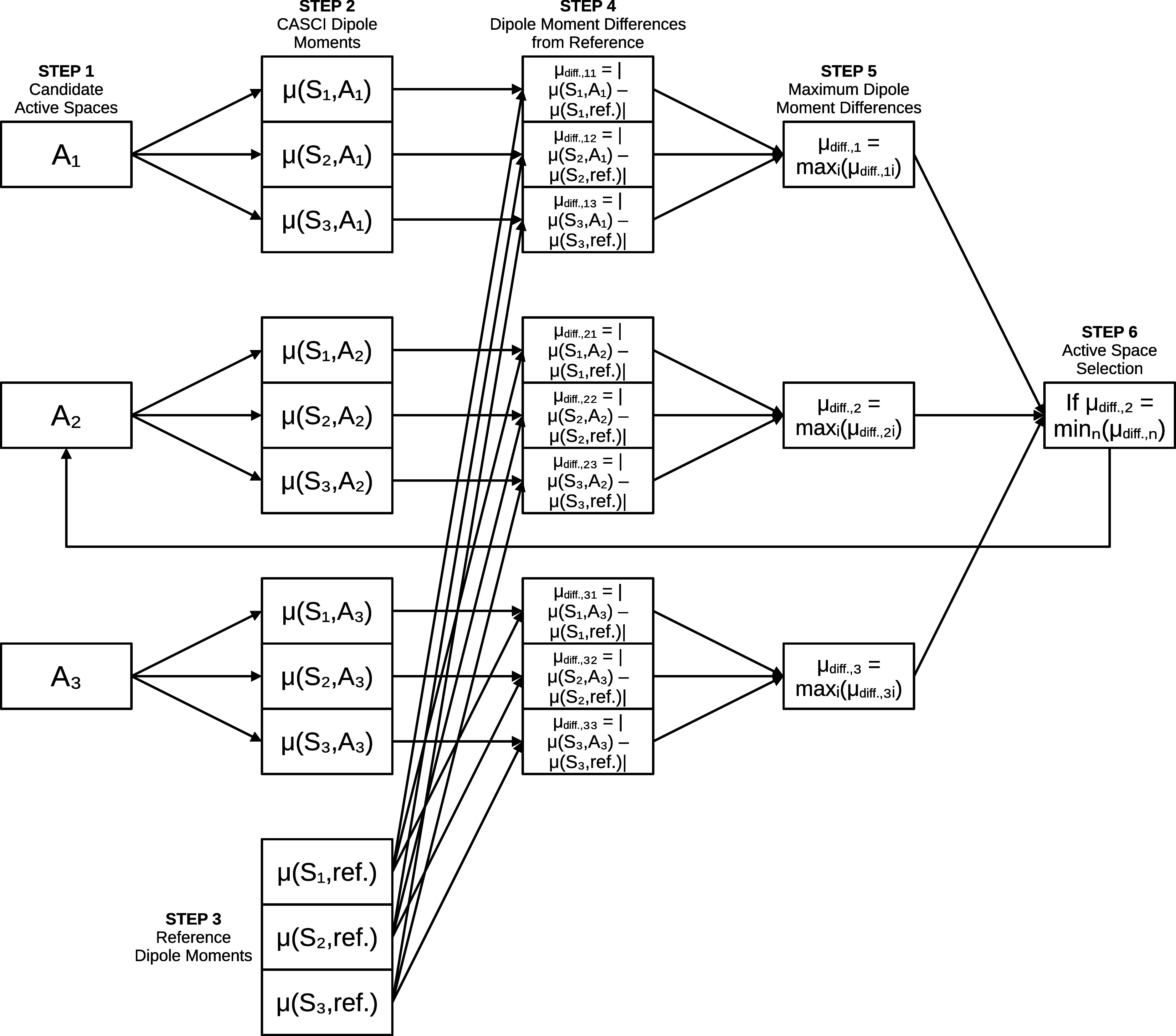
Flowchart representation of the CASCI-EDM-AS protocol.

### Directional CASCI-EDM-AS

As with CASCI-GDM-AS, we tested a directional variant of CASCI-EDM-AS (CASCI-vEDM-AS), where the dipole moment differences in Step 4 are calculated with the formula $\mu_{diff.,ni}=∥\mathrm{\mu ⃗}\left(S_{i},A_{n}\right)-\mathrm{\mu ⃗}\left(S_{i},ref.\right)∥$ .

### CASCI-D^2^DM-AS

CASCI-D^2^DM-AS is a new protocol which involves the use of excited-state dipole moments for comparison as in CASCI-EDM-AS. Instead of considering all low-lying excited states collectively as in CASCI-EDM-AS, only one excited state, whose dipole moment differs from the ground state dipole moment the most, is chosen for comparison. This protocols was designed to prioritize the reasonable prediction of charge transfer (CT) excitations, but can be applied to a variety of molecules. The relevant state is found with a simple search, by comparing the dipole moment at each state to the ground-state dipole moment. To select an active space with CASCI-D^2^DM-AS, for a given molecule, the following steps are taken:

1. Identify a set of candidate active spaces, here labeled A_*n*_. Note that they need not be the same set of active spaces tested in this paper.
2. Conduct a CASCI calculation, which includes computing the dipole moment of the ground state, S_0_, and each excited state, S_*i*_, for each active space A_*n*_. Orbitals for each CASCI calculation are obtained from a prior MP2 calculation. We use the labels μ­(S_0_, A_*n*_) and μ­(S_*i*_, A_*n*_) for the ground-state dipole moment and an excited-state dipole moment, respectively.
3. For each active space A_*n*_ and excited state S_*i*_, calculate the difference between the excited-state and the ground-state dipole moments, μ_exc.,*ni*_ = |μ­(S_*i*_, A_*n*_) – μ­(S_0_, A_*n*_)|.
4. Determine the specific CASCI excited state to use for dipole moment comparison. The excited state of interest S_*j*_ is chosen independently for each A_*n*_ such that μ_exc.,*nj*_ = max_*i*_(μ_exc.,*ni*_).
5. Conduct a TD-DFT calculation, which includes computing the dipole moment of the ground state, S_0_, and each excited state, S_*i*_. We use the same labels, μ­(S_0_, ref.) and μ­(S_*i*_, ref.) for a ground-state and excited-state dipole moment, respectively. In this work, we have tested the use of dipole moments computed using TD-DFT with a variety of density functionals.
6. For each excited state S_*i*_, calculate μ_exc.,ref.,*i*_ = |μ­(S_*i*_, ref.) – μ­(S_0_, ref.)|.
7. Determine the specific reference excited state to use for dipole moment comparison. The excited state of interest S_*k*_ is chosen such that μ_exc.,ref.,*k*_ = max_*i*_(μ_exc.,ref.,*i*_).
8. For each active space A_*n*_, take the absolute difference in the CASCI-calculated dipole moment value only the state S_*j*_ and the reference value only for the state S_*k*_. We define this as μ_diff.,*n*_ = |μ­(S_*j*_, A_*n*_) – μ­(S_*k*_, ref.)|.
9. Identify the active space that gives the smallest μ_diff.,*n*_ and choose this active space for follow-up calculations with CASSCF, CASPT2 and CAS-PDFT. If μ_diff.,*m*_ = min_*n*_(μ_diff.,*n*_), then A_*m*_ is the active space chosen by CASCI-D^2^DM-AS.

We also provide a flowchart representation of CASCI-D^2^DM-AS in Figure 4. In this schematic, the active space A_2_ is the selected active space.

**Figure 4:**
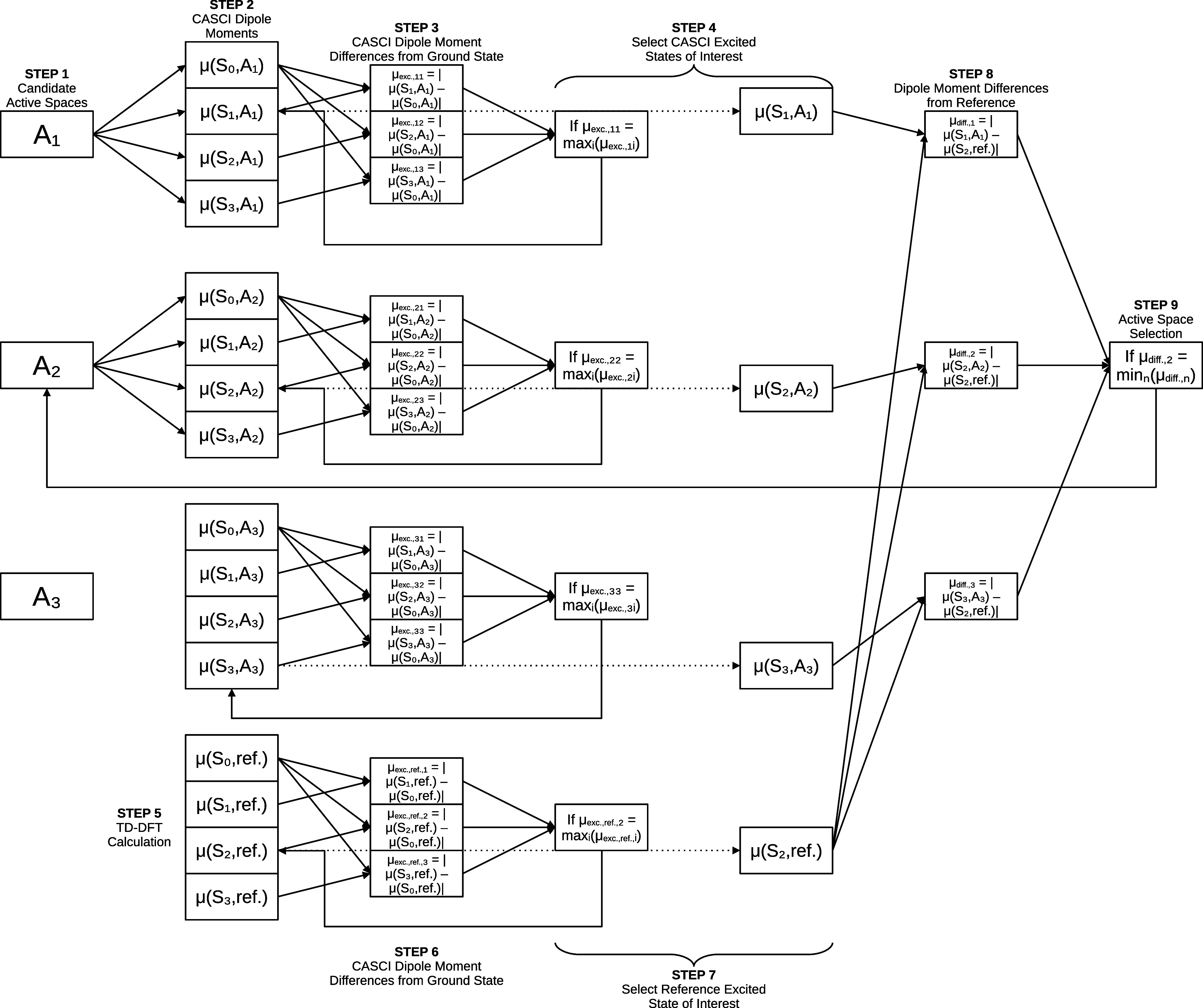
Flowchart representation of the CASCI-D^2^DM-AS protocol.

### Directional CASCI-D^2^DM-AS

In the directional variant of CASCI-D^2^DM-AS (CASCI-vD^2^DM-AS), dipole moment vector differences are taken at all steps where dipole moments are compared. For selecting the excited state of interest (Step 3), $\mu_{exc.,ni}=∥\mathrm{\mu ⃗}\left(S_{i},A_{n}\right)-\mathrm{\mu ⃗}\left(S_{0},A_{n}\right)∥$ . For selecting the reference excited state (Step 6), $\mu_{exc.,ref.,i}=∥\mathrm{\mu ⃗}\left(S_{i},ref.\right)-\mathrm{\mu ⃗}\left(S_{0},ref.\right)∥$ . For comparing the excited-state dipole moment between CASCI and a reference (Step 8), $\mu_{diff.,n}=∥\mathrm{\mu ⃗}\left(S_{j},A_{n}\right)-\mathrm{\mu ⃗}\left(S_{k},ref.\right)∥$ .

### Computational Details

OpenMolcas^49^ 22.02 was used to carry out multireference calculations. The active spaces in the set designated as PASS in our previous work were tested, and are specified by the number of active electrons *n*_e_ and the number of active orbitals *n*_o_. They adhere to the constraints $\frac{n_{e}}{2}\in N$ , 6 ≤ *n*_e_ ≤ 14, $n_{o}\in N$ , $\frac{n_{e}}{2}+3\leq n_{o}\leq 14$ . The basis set ANO-RCC-VTZP^50^ was used, and six roots (the ground state and five excited states) were calculated. In addition, relativistic treatment with the second-order Douglas–Kroll–Hess Hamiltonian^51–53^ and an ultrafine two-electron integral grid were used. OpenMP parallelization was employed for individual calculations, and four CPU cores with one thread per core were always used.

CASCI dipole moments and excitation energies were produced by first converging ground-state MP2 ^21–26^ for a given geometry, then using the resulting orbitals as input to a CASCI calculation. In the Supporting Information section “Benchmarking Starting Orbitals for CASCI”, we present data to support our use of MP2 to provide the orbitals for CASCI as opposed to Hartree–Fock or DFT methods.

CASSCF excitation energies were produced by first calculating orbitals with unrestricted^54,55^ Hartree–Fock^56^ (UHF) for a given geometry, then using the resulting orbitals as input to a CASSCF calculation. Afterward, corrections were applied with single-state complete active space second-order perturbation theory (SS-CASPT2)^32^ and complete active space pair-density functional theory (CAS-PDFT)^33,34^ to obtain excitation energies at these levels as well. When we discuss our own CASPT2 calculations, we always use SS-CASPT2.

ORCA^35–37^ 6.0.1 was used to carry out all (TD-)­DFT calculations. The functionals tested in this work include BLYP,^57–59^ B3LYP,^60^ CAM-B3LYP,^61^ PBE,^62,63^ PBE0,^64,65^ LC-ωHPBE,^66,67^ M06-L,^68,69^ M06,^69^ M06-2X,^69^ M06-HF,^70^ R^2^SCANh,^71^ R^2^SCAN0 ^71^ and R^2^SCAN50.^71^ For these calculations, the ANO-RCC-TZP^50,72^ was always used. For the sake of efficiency, the RIJCOSX approximation^73,74^ was employed, with auxiliary basis sets automatically generated according to the specification of Stoychev et al.^75^ As with OpenMolcas, relativistic treatment was accomplished with the second-order Douglas–Kroll–Hess Hamiltonian.

In both software packages, symmetry was not used. This enabled us to specify an active space by only the number of electrons and orbitals, and ensured that the Cartesian axes were consistent between software packages for comparisons of dipole moment vectors.

GNU Parallel^76^ was used for data processing.

## Results and Discussion

### Evaluation of Protocols on the NAQ Data Set

In order to establish the usefulness of the active space selection protocols, we calculate low-lying excitation energies for molecules in NAQ and determine their accuracy by comparing to reference values in QUEST. In Figures 5 through 10, we report excitation energy errors found with CAS-PDFT and CASPT2 for active spaces chosen by the protocols, averaged over all molecules in the data set. We use 0.3 eV as a soft threshold to divide satisfactory average excitation energy errors from unsatisfactory ones, as we had done in our previous work.

**Figure 5:**
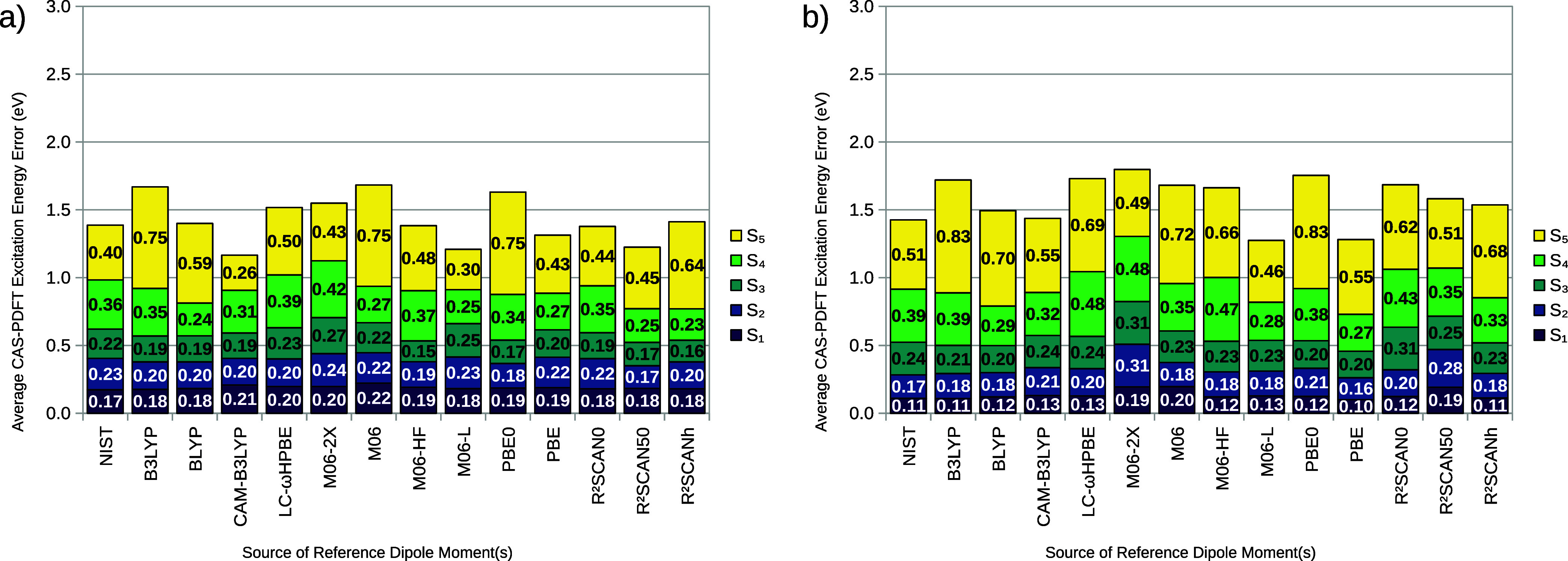
Low-lying (a) CAS-PDFT and (b) CASPT2 excitation energy errors calculated with active spaces chosen by CASCI-GDM-AS, averaged over all molecules in NAQ.

### CASCI-GDM-AS

S_1_–S_5_ CAS-PDFT and CASPT2 excitation energy errors with active spaces chosen by CASCI-GDM-AS, averaged over the molecules in NAQ, are presented in Figure 5. CASCI-GDM-AS produces satisfactory average excitation energies, both with CAS-PDFT and CASPT2, for the first three excited states S_1_ through S_3_. With CAS-PDFT, average excitation energy errors for all three of these states fall below our threshold of 0.3 eV, regardless of the source of reference dipole moment. Meanwhile, with CASPT2, the maximum average excitation energy error for these three states is 0.31 eV, barely above the threshold. These together suggest that CASCI-GDM-AS reliably chooses useful active spaces for finding CAS-PDFT or CASPT2 excitation energies for S_1_ through S_3_.

Average excitation energies for S_4_ and S_5_ are higher and generally do not fall below 0.3 eV. Most notably, all average CASPT2 excitation energy errors for S_5_ are at least 0.46 eV. There is not an apparent pattern in the sources of reference dipole moment for which average S_4_ or S_5_ excitation energy errors are satisfactory, although reference dipole moments from M06-L, PBE or CAM-B3LYP allow CASCI-GDM-AS to perform the best in this regard overall. In our previous work,^17^ we also found that GDM-AS based on CASSCF dipole moments only found satisfactory average excitation energy errors for S_1_ through S_3_ and was not reliable for S_4_ or S_5_. Similar to our previous work, there are much fewer reference data available at these higher excited states, so the performance of the protocols at these excited states in NAQ may not represent the performance of the protocols in a more general sense.

### CASCI-vGDM-AS

S_1_–S_5_ CAS-PDFT and CASPT2 excitation energy errors with active spaces chosen by CASCI-vGDM-AS, averaged over the molecules in NAQ, are presented in Figure 6. CASCI-vGDM-AS performs very similarly to CASCI-GDM-AS. All of the same observations for CASCI-GDM-AS—that average CAS-PDFT and CASPT2 excitation energy errors are satisfasctory for S_1_ through S_3_ and are usually not satisfactory for S_4_ or S_5_, and that M06-L, PBE and CAM-B3LYP reference dipole moments lead to the highest overall performance—are also seen with CASCI-vGDM-AS. The similarity in performance between CASCI-vGDM-AS and CASCI-GDM-AS might be due to the presence of molecules in the test data set with *C*_2*v*_ symmetry, for which the dipole moment is described in full by one Cartesian component. For these molecules, CASCI-GDM-AS and CASCI-vGDM-AS would be expected to perform similarly.

**Figure 6:**
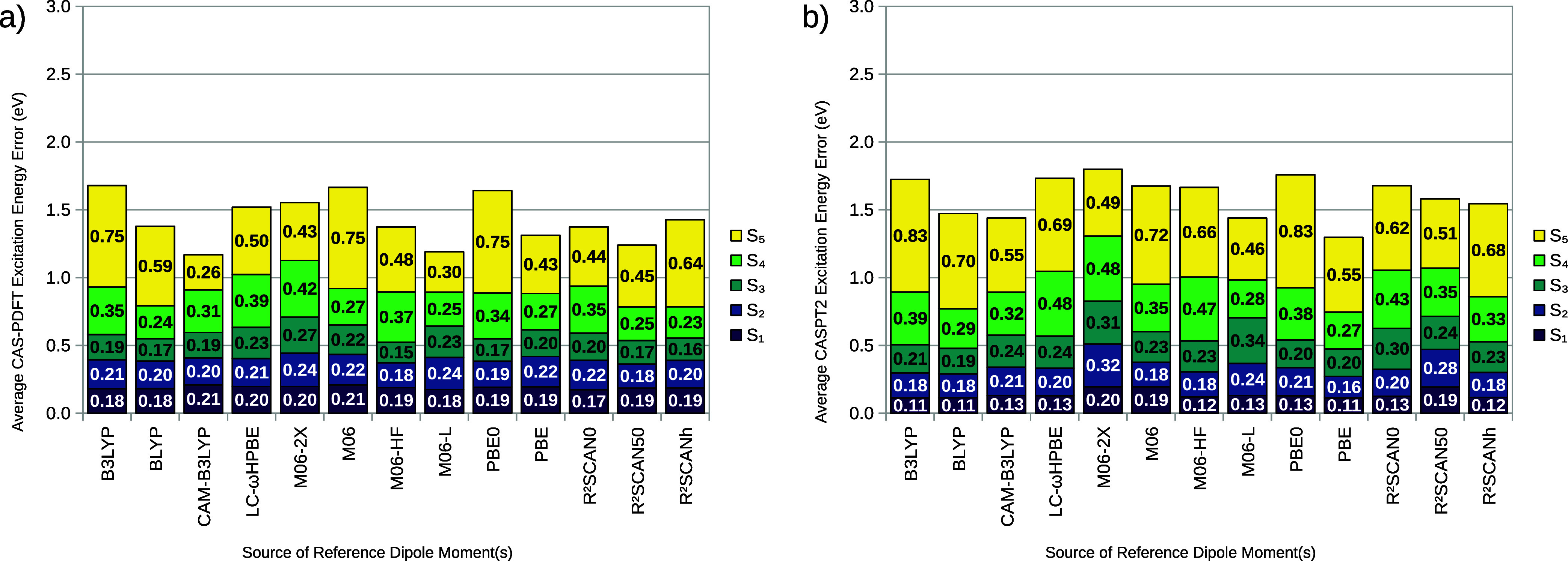
Low-lying (a) CAS-PDFT and (b) CASPT2 excitation energy errors calculated with active spaces chosen by CASCI-vGDM-AS, averaged over all molecules in NAQ.

### CASCI-EDM-AS

S_1_–S_5_ CAS-PDFT and CASPT2 excitation energy errors with active spaces chosen by CASCI-EDM-AS, averaged over the molecules in NAQ, are presented in Figure 7. For CASCI-EDM-AS, average CAS-PDFT excitation energy errors for S_1_ through S_3_ are satisfactory, regardless of the source of reference dipole moments. On the other hand, average CASPT2 excitation energy errors for S_1_ are not satisfactory (minimum 0.47 eV) when functionals in the R^2^SCAN family are used to provide reference dipole moments, while S_2_ excitation energy errors barely exceed the soft 0.3 eV threshold (minimum 0.32 eV). When reference dipole moments are provided by any other DFT functional besides those in the R^2^SCAN family, average CASPT2 excitation energy errors for S_1_ and S_2_ fall well within the threshold for satisfactory values, and average CASPT2 S_3_ excitation energy errors are always satisfactory for all sources of reference dipole moments.

**Figure 7:**
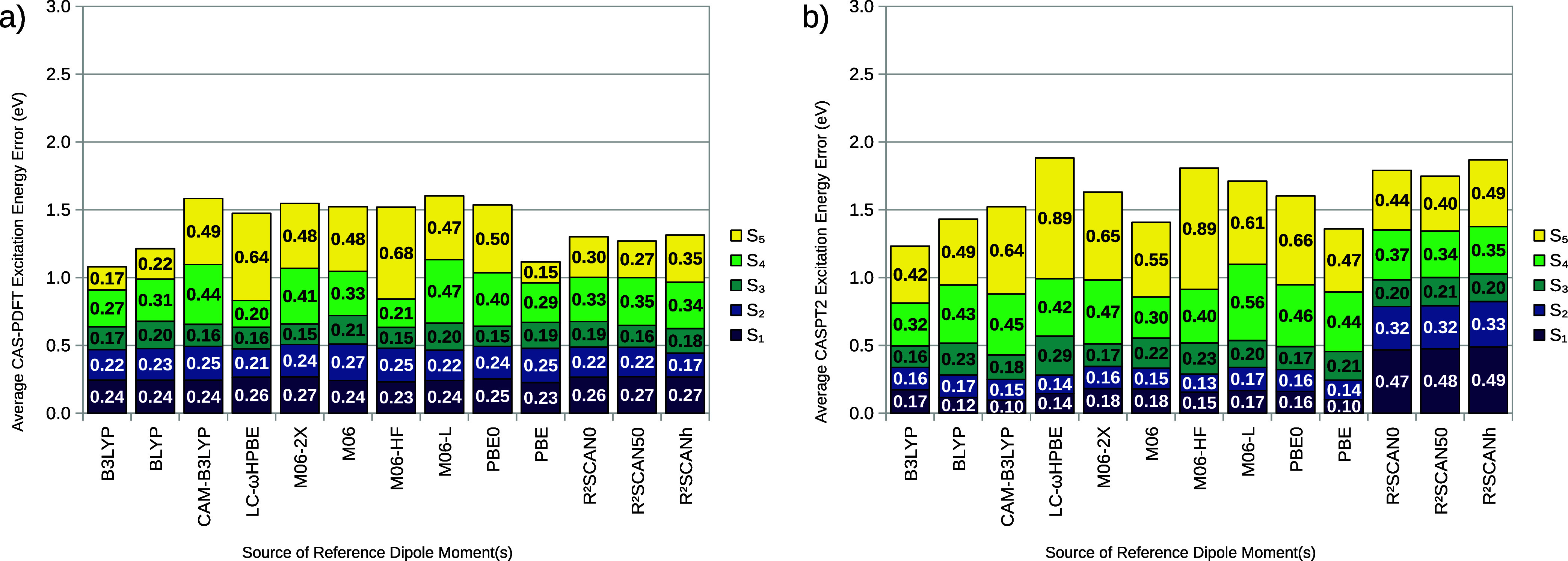
Low-lying (a) CAS-PDFT and (b) CASPT2 excitation energy errors calculated with active spaces chosen by CASCI-EDM-AS, averaged over all molecules in NAQ.

Average excitation energy errors for S_4_ and S_5_ are often not satisfactory, although the functionals B3LYP and PBE appear to the best for providing reference dipole moments in this regard.

### CASCI-vEDM-AS

S_1_–S_5_ CAS-PDFT and CASPT2 excitation energy errors with active spaces chosen by CASCI-vEDM-AS, averaged over the molecules in NAQ, are presented in Figure 8. Many of the same observations discussed with CASCI-EDM-AS are also seen with CASCI-vEDM-AS. Average S_1_ through S_3_ excitation energy errors are below, or close to, the 0.3 eV threshold except for average CASPT2 S_1_ excitation energy errors when DFT functionals in the R^2^SCAN family are used to provide reference dipole moments.

**Figure 8:**
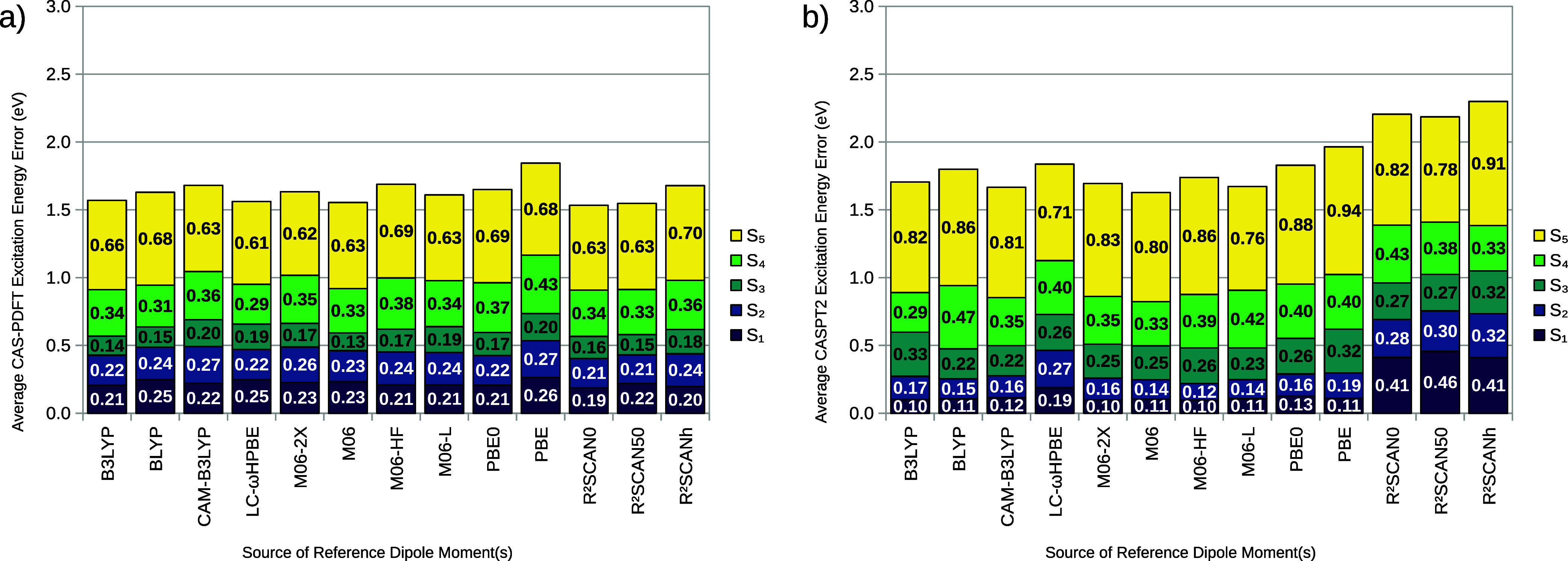
Low-lying (a) CAS-PDFT and (b) CASPT2 excitation energy errors calculated with active spaces chosen by CASCI-vEDM-AS, averaged over all molecules in NAQ.

However, there are some differences in the relative performance of specific sources of reference dipole moments, most notably with the PBE density functional. For CASCI-vEDM-AS, the total average CAS-PDFT excitation energy error for S_1_ through S_3_ is the largest of all sources of reference dipole moments tested when PBE is used to provide reference dipole moments; total average CASPT2 excitation energy errors for these states are the largest when the R^2^SCAN family is used to provide reference dipole moments and the second largest when PBE is used. For CASCI-EDM-AS, CASPT2 excitation energy errors were among the lowest when PBE was used to provide reference dipole moments. Otherwise, the influence of the source of reference dipole moments on the performance of CASCI-vEDM-AS is less pronounced than it is for CASCI-EDM-AS.

### CASCI-D^2^DM-AS

S_1_–S_5_ CAS-PDFT and CASPT2 excitation energy errors with active spaces chosen by CASCI-D^2^DM-AS, averaged over the molecules in NAQ, are presented in Figure 9. For CASCI-D^2^DM-AS, average CAS-PDFT excitation energy errors for S_1_ through S_3_ are satisfactory, regardless of the source of reference dipole moments. On the other hand, average CASPT2 excitation energy errors for S_1_ and S_2_ are not satisfactory (minimum 0.47 eV) when LC-ωHPBE or functionals in the R^2^SCAN family are used to provide reference dipole moments. When reference dipole moments are provided by any other DFT functional besides LC-ωHPBE or those in the R^2^SCAN family, average CASPT2 excitation energy errors for S_1_ and S_2_ fall well within the threshold for satisfactory values, or close to it (maximum 0.33 eV) and average CASPT2 S_3_ excitation energy errors are always satisfactory for all sources of reference dipole moments. The issue with using the R^2^SCAN family to provide reference dipole moments is similar to those in CASCI-EDM-AS and CASCI-vEDM-AS.

**Figure 9:**
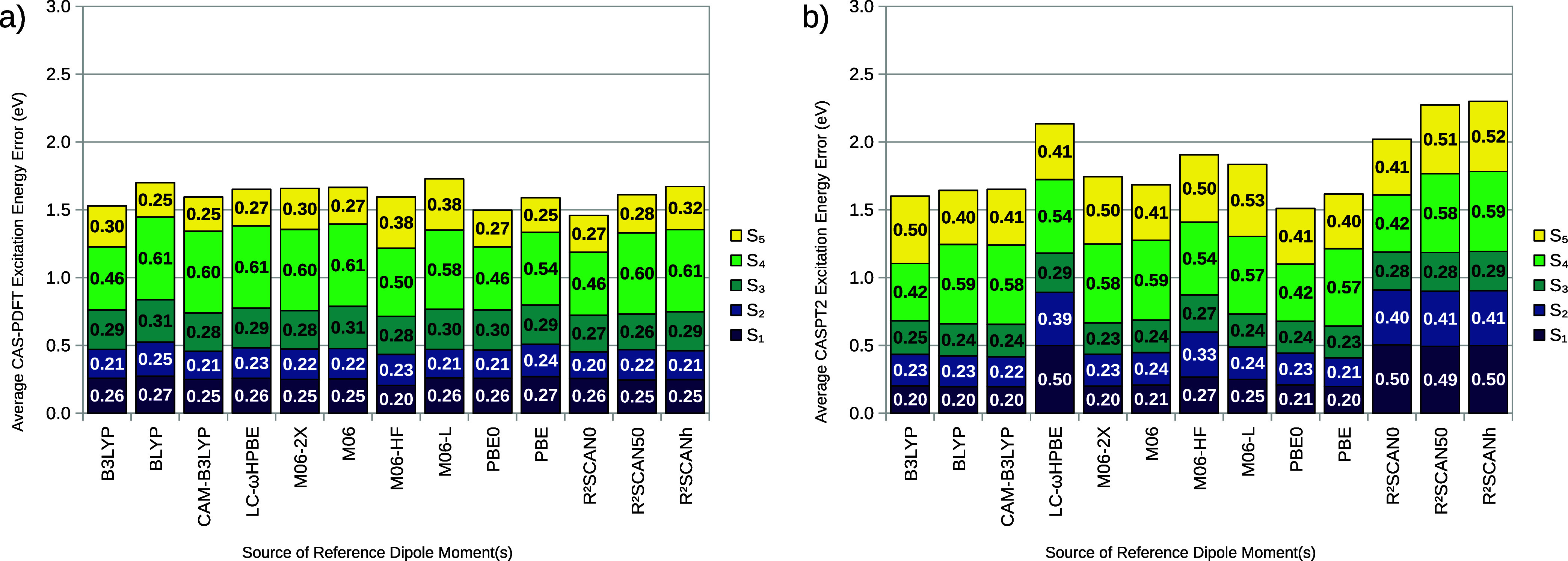
Low-lying (a) CAS-PDFT and (b) CASPT2 excitation energy errors calculated with active spaces chosen by CASCI-D^2^DM-AS, averaged over all molecules in NAQ.

Interestingly, average S_5_ excitation energies are better overall with CASCI-D^2^DM-AS than with any other protocol. Average CAS-PDFT S_5_ excitation energy errors are frequently below 0.3 eV. This does not indicate that there is a preference to select S_5_ as the state the protocol uses to select the active space, namely “the state of interest”. We arrive at this conclusion with a few analyses. First, for the six molecules for which reference data for the S_5_ excitation is available, for each active space we check the excited state selected for comparison to reference dipole moments. These are shown in the Supporting Information Tables S3–S8, and active spaces chosen with at least one source of reference dipole moments are shown in a box along with the CASCI excited state of interest for each of these active spaces. We see that S_5_ is only consistently chosen for comparison with reference dipole moments for two of the six molecules, and only chosen at all for four of the six molecules.

In addition, to verify that there is not a more widespread, inherent bias for CASCI-D^2^DM-AS to choose S_5_ as the state of interest, we have calculated the probability with which each CASCI excited state is chosen for comparison with the reference dipole moment for all of NAQ and all active spaces. These are found in the Supporting Information Table S2. We have found that S_5_ is indeed most likely to be chosen (24.60%) but only slightly more often than S_1_ (23.23%) and S_2_ (22.48%) and not significantly more often than it would be chosen by random chance (20%). The average is broken down into individual molecules and active spaces in the Supporting Information Tables S3–S25. These two analyses suggest that CASCI-D^2^DM-AS does not have an inherent propensity to choose S_5_ as the state of interest.

CASCI-D^2^DM-AS demonstrates a good ability to select the real charge transfer state for a molecule, if one is present, as the state of interest. There are five molecules in NAQ with charge transfer states listed in QUEST6,^30^ namely aniline, azulene, benzonitrile, hydrogen chloride and nitrobenzene. For four of these molecules, the real charge transfer state is considered the state of interest for at least one active space, with only aniline being the exception (Supporting Information Tables S3, S4, S10, S17, S22). Notably, for hydrogen chloride, every active space properly selects one of the two charge transfer states as the state of interest. For azulene and benzonitrile, which we analyzed in the set of NAQ molecules with reference data for the S_5_ excitation energy, a charge transfer state is considered the state of interest for at least one selected active space.

### CASCI-vD^2^DM-AS

S_1_–S_5_ CAS-PDFT and CASPT2 excitation energy errors with active spaces chosen by CASCI-GDM-AS, averaged over the molecules in NAQ, are presented in Figure 10. For CASCI-vD^2^DM-AS, average CAS-PDFT excitation energy errors for S_1_ through S_3_ are satisfactory, regardless of the source of reference dipole moments. On the other hand, average CASPT2 excitation energy errors for the first three excited states tend to be unsatisfactory. Of all the protocols tested in this work, CASCI-vD^2^DM-AS performs the worst overall for finding CASPT2 excitation energies. Interestingly, some of the sources of reference dipole moments for which CASCI-D^2^DM-AS performed worse—namely LC-ωHPBE, R^2^SCAN0 and R^2^SCAN50—enable CASCI-vD^2^DM-AS to perform better for finding CASPT2 excitation energies.

**Figure 10:**
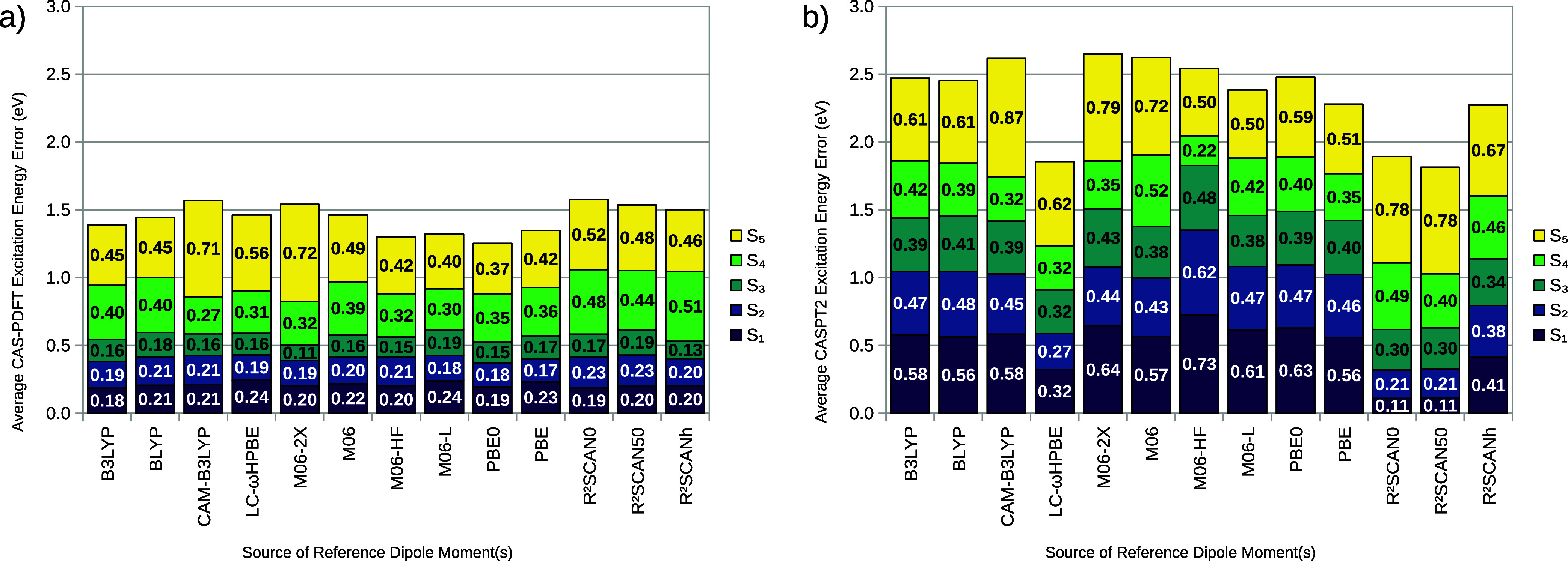
Low-lying (a) CAS-PDFT and (b) CASPT2 excitation energy errors calculated with active spaces chosen by CASCI-vD^2^DM-AS, averaged over all molecules in NAQ.

### General Discussion on the NAQ Data Set

All protocols excel at choosing active spaces for finding CAS-PDFT excitation energies for the first three excited states. Average excitation energy errors for these states are always within the 0.3 eV threshold, regardless of which reference dipole moments are used, with only two exceptions (average excitation energy errors for CASCI-D^2^DM-AS are 0.31 eV with BLYP or M06 reference dipole moments). Based on the hypothesis on which we built these dipole-moment-based protocols, we do expect that the protocols should work particularly well for finding CAS-PDFT excitation energies. When designing the protocols discussed in this work as well as our previous one,^17^ we operate on the principle that the active space which most accurately describes the dipole moment should also describe the electron density well. Since CAS-PDFT is highly dependent on the electron density, it is intuitive that CAS-PDFT calculations would benefit most from an active space that represents the electron density accurately. Both our current and previous^17^ results support that this hypothesis is correct.

Meanwhile, the performance of the protocols for choosing active spaces with which to calculate CASPT2 excitation energies depends more on the density functional used to compute the reference dipole moments. Note that we only see unsatisfactory CASPT2 excitation energy errors for protocols or sources of reference dipole moments which we did not test in our previous work when designing the protocols based on CASSCF dipole moments.^17^ The CASCI-D^2^DM-AS protocols in particular are entirely new to this work, and for the CASCI-EDM-AS protocols, we only see unsatisfactory CASPT2 excitation energies with sources of reference dipole moment we did not use when designing the protocols based on CASSCF dipole moments. It is possible that we are instead seeing features inherent to the dipole moment based protocols rather than a decrease in performance from using CASCI dipole moments specifically.

We found that average S_4_ and S_5_ excitation energy errors were frequently unsatisfactory, as we had with the protocols based on CASSCF dipole moments, which may be related to the fact that there are fewer reference values for those states.^17^ Nevertheless, we found that the average S_5_ excitation energies decreased substantially for CASCI-D^2^DM-AS, and for CASCI-EDM-AS with certain sources of reference dipole moment, which suggests that one may prefer to use either of these protocols when higher excitation energies are of interest. However, we treat all results regarding S_4_ and S_5_ excitation energies with caution, as there are few reference excitation energies in our data set for us to use to compare to our calculated excitation energies. In general, our results suggest that the protocols are reliable for choosing active spaces for finding the first three excitation energies.

By design, the selected active space chosen by the protocols is not limited to containing only valence orbitals which might be considered relevant by chemical intuition, and may contain Rydberg orbitals while omitting certain valence orbitals. This is intentional as “chemical intuition” can be wrong and does not necessarily result in reasonable or affordable active spaces. Rydberg orbitals may be necessary to describe even low-lying excitations in molecules,^29,77^ but systematic rules for including them in an active space are not available. The automated nature of the protocols allows for one to select useful active spaces within computational resource limitations, regardless of whether the orbitals can be classified to needed in the active space based on “chemical intuition”, without needing to consult systematic rules or deal with the lack thereof. One may argue that this approach compromises the interpretability of the active spaces, and can cause complications when one is seeking certain orbitals to be included in the active space to describe every geometry relevant in a specific chemical reaction. Regarding interpretability, we argue that interpretability can be limited to human’s existing knowledge, and our protocols have the capability to expand beyond known knowledge. Additionally, we view the active space selection problem to be analogous to selecting the starting condition and constraints of a nonconvex optimization problem, which by nature may be beyond human’s intuition. While we leave the comprehensive testing of our approach’s capability to deal with chemical reactions to future work, similar to our previous work,^17^ we tested the performance of the calculation of the potential energy curve for carbon monoxide, but with active spaces chosen at CO’s equilibrium bond length by the protocols CASCI-GDM-AS, which is (6,10), and CASCI-EDM-AS, which is (8,10). Potential energy curves calculated with these active spaces are presented in the Supporting Information Figure S36. We find that potential energy curves calculated with CASSCF, CASPT2 and CAS-PDFT at these active spaces agree well with the improved extended-Lennard-Jones curve presented in the work of Araújo et al.^78^ This suggests that for a simple chemical reaction like the bond dissociation of CO, the active space chosen at the equilibrium geometry can still describe other geometries on the reaction coordinate.

CASCI-(v)­EDM-AS and CASCI-(v)­D^2^DM-AS depend on the presence of nonzero excited-state dipole moments in order to make the active space selection, with CASCI-(v)­D^2^DM-AS in particular depending on the presence of an excited state with a dipole moment significantly different from the ground state. Despite this, these protocols are capable of choosing active spaces that accurately model dark states. We establish this by applying these protocols to the NAQ data set, and only calculating excitation energy errors for excited states with an oscillator strength listed as 0.000 in QUEST. The data set of excitations used for this analysis is provided in the Supporting Information Figure S7 and the results of applying the protocols are given in the Supporting Information Figures S8–S11. For all protocols and sources of reference dipole moment, average S_1_ and S_2_ excitation energy errors are always satisfactorily low both with CAS-PDFT and CASPT2 and are not higher than the average excitation energy errors when bright states are considered. Only average S_3_ excitation energies exceed 0.3 eV in some cases; however, one must mind the fact that the sample size of dark S_3_ excitations is only one among all molecules.

### Conjugated Polar Molecules

The six protocols in this work were tested against a set of 13 conjugated polar molecules in the QUEST^29–31^ database shown in the Supporting Information Figure S1. The average CAS-PDFT and CASPT2 excitation energy errors for the data set, for active spaces selected with the protocols, are given in the Supporting Information Figures S12–S17.

Results suggest that the protocols are useful for modeling the first two low-lying excitations. Average S_1_ and S_2_ excitation energy errors, both for CAS-PDFT and CASPT2, are often satisfactory (≤0.3 eV). Although this threshold is exceeded for some protocols and sources of reference dipole moment, average errors with CAS-PDFT tend not to exceed it to a large degree; the maximum average S_1_ CAS-PDFT excitation energy error for any protocol and source of reference dipole moments is 0.39 eV, while for S_2_, it is 0.38 eV. Meanwhile, average excitation energy errors for S_3_ through S_5_ are less reliable, and can reach values above 1 eV.

Large average S_1_ excitation energy errors in excess of 1 eV are occasionally seen with CASPT2, particularly for CASCI-vD^2^DM-AS (when CAM-B3LYP, M06-2X, M06-HF and R^2^SCANh are used to provide reference dipole moments) and CASCI-vEDM-AS (when R^2^SCAN50 is used to provide reference dipole moments). Evidently, these large errors are caused by a single outlier. For the maleimide molecule, the (8,7) active space has an unusually large S_1_ CASPT2 excitation energy error of 7.39 eV; this active space is chosen with all protocols and sources of reference dipole moment for which large average S_1_ excitation energy errors are observed. The CASPT2 calculation for maleimide with this active space shares some similarities to our erroneous CASPT2 calculation with difluorocarbene, in particular that CASPT2 required many iterations to converge for some roots, and the absolute energy of a higher root dropped below that of S_0_ after CASPT2 corrections to CASSCF. This, combined with the fact that we never see unusually high average S_1_ CAS-PDFT excitation energy errors, and that some of these convergence issues, as demonstrated in difluorocarbene, can be resolved by using ORCA instead of OpenMolcas, suggests that the issue may be a CASPT2-specific problem in OpenMolcas and not an issues of the active space selection protocols. The largest average S_2_ CASPT2 excitation energy error observed is 0.49 eV.

Overall, these results show that the protocols discussed in this work offer promise as active space selection protocols for modeling excitation energies of conjugated polar molecules.

### Nonpolar Systems

Four protocols in this work were tested against a set of 5 nonpolar molecules from the QUEST^29–31^ database shown in the Supporting Information Figure S2. The average CAS-PDFT and CASPT2 excitation energy errors for the data set, for active spaces selected with the protocols, are given in the Supporting Information Figures S18–S21. CASCI-EDM-AS, CASCI-D^2^DM-AS, and their directional counterparts were tested as these protocols allow for the use of the dipole moments of excited states, which can be nonzero even if the ground-state dipole moment of the molecule is zero. CASCI-GDM-AS and CASCI-vGDM-AS explicitly depend on nonzero ground-state dipole moments so they were not applied to this data set.

Results for this data set show greater excitation energy errors than the NAQ data set and the conjugated polar molecules previously discussed. Nevertheless, the active spaces chosen by the protocols do appear to be useful for calculating S_1_ excitation energies with CASPT2, with the highest average error being 0.45 eV for all protocols and sources of reference dipole moment. CAM-B3LYP appears to be the best functional to use for reference dipole moments across all protocols when CASPT2 excitation energies are sought, with the average S_1_ excitation energy error not exceeding 0.39 eV for any protocol, and the total average excitation energy error for all five low-lying excitations being among the lowest for all protocols. When CAS-PDFT excitation energies are sought, CAM-B3LYP reference dipole moments are still among the most useful (highest S_1_ average excitation energy error of 0.61 eV), along with those from LC-ωHPBE (0.58 eV). The directional versions, CASCI-vEDM-AS and CASCI-vD^2^DM-AS, can perform reasonably well for CASPT2 S_2_ excitation energy as well. However, CASPT2 excitation energies for S_3_ and above, and CAS-PDFT excitation energy errors for all low-lying excitations, are not consistently modeled accurately. As such, cautions need to be taken when using the protocols for nonpolar systems. We leave adapting the protocols for improved performance for nonpolar molecules to future work.

The specification of CASCI-EDM-AS and CASCI-vEDM-AS naturally leads to the possibility that they perform worse when a nonpolar system is studied. For both of these protocols, the difference in the CASCI and reference (TD-DFT in this work) dipole moment for all low-lying excitation energies are used in the active space selection process. If, for numerous excited states, the dipole moment is zero for either CASCI or TD-DFT but not both (say, if the CASCI and TD-DFT excited states are not in the same order), unnaturally high differences in dipole moment may affect active space selection.

CASCI-D^2^DM-AS and CASCI-vD^2^DM-AS are designed to use only the excited state with the dipole moment most different from the ground state for active space selection, so in theory should avoid the issue with CASCI-(v)­EDM-AS discussed above. It is notable that for CASCI-vD^2^DM-AS, average S_2_ CAS-PDFT excitation energy errors are always less than 0.3 eV, while the corresponding CASPT2 average errors are satisfactory for all sources of reference dipole moment other than M06-2X and M06-HF. As for CASCI-D^2^DM-AS, average excitation energies are frequently low for S_1_, S_3_ and (only for CASPT2) S_5_, suggesting that this protocol does have some benefit over CASCI-(v)­EDM-AS.

### Molecules with Transition Metal Atoms

CASCI-GDM-AS was applied to the selection of active spaces for the determination of ground-state spin states for a set of five transition metal monoxides. These five molecules, ScO, TiO, VO, CrO and MnO, are chosen from the work by Wagner and Mitas.^40^ Here, only CASCI-GDM-AS was applied because only ground-state properties were of interest. CASCI dipole moments were compared to reference values in Wagner and Mitas^40^ which are as follows:

- ScO, VO, CrO: experimental values from the work of Steimle.^79^
- TiO: experimental values from the work of Huang et al.^80^
- MnO: Quantum Monte Carlo values from the work of Wagner and Mitas^40^

For molecules with an even number of electrons, we use the set of active spaces constrained by $\frac{n_{e}}{2}\in N$ , 6 ≤ *n*_e_ ≤ 14, $n_{o}\in N$ , $\frac{n_{e}}{2}+3\leq n_{o}\leq 14$ as candidates. For molecules with an odd number of electrons, we use the set of active spaces constrained by $\frac{n_{e}-1}{2}\in N$ , 5 ≤ *n*_e_ ≤ 13, $n_{o}\in N$ , $\frac{n_{e}+1}{2}+3\leq n_{o}\leq 14$ as candidates. Also, due to a limitation in OpenMolcas preventing the use of unrestricted MP2, unrestricted M06-2X was used instead to produce starting orbitals for CASCI.

Relative ground-state energies of the transition metal monoxides at various spin multiplicities are provided in the Supporting Information Tables S26–S28. Results indicate that CASCI-GDM-AS is useful for choosing active spaces for determining ground-state spin states for these transition metal monoxides. With active spaces chosen by CASCI-GDM-AS, the energy of the correct ground-state spin state is always the lowest, whether the energies are calculated with CASSCF, CASPT2 or CAS-PDFT. This demonstrates that the protocols can be applied to finding properties other than excitation energies and are not limited to organic molecules.

### Bimolecular Complexes

The protocols discussed in this work were tested against a set of seven bimolecular complexes chosen from the work of Kozma et al.^41^ Coordinates, and reference excitation energies at the CCSDT/aug-cc-pVDZ level or the CCSDT-3/aug-cc-pVDZ level, are obtained from Kozma et al.^41^ In order to model higher-energy excitations that may be charge-transfer in nature, we increased the number of roots modeled with CASCI and CASSCF from 6 to 11, and as such, the number of excited states from 5 to 10. Due to the large size of the complexes, CASSCF was run differently. First, a CASSCF calculation with the resolution-of-identity (RI) approximation and an auxiliary basis set generated as specified in Aquilante et al.^81^ was run to produce a starting wave function for a higher-quality CASSCF calculation. Then, using the resulting wave function, a second CASSCF calculation was run without the RI approximation. OpenMolcas 21.06 was used in place of OpenMolcas 22.02 for this data set.

The Supporting Information Figure S3 shows the molecules in this dataset while the Supporting Information Figures S22-S27 show the results of applying the protocols to this dataset. Results indicate that the protocols seem capable of properly choosing active spaces for modeling excitation energies in bimolecular complexes, but can have large errors for some protocols and sources of reference dipole moments. Average S_1_ CASPT2 excitation energy errors tend to be satisfactory, with some exceptions. The average excitation energy errors for higher excited states are not consistently satisfactory. However, it is interesting to note that there are specific average excitation energy errors that suggest that the active space chosen by the protocol is well-suited for modeling higher excitation energies in specific cases. For example, average S_3_ CAS-PDFT excitation energies are usually satisfactory when CASCI-GDM-AS is used for active space selection, and average S_8_ errors are also lower when CASCI-vGDM-AS is used for active space selection for CAS-PDFT or CASCI-vD^2^DM-AS is used for active space selection for CASPT2. As such, the protocols seem to have the potential of performing well with bimolecular systems, and we leave further development to future work.

### Comparison of Run Time

The active space selection protocols based on CASCI dipole moments are much more computationally economical than those based on CASSCF dipole moments. Because CASCI does not involve orbital optimization while CASSCF does, we expect that a CASCI calculation to be almost always faster than a CASSCF calculation for the same system, active space, and computational resources, given that the starting orbital is not from CASSCF. We confirm this by comparing the run times of the calculations required for the active space selection step of the protocols tested in this work to those based on CASSCF dipole moments, as shown in Figure 11, for all molecules and active spaces tested in this work, including the molecules in NAQ, the conjugated polar molecules, and the nonpolar molecules to make this comparison. Run time data for bimolecular complexes and transition metal monoxides were plotted separately owing to procedural differences with these data sets, most prominently different numbers of roots from other molecules. Relevant plots for these other data sets are provided in the Supporting Information Figures S30–S32 for bimolecular complexes, and Figures S33–S35 for transition metal monoxides.

**Figure 11:**
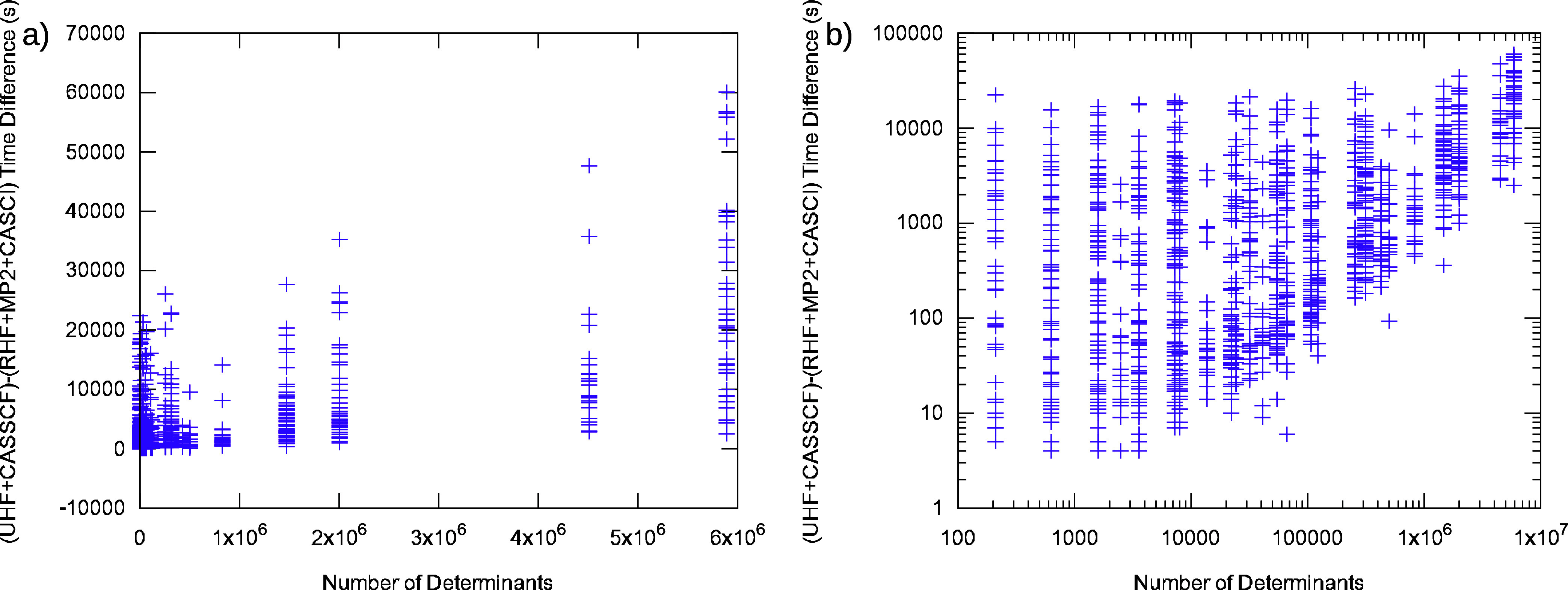
Difference in combined run times of UHF + CASSCF, and MP2 + CASCI (this work), for molecules in NAQ, as well as conjugated polar and nonpolar molecules. (a) Linear axes; (b) log–log axes.

In order to account for the time required to build the starting orbitals for the multireference calculations, we follow the following steps to generate Figure 11:

1. Add the wall time, in seconds, for UHF to that of CASSCF. Refer to this as *u*.
2. Add the wall time for MP2 (itself a sum of separate run times for the &SCF and &MP2 modules) to that of CASCI. Refer to this as *m*.
3. Plot (*u* – *m*) with respect to the number of determinants, as reported by OpenMolcas.

It is clear that the active-space selection based on CASSCF dipole moments has greater run times than the active-space selection based on CASCI dipole moments for almost all calculations, even when the run time for producing starting orbitals is accounted for. The average (*u* – *m*) value for all calculations is 3252.87 s, with a maximum value of 60,078 s and a minimum value of −12 s. As such, in the best case scenario for this data set, replacing CASSCF with CASCI at the active space selection phase saved 16.69 h, while in the worst case, it added 12 s of run time. This suggests that the efficiency benefit of using CASCI over CASSCF dipole moments far outweighs the risk.

Additionally, the time difference (*u* – *m*) is seen to increase with the number of determinants. This indicates that the efficiency benefit for using CASCI over CASSCF is greatest for the most resource-intensive calculations, where the increase in efficiency is more critical.

To verify that a time savings for choosing MP2 and CASCI over UHF and CASSCF exists independent of the absolute run time of the calculations, we also provide plots of the ratio $\frac{u}{m}$ with respect to the number of determinants in the Supporting Information Figure S28. Since $\frac{u}{m}>1$ for almost all calculations, it is advantageous to use MP2 and CASCI over UHF and CASSCF for faster run times. $\frac{u}{m}<1$ occasionally due to the absolute run time being very small for some cases, and factors such as the CPU load can significantly impact a run time ratio. At the maximum, UHF + CASSCF runtime is 35.01 times that of MP2 + CASCI. Additionally, $\frac{u}{m}$ tends to be higher with a larger number of determinants. It again shows that the efficiency benefit for using CASCI over CASSCF is greater for the more resource-intensive calculations.

To understand the time saved by using CASCI instead of CASSCF, we also plot the ratio of the run time of CASSCF calculations to the run time of CASCI calculations, without adding the respective run times for producing starting orbitals, with respect to the number of determinants. The relevant plots are provided in the Supporting Information Figure S29. Results are similar to those with the run times for producing starting orbitals included, with the maximum ratio over 45. Again, some CASSCF/CASCI run time ratios are less than 1. Similar to the case for $\frac{u}{m}$ , all such cases are for the smallest molecule in NAQ (carbon monoxide) with small active spaces, where run times are in the range of tens of seconds or less. The runtime of these short calculations are more susceptible to factors such as CPU load.

The same analyses of the run time difference and ratio between CASSCF and CASCI are provided in the Supporting Information Figures S30–S32 for bimolecular complexes, and Figures S33–S35 for transition metal monoxides. The observations are similar to those for the combined NAQ, conjugatedpolar, and nonpolar data sets, with CASSCF/CASCI run time ratios in the same order of magnitude and increase with respect to the number of determinants. Additionally, they have a greater maximum CASSCF/CASCI run time ratio (over 50 for bimolecular complexes and over 80 for transition metal oxides) and a more prominent increase of the ratio as a function of the number of determinants than a smaller system like those in the NAQ data set.

### Recommendations

For finding CAS-PDFT excitation energies for the first three excited states, all active space selection protocols perform similarly. They are generally best with directional CASCI-D^2^DM-AS (CASCI-vD^2^DM-AS), so a user may wish to choose this protocol when maximum accuracy is desired. CASCI-GDM-AS and CASCI-vGDM-AS perform nearly as well and might be desirable for their simpler implementation. CASCI-GDM-AS and CASCI-vGDM-AS are also more efficient, as the calculation of excited-state reference dipole moments is not required, and if the ground-state reference dipole moment is determined experimentally, calculations to obtain reference dipole moment can be completely bypassed.

For finding CASPT2 excitation energies for the first three excited states, CASCI-EDM-AS has the best performance provided that density functionals in the R^2^SCAN family are not used to provide reference dipole moments, and may be chosen if maximum accuracy is desired. CASCI-vEDM-AS, CASCI-GDM-AS, and CASCI-vGDM-AS have slightly greater average excitation energy errors than CASCI-EDM-AS, but they are usually still satisfactory, providing average CASPT2 excitation energy errors much smaller than these protocols for CAS-PDFT excitation energy errors. Due to the simpler implementation of CASCI-GDM-AS and CASCI-vGDM-AS, they may be used for maximum efficiency (due to bypassing the calculation of excited-state reference dipole moments) that balances accuracy. The excitation energy errors averaging over all molecules studied in this work does not show a benefit of using CASCI-D^2^DM-AS or CASCI-vD^2^DM-AS over the other protocols for CASPT2 excitation energies, and CASCI-vD^2^DM-AS gives particularly problematic average excitation energy errors. That said, CASCI-D^2^DM-AS can still provide CASPT2 excitation energy errors similar to that for CAS-PDFT excitation energy errors.

When it comes to molecules with charge transfer states, however, CASCI-D^2^DM-AS and CASCI-vD^2^DM-AS show an advantage for both CASPT2 and CAS-PDFT excitation energies compared to an arbitrary molecule in the data set. For example, for azulene, S_0_ → S_2_ and S_0_ → S_3_ are generally considered CT. CASCI-D^2^DM-AS gives a CASPT2 excitation energy error of 0.04, 0.18, and 0.05 eV, for S_1_, S_2_, and S_3_, respectively, from using reference dipole moments from any of the density functionals tested other than LC-ωHPBE, which gives an even smaller excitation energy error which is 0.07, 0.00, and 0.06 eV, respectively, and M06-HF, which gives a much greater excitation energy error which is 1.51, 1.60, and 0.51 eV, respectively. Other than using the M06-HF reference dipole moment, CASCI-D^2^DM-AS has excellent performance for azulene compared to Figure 9. CASCI-vD^2^DM-AS gives a CASPT2 excitation energy error of 0.09, 0.15, and 0.07 eV, for S_1_, S_2_, and S_3_, respectively, from using reference dipole moments from any of the density functionals tested other than those in the R^2^SCAN family, which gives an even smaller excitation energy error which is 0.09, 0.13, and 0.07 eV, respectively. Again, this is much better performance than the average performance shown in Figure 10. For CAS-PDFT excitation energies of azulene, CASCI-D^2^DM-AS and CASCI-vD^2^DM-AS offer a clear improvement for S_2_ and S_3_ excitation energies over the non-CT states, where S_1_ has similar performance to the average performance in Figures 9 and 10. With CASCI-D^2^DM-AS, CAS-PDFT excitation energy errors for S_2_ and S_3_ are 0.03 and 0.06 eV, respectively, from using reference dipole moments from any of the density functionals tested other than LC-ωHPBE (0.35 eV, 0.15 eV) and M06-HF (0.06 eV, 0.12 eV). With CASCI-vD^2^DM-AS, CAS-PDFT excitation energy errors for S_2_ and S_3_ are 0.06 and 0.12 eV, respectively, from using reference dipole moments from any of the density functionals tested other than the R^2^SCAN family (0.25 eV, 0.05 eV). These results suggest that one can prioritize the use of CASCI-D^2^DM-AS or CASCI-vD^2^DM-AS when dealing with molecules with charge transfer states.

There is no clear evidence that using a directional variant of the active space selection protocols (e.g., CASCI-vGDM-AS and CASCI-vEDM-AS) has any significant benefit over using a nondirectional variant (CASCI-GDM-AS and CASCI-EDM-AS). Average excitation energy errors for S_1_ through S_3_ are about equal between the directional and nondirectional variants. If a user faces the decision of using CASCI-GDM-AS vs CASCI-vGDM-AS (or CASCI-EDM-AS vs CASCI-vEDM-AS) for their application, they can choose to use the nondirectional variants owing to their simplicity. This also suggests that we do not need to attempt to experimentally resolve the dipole moments into individual spatial components, if we choose to use reference dipole moments from experimental measurements.

All of the above recommendations are based on our observations for the NAQ data set, and are most likely applicable to organic molecules similar to those in NAQ. When dealing with other cases like those in the additional data sets, some recommendations change. CASCI-vD^2^DM-AS is still generally a top performer when CAS-PDFT excitation energies are of interest, and it is also true that no protocol stands out as unequivocally, significantly better than any other, so CASCI-(v)­GDM-AS may still be attractive for their simplicity. Also, there does not seem to be a consistent benefit to using the directional variant of the protocols over their nondirectional variants. However, when low-lying CASPT2 excitation energies are sought, CASCI-EDM-AS no longer stands out as a particularly high-performance method, unlike the NAQ data set. In general, no one protocol is likely to be the best or worst for a general data set. As such, users will probably opt for the simplest protocol that the application allows, making CASCI-GDM-AS a good “default” choice unless there is a specific reason to select a different one or the user is interested in testing/developing the protocols further.

For a potential energy scan of an arbitrary molecule, the protocols do not offer any theoretical guarantee that the active spaces chosen for every geometry will be the same, as the protocols are designed mainly to screen out unreasonable active spaces. Nevertheless, it is possible to select the active space for only one geometry and use the selected active space for all geometries along the potential energy surface. Whether this holds for exploring a complex potential energy surface of a large and complex molecular system is left to future work.

## Future Directions

In this work, we did not perform calculations on charged systems as the dipole moment depends on the definition of the origin of the Cartesian coordinates for charged molecules. It is theoretically possible to use these protocols to choose active spaces for charged systems provided that the same origin is used for all calculations involved in the active space selection process. However, the protocols might not be useful if the user is obtaining reference dipole moment from experimental data.

Another possibility for using the protocols for systems outside the purview of the ones in this work is to use another property besides the dipole moment for active space selection, such as the quadrupole moment. We have chosen the dipole moment in our work because it is a physical observable directly related to the electron density and thus the wave function, and the user has the option to obtain reference data from experiments instead of calculations. We envision that the protocols described in this work can be adapted for the formulation of other active space selection protocols which use the same workflow of comparing a property computed with CASCI with different active spaces to some reliable reference that can be easily obtained, using properties other than the dipole moment.

## Conclusion

We have designed a set of active space selection protocols, CASCI-GDM-AS, CASCI-EDM-AS, CASCI-D^2^DM-AS, and their directional variants, which use dipole moments calculated with CASCI to select active spaces for finding low-lying excitation energies with CAS-PDFT or CASPT2 with reasonable accuracy and efficiency. In our previous work, we had already demonstrated the potential for our active space selection protocols based on CASSCF dipole moments to reduce the time-to-solution, avoid human error, and adapt to a user’s computational resources. In this work, we have demonstrated that it is possible to improve the efficiency of these protocols even further, by replacing the CASSCF calculations with the less computationally expensive CASCI method in choosing the active space. The CASCI-based protocols demonstrate significantly greater improvement in efficiency as the active space becomes larger.

Like their counterparts based on CASSCF dipole moments, the active space selection protocols based on CASCI dipole moments presented in this work prevent the user from running expensive calculations with large active spaces which might not be necessary. For the CASSCF-based protocols, CAS-PDFT and CASPT2 are only run for the single active space chosen from candidate active spaces, and these active spaces do not necessarily include the largest active space that can be afforded. With the CASCI-based protocols, in addition to this benefit, CASSCF is only run for the single chosen active space instead of all the candidate active spaces.

Additionally, in this work, we designed new protocols, CASCI-D^2^DM-AS and its directional variant CASCI-vD^2^DM-AS, that make use of states with maximal dipole moment changes compared to the ground state to prioritize the description of charge transfer states, which appear to be particularly suitable for molecules with charge transfer states.

The performance of all protocols discussed here for finding low-lying CAS-PDFT excitation energies supports the validity of the underlying hypothesis behind the design of the protocols, that an active space which accurately represents the dipole moment should also represent the electron density reasonably. This opens the door for other active space selection protocols to be designed which make use of molecular properties that represent the electron density.

The protocols additionally show promise in dealing with active space selection for calculating excitation energies of larger and more complex molecules, such as those with extensive conjugation, either polar or nonpolar, and bimolecular complexes, which are systems not tested in our previous work on CASSCF-dipole-moment-based protocols. Among these, conjugated polar molecules perform the best. The results for nonpolar molecules and bimolecular complexes exceed the expectation, and we leave further development to improve their results to future work. The protocols are also capable of predicting the ground-state spin states of transition metal complexes, as well as obtaining a reasonable bond dissociation curve for CO. Comprehensive testing and further development for exploring the potential energy surfaces of complex molecules’ chemical reactions would be an interesting future direction. In terms of computational time, we demonstrated that there is an efficiency benefit to using CASCI over CASSCF for all these data sets in the active space selection process.

In summary, in this work, we demonstrate a major improvement of the efficiency of our dipole-moment-based active-space selection protocols. Our new protocols will enable our automated active space selection to be applied to even larger systems with less computational resources than previously demonstrated. It also shows that CASCI (as opposed to the more computationally expensive CASSCF) can be a reasonable starting point for active space selection in general, beyond this work. Furthermore, it demonstrates the strength of making use of physical observables as descriptors to select the active space, as one can further customize the protocols based on the specific physical properties of interest (e.g., charge transfer).

## Supplementary Material



## Notes

Excitation energies for difluorocarbene calculated with OpenMolcas and ORCA; Cartesian coordinates of molecules in the NAQ data set; structures of molecules and complexes in the additional data sets; benchmarking data for determining starting orbitals for use with CASCI; excited states chosen as the state of interest with the CASCI-D^2^DM-AS protocol; average excitation energy errors for dark states with active spaces selected by protocols; protocol performance with the additional data sets; ratios of CASSCF and CASCI run time for NAQ; run time data for the additional data sets; potential energy curves for carbon monoxide with selected active spaces (PDF)

## References

[1] Eade R. H. A., Robb M. A. (1981). Direct minimization in mc scf theory. the quasi-newton
method. Chem. Phys. Lett..

[2] Roos B. O., Taylor P. R., Sigbahn P. E. M. (1980). A complete active
space SCF method
(CASSCF) using a density matrix formulated super-CI approach. Chem. Phys..

[3] Hegarty D., Robb M. A. (1979). Application of unitary
group methods to configuration
interaction calculations. Mol. Phys..

[4] Bao J. J., Dong S. S., Gagliardi L., Truhlar D. G. (2018). Automatic Selection
of an Active Space for Calculating Electronic Excitation Spectra by
MS-CASPT2 or MC-PDFT. J. Chem. Theory Comput..

[5] Bao J. J., Truhlar D. G. (2019). Automatic Active Space Selection
for Calculating Electronic
Excitation Energies Based on High-Spin Unrestricted Hartree–Fock
Orbitals. J. Chem. Theory Comput..

[6] Lei Y., Suo B., Liu W. (2021). iCAS: Imposed Automatic Selection
and Localization
of Complete Active Spaces. J. Chem. Theory Comput..

[7] Stein C. J., Reiher M. (2019). autoCAS: A Program for Fully Automated
Multiconfigurational
Calculations. J. Comput. Chem..

[8] Jeong W., Stoneburner S. J., King D., Li R., Walker A., Lindh R., Gagliardi L. (2020). Automation of Active Space Selection
for Multireference Methods via Machine Learning on Chemical Bond Dissociation. J. Chem. Theory Comput..

[9] Sayfutyarova E. R., Sun Q., Chan G. K.-L., Knizia G. (2017). Automated Construction of Molecular
Active Spaces from Atomic Valence Orbitals. J. Chem. Theory Comput..

[10] Sayfutyarova E. R., Hammes-Schiffer S. (2019). Constructing Molecular π-Orbital Active Spaces
for Multireference Calculations of Conjugated Systems. J. Chem. Theory Comput..

[11] Golub P., Antalik A., Veis L., Brabec J. (2021). Machine Learning-Assisted
Selection of Active Spaces for Strongly Correlated Transition Metal
Systems. J. Chem. Theory Comput..

[12] King D. S., Gagliardi L. (2021). A Ranked-Orbital Approach to Select
Active Spaces for
High-Throughput Multireference Computation. J. Chem. Theory Comput..

[13] Bao J. L., Sand A., Gagliardi L., Truhlar D. G. (2016). Correlated-Participating-Orbitals
Pair-Density Functional Method and Application to Multiplet Energy
Splittings of Main-Group Divalent Radicals. J. Chem. Theory Comput..

[14] Pulay P., Hamilton T. P. (1988). UHF natural orbitals for defining and starting MC-SCF
calculations. J. Chem. Phys..

[15] Khedkar A., Roemelt M. (2019). Active Space Selection Based on Natural Orbital Occupation
Numbers from n-Electron Valence Perturbation Theory. J. Chem. Theory Comput..

[16] Khedkar A., Roemelt M. (2020). Extending the ASS1ST Active Space Selection Scheme
to Large Molecules and Excited States. J. Chem.
Theory Comput..

[17] Kaufold B. W., Chintala N., Pandeya P., Dong S. S. (2023). Automated Active
Space Selection with Dipole Moments. J. Chem.
Theory Comput..

[18] Knowles P., Handy N. (1984). A new determinant-based
full configuration interaction method. Chem.
Phys. Lett..

[19] Szabo, A. ; Ostlund, N. S. Modern Quantum Chemistry: Introduction to Advanced Electronic Structure Theory; Dover Publications, Inc: Mineola, NY, 2012.

[20] Helgaker, T. ; Jørgensen, P. ; Olsen, J. Molecular Electronic-Structure Theory, 1st ed.; Wiley, 2000

[21] Møller C., Plesset M. S. (1934). Note on
an Approximation Treatment for Many-Electron
Systems. Phys. Rev..

[22] Frisch M. J., Head-Gordon M., Pople J. A. (1990). A direct MP2 gradient method. Chem. Phys. Lett..

[23] Frisch M. J., Head-Gordon M., Pople J. A. (1990). Semi-direct algorithms for the MP2
energy and gradient. Chem. Phys. Lett..

[24] Head-Gordon M., Pople J. A., Frisch M. J. (1988). MP2 energy
evaluation by direct methods. Chem. Phys. Lett..

[25] Sæbø S., Almlöf J. (1989). Avoiding the
integral storage bottleneck in LCAO calculations
of electron correlation. Chem. Phys. Lett..

[26] Head-Gordon M., Head-Gordon T. (1994). Analytic MP2
frequencies without fifth-order storage.
Theory and application to bifurcated hydrogen bonds in the water hexamer. Chem. Phys. Lett..

[27] Levine B. G., Durden A. S., Esch M. P., Liang F., Shu Y. (2021). CAS without
SCF-Why to use CASCI and where to get the orbitals. J. Chem. Phys..

[28] NIST Computational Chemistry Comparison and Benchmark Database, NIST Standard Reference Database Number 101; Johnson, R. D. , Ed.; NIST, 2022.

[29] Véril M., Scemama A., Caffarel M., Lipparini F., Boggio-Pasqua M., Jacquemin D., Loos P.-F. (2021). QUESTDB: A database
of highly accurate excitation energies for the electronic structure
community. WIRES Comput. Mol. Sci..

[30] Loos P.-F., Comin M., Blase X., Jacquemin D. (2021). Reference
Energies for Intramolecular Charge-Transfer Excitations. J. Chem. Theory Comput..

[31] Loos P.-F., Jacquemin D. (2021). A Mountaineering Strategy to Excited States: Highly
Accurate Energies and Benchmarks for Bicyclic Systems. J. Phys. Chem. A.

[32] Roos B. O., Linse P., Siegbahn P. E., Blomberg M. R. (1982). A simple method
for the evaluation of the second-order-perturbation energy from external
double-excitations with a CASSCF reference wavefunction. Chem. Phys..

[33] Li
Manni G., Carlson R. K., Luo S., Ma D., Olsen J., Truhlar D. G., Gagliardi L. (2014). Multiconfiguration
Pair-Density Functional Theory. J. Chem. Theory
Comput..

[34] Carlson R. K., Li Manni G., Sonnenberger A. L., Truhlar D. G., Gagliardi L. (2015). Multiconfiguration
Pair-Density Functional Theory: Barrier Heights and Main Group and
Transition Metal Energetics. J. Chem. Theory
Comput..

[35] Neese F. (2012). The ORCA program
system. WIREs Comput. Mol. Sci..

[36] Neese F. (2018). Software update:
the ORCA program system, version 4.0. WIREs
Comput. Mol. Sci..

[37] Neese F., Wennmohs F., Becker U., Riplinger C. (2020). The ORCA quantum
chemistry program package. J. Chem. Phys..

[38] Hanwell M. D., Curtis D. E., Lonie D. C., Vandermeersch T., Zurek E., Hutchison G. R. (2012). Avogadro: an advanced semantic chemical
editor, visualization, and analysis platform. J. Chem..

[39] Tishchenko O., Zheng J., Truhlar D. G. (2008). Multireference Model
Chemistries
for Thermochemical Kinetics. J. Chem. Theory
Comput..

[40] Wagner L. K., Mitas L. (2007). Energetics and dipole
moment of transition metal monoxides by quantum
Monte Carlo. J. Chem. Phys..

[41] Kozma B., Tajti A., Demoulin B., Izsák R., Nooijen M., Szalay P. G. (2020). A New Benchmark
Set for Excitation
Energy of Charge Transfer States: Systematic Investigation of Coupled
Cluster Type Methods. J. Chem. Theory Comput..

[42] Hohenberg P., Kohn W. (1964). Inhomogeneous Electron Gas. Phys. Rev..

[43] Kohn W., Sham L. J. (1965). Self-Consistent
Equations Including Exchange and Correlation
Effects. Phys. Rev..

[44] Parr, R. G. ; Weitao, Y. Density-Functional Theory of Atoms and Molecules; Oxford University Press, 1995.

[45] Perdew J. P., Schmidt K. (2001). Jacob’s ladder of density functional approximations
for the exchange-correlation energy. AIP Conf.
Proc..

[46] Seidl A., Görling A., Vogl P., Majewski J. A., Levy M. (1996). Generalized
Kohn-Sham schemes and the band-gap problem. Phys. Rev. B.

[47] Bauernschmitt R., Ahlrichs R. (1996). Treatment of electronic
excitations within the adiabatic
approximation of time dependent density functional theory. Chem. Phys. Lett..

[48] Jamorski C., Casida M. E., Salahub D. R. (1996). Dynamic
polarizabilities and excitation
spectra from a molecular implementation of time-dependent density-functional
response theory: N2 as a case study. J. Chem.
Phys..

[49] Li
Manni G. (2023). The OpenMolcas Web: A Community-Driven Approach to
Advancing Computational Chemistry. J. Chem.
Theory Comput..

[50] Roos B. O., Lindh R., Malmqvist P.-Å., Veryazov V., Widmark P.-O. (2004). Main Group
Atoms and Dimers Studied with a New Relativistic ANO Basis Set. J. Phys. Chem. A.

[51] Douglas M., Kroll N. M. (1974). Quantum electrodynamical
corrections to the fine structure
of helium. Ann. Phys..

[52] Hess B. A. (1985). Applicability
of the no-pair equation with free-particle projection operators to
atomic and molecular structure calculations. Phys. Rev. A:At., Mol., Opt. Phys..

[53] Hess B. A. (1986). Relativistic
electronic-structure calculations employing a two-component no-pair
formalism with external-field projection operators. Phys. Rev. A:At., Mol., Opt. Phys..

[54] Berthier G. (1954). Extension
de la methode du champ moleculaire self-consistent a letude des etats
a couches incompletes. C. R. Hebd. Séances
Acad. Sci..

[55] Pople J. A., Nesbet R. K. (1954). Self-Consistent
Orbitals for Radicals. J. Chem. Phys..

[56] Roothaan C. C. J. (1951). New
Developments in Molecular Orbital Theory. Rev.
Mod. Phys..

[57] Becke A. D. (1988). Density-functional
exchange-energy approximation with correct asymptotic behavior. Phys. Rev. A:At., Mol., Opt. Phys..

[58] Lee C., Yang W., Parr R. G. (1988). Development of the Colle-Salvetti
correlation-energy formula into a functional of the electron density. Phys. Rev. B.

[59] Miehlich B., Savin A., Stoll H., Preuss H. (1989). Results obtained with
the correlation energy density functionals of becke and Lee, Yang
and Parr. Chem. Phys. Lett..

[60] Stephens P. J., Devlin F. J., Chabalowski C. F., Frisch M. J. (1994). Ab Initio Calculation
of Vibrational Absorption and Circular Dichroism Spectra Using Density
Functional Force Fields. J. Phys. Chem..

[61] Yanai T., Tew D. P., Handy N. C. (2004). A new hybrid exchange–correlation
functional using the Coulomb-attenuating method (CAM-B3LYP). Chem. Phys. Lett..

[62] Perdew J. P., Burke K., Ernzerhof M. (1996). Generalized
Gradient Approximation
Made Simple. Phys. Rev. Lett..

[63] Perdew J. P., Burke K., Ernzerhof M. (1997). Generalized
Gradient Approximation
Made Simple [Phys. Rev. Lett. Phys. Rev. Lett..

[64] Adamo C., Barone V. (1999). Toward reliable density functional
methods without
adjustable parameters: The PBE0 model. J. Chem.
Phys..

[65] Ernzerhof M., Scuseria G. E. (1999). Assessment of the
Perdew–Burke–Ernzerhof
exchange-correlation functional. J. Chem. Phys..

[66] Weintraub E., Henderson T. M., Scuseria G. E. (2009). Long-Range-Corrected Hybrids Based
on a New Model Exchange Hole. J. Chem. Theory
Comput..

[67] Henderson T. M., Janesko B. G., Scuseria G. E. (2008). Generalized
gradient approximation
model exchange holes for range-separated hybrids. J. Chem. Phys..

[68] Zhao Y., Truhlar D. G. (2006). A new local density
functional for main-group thermochemistry,
transition metal bonding, thermochemical kinetics, and noncovalent
interactions. J. Chem. Phys..

[69] Zhao Y., Truhlar D. G. (2008). The M06 suite of
density functionals for main group
thermochemistry, thermochemical kinetics, noncovalent interactions,
excited states, and transition elements: two new functionals and systematic
testing of four M06-class functionals and 12 other functionals. Theor. Chem. Acc..

[70] Zhao Y., Truhlar D. G. (2006). Density Functional
for Spectroscopy: No Long-Range
Self-Interaction Error, Good Performance for Rydberg and Charge-Transfer
States, and Better Performance on Average than B3LYP for Ground States. J. Phys. Chem. A.

[71] Bursch M., Neugebauer H., Ehlert S., Grimme S. (2022). Dispersion corrected
r2SCAN based global hybrid functionals: r2SCANh, r2SCAN0, and r2SCAN50. J. Chem. Phys..

[72] Widmark P.-O., Malmqvist P.-Å., Roos B. O. (1990). Density matrix averaged atomic natural
orbital (ANO) basis sets for correlated molecular wave functions:
I. First row atoms. Theor. Chim. Acta.

[73] Helmich-Paris B., de Souza B., Neese F., Izsák R. (2021). An improved
chain of spheres for exchange algorithm. J.
Chem. Phys..

[74] Neese F., Wennmohs F., Hansen A., Becker U. (2009). Efficient, approximate
and parallel Hartree–Fock and hybrid DFT calculations. A chain-of-spheres
algorithm for the Hartree–Fock exchange. Chem. Phys..

[75] Stoychev G. L., Auer A. A., Neese F. (2017). Automatic
Generation of Auxiliary
Basis Sets. J. Chem. Theory Comput..

[76] Tange, O. GNU Parallel 20250422 (“Tariffs”), GNU Parallel is a general parallelizer to run multiple serial command line programs in parallel without changing them; Zenodo, 2025.

[77] Pastore M., Angeli C., Cimiraglia R. (2006). The vertical
electronic spectrum
of pyrrole: A second and third order n-electron valence state perturbation
theory study. Chem. Phys. Lett..

[78] Araújo J. P., Lugão I. G., Silva R. P., Martins M. P., Okon I. B., Onate C. A. (2024). A comparative
study of analytical representations of
potential energy curves for CO in its ground electronic state. Int. J. Quantum Chem..

[79] Steimle T. C. (2000). Permanent
electric dipole moments of metal containing molecules. Int. Rev. Phys. Chem..

[80] Huang K. C., Needs R. J., Rajagopal G. (2000). Comment on
Quantum Monte Carlo study
of the dipole moment of CO [J. Chem. Phys. J.
Chem. Phys..

[81] Aquilante F., Lindh R., Bondo Pedersen T. (2007). Unbiased auxiliary
basis sets for
accurate two-electron integral approximations. J. Chem. Phys..

