# Supplementary material for: Automated Active Space Selection with CASCI Dipole Moments
Source: J Chem Theory Comput. 2026 Jul 24;22(15):7624–41. doi: 10.1021/acs.jctc.6c00473 (PMC13472137; doi:10.1021/acs.jctc.6c00473)
Supplement: Supplementary file 1 [file ct6c00473_si_001.pdf]

Supporting Information
Automated Active Space Selection with CASCI Dipole Moments

Benjamin W. Kaufold[†] and Sijia S. Dong[*,†,‡,¶]

†*Department of Chemistry and Chemical Biology, Northeastern University, Boston, Massachussetts 02115, United States*

‡*Department of Chemical Engineering, Northeastern University, Boston, Massachussetts 02115, United States*

¶*Department of Physics, Northeastern University, Boston, Massachussetts 02115, United States*

E-mail: s.dong@northeastern.edu

Contents

1 Excitation Energies for Difluorocarbene Calculated with OpenMolcas and ORCA **3**

2 Cartesian Coordinates of Molecules **3**

3 Additional Datasets **8**

4 Benchmarking Starting Orbitals for CASCI **10**

5 Excited States Chosen for Comparison with CASCI-D^2DM-AS 14

5.1 Molecules with Reference Data for S_5 Excitation Energy 15

5.2 Other Molecules in the NAQ Dataset 21

6 Protocol Performance with Dark States 38

7 Protocol Performance with Conjugated Polar Molecules 40

8 Protocol Performance with Nonpolar Molecules 43

9 Protocol Performance with Transition Metal Monoxides 45

10 Protocol Performance with Bimolecular Complexes 47

11 Ratio of Run Times 50

12 Run Time Data for Other Datasets 51

13 Potential Energy Curve of CO Calculated with Selected Active Spaces 54

1 Excitation Energies for Difluorocarbene Calculated with OpenMolcas and ORCA

CASPT2 excitation energy errors for the difluorocarbene molecule with the (10,13) active space were found to be unphysically high when calculated in OpenMolcas with the ANO-RCC-VTZP basis set. To verify that this issue is software specific, we calculated CASPT2 excitation energy errors with the same active space with ORCA[1–3] 6.1.0 using the ANO-RCC-TZP basis set (for *p*-block elements, this is equivalent to ANO-RCC-VTZP.[4] Excitation energies calculated with each software suite and those given in the QUEST[5] are reported in Table S1. With ORCA, excitation energy errors are far more similar to those reported in QUEST, leading us to conclude that the poor excitation energy errors we found with OpenMolcas were indeed due to a software bug outside of our control rather than a poor choice of active space.

Table S1: Low-lying excitation energies for the difluorocarbene (CF_2) molecule and (10,13) active space as calculated in OpenMolcas and ORCA. Excitation energies provided in the QUEST[5] database are reported for convenience.

**Excitation**	**Excitation Energy (eV)**		
	**OpenMolcas**	**ORCA**	**QUEST**
$\mathbf{S_1}$	28.57	4.71	8.49
$\mathbf{S_2}$	33.38	8.33	8.49
$\mathbf{S_3}$	36.78	9.61	9.92
$\mathbf{S_4}$	38.39	10.48	10.06
$\mathbf{S_5}$	39.45	13.25	10.06

2 Cartesian Coordinates of Molecules

Acetaldehyde

```
C   -0.00124094          0.00000000          0.46104598
C   -0.93054640          0.00000000         -0.71424991
O    1.20624568          0.00000000          0.38083840
H   -0.49162964          0.00000000          1.44957198
```

H	-1.57671241	0.87868017	-0.66257994
H	-1.57671241	-0.87868017	-0.66257994
H	-0.37065389	0.00000000	-1.64609256

Azulene

C	0.00000000	0.00000000	-2.69056265
C	1.14725321	0.00000000	-1.88307845
C	-1.14725321	0.00000000	-1.88307845
C	0.74381474	0.00000000	-0.54178450
C	-0.74381474	0.00000000	-0.54178450
C	0.00000000	0.00000000	2.50243413
C	1.26255146	0.00000000	1.91148961
C	-1.26255146	0.00000000	1.91148961
C	1.58837012	0.00000000	0.55661141
C	-1.58837012	0.00000000	0.55661141
H	0.00000000	0.00000000	-3.76695093
H	2.16780635	0.00000000	-2.22401579
H	-2.16780635	0.00000000	-2.22401579
H	0.00000000	0.00000000	3.58359701
H	2.09826695	0.00000000	2.59551766
H	-2.09826695	0.00000000	2.59551766
H	2.64613562	0.00000000	0.32535383
H	-2.64613562	0.00000000	0.32535383

Benzonitrile

C	0.00000000	0.00000000	-1.97392739
C	0.00000000	0.00000000	-0.54357340
C	1.21054801	0.00000000	0.15130424
C	-1.21054801	0.00000000	0.15130424
C	0.00000000	0.00000000	2.23456149
C	1.20683919	0.00000000	1.53983404
C	-1.20683919	0.00000000	1.53983404
N	0.00000000	0.00000000	-3.13611602
H	2.13750797	0.00000000	-0.39729878
H	-2.13750797	0.00000000	-0.39729878
H	2.14082120	0.00000000	2.07676673
H	-2.14082120	0.00000000	2.07676673
H	0.00000000	0.00000000	3.31208077

Carbon Monoxide

C	0.00000000	0.00000000	-0.66116488
O	0.00000000	0.00000000	0.47237899

Cyclopropene

C	0.00000000	0.00000000	-0.88277808
C	0.00000000	0.64836859	0.47986540
C	0.00000000	-0.64836859	0.47986540
H	0.91153656	0.00000000	-1.47048371
H	-0.91153656	0.00000000	-1.47048371
H	0.00000000	1.57612532	1.01642650
H	0.00000000	-1.57612532	1.01642650

Diazirine

C	0.00000000	0.00000000	-0.14126967
H	0.00000000	0.93077453	-0.68060909
H	0.00000000	-0.93077453	-0.68060909
N	0.61415302	0.00000000	1.20220070
N	-0.61415302	0.00000000	1.20220070

Diazomethane

C	0.00000000	0.00000000	-1.22149978
N	0.00000000	0.00000000	0.07650786
N	0.00000000	0.00000000	1.21670126
H	0.00000000	0.95185857	-1.71597520
H	0.00000000	-0.95185857	-1.71597520

Difluorocarbene

C	0.00000000	0.00000000	-0.60416558
F	0.00000000	1.03089339	0.19110948
F	0.00000000	-1.03089339	0.19110948

Formaldehyde

C	0.00000000	0.00000000	-0.60298508
O	0.00000000	0.00000000	0.60539399
H	0.00000000	0.93467313	-1.18217476
H	0.00000000	-0.93467313	-1.18217476

Formamide

C	-0.05261453	0.00000000	0.67243114
O	0.01051633	0.00000000	-0.65237250
H	0.22637595	0.93521292	1.15609813
H	0.22637595	-0.93521292	1.15609813

Formyl Fluoride

C	0.00283691	0.00000000	0.39858135
O	1.15027151	0.00000000	0.11794170
H	-0.44369578	0.00000000	1.38983618
F	-0.97395764	0.00000000	-0.52586324

Hydrogen Chloride

Cl	0.00000000	0.00000000	-0.01317536
H	0.00000000	0.00000000	1.26199843

Hydrogen Sulfide

S	0.00000000	0.00000000	-0.26652056
H	0.00000000	0.96219289	0.66259489
H	0.00000000	-0.96219289	0.66259489

Imidazole

C	0.22047002	1.09013818	0.00000000
C	-0.80762172	-0.85908302	0.00000000
C	0.55119348	-1.02135132	0.00000000
N	-1.00726640	0.50226832	0.00000000
N	1.18649703	0.20152884	0.00000000
H	0.34661972	2.15771096	0.00000000
H	-1.89181141	0.97423200	0.00000000
H	-1.62120791	-1.55873998	0.00000000
H	1.10425494	-1.94208619	0.00000000

Ketene

C	0.00000000	0.00000000	-1.29547953
C	0.00000000	0.00000000	0.01851350
O	0.00000000	0.00000000	1.18357846
H	0.00000000	0.93893013	-1.81881376
H	0.00000000	-0.93893013	-1.81881376

Methanimine

C	0.05660421	0.00000000	0.58786894
N	0.05696070	0.00000000	-0.68622506
H	-0.84213766	0.00000000	1.20280208
H	1.00795114	0.00000000	1.10806523
H	-0.89936939	0.00000000	-1.03833820

Nitrobenzene

C	0.00000000	0.00000000	-0.17843736
C	1.21534572	0.00000000	0.48757402
C	-1.21534572	0.00000000	0.48757402
C	0.00000000	0.00000000	2.57088310
C	1.20783156	0.00000000	1.87729263
C	-1.20783156	0.00000000	1.87729263
N	0.00000000	0.00000000	-1.64801213
O	-1.08733304	0.00000000	-2.21246453

O	1.08733304	0.00000000	-2.21246453
H	2.12913052	0.00000000	-0.07815867
H	-2.12913052	0.00000000	-0.07815867
H	2.14118233	0.00000000	2.41510435
H	-2.14118233	0.00000000	2.41510435
H	0.00000000	0.00000000	3.64843586

Nitrosyl Hydride

O	0.11165478	0.00000000	1.14017824
N	-0.23694895	0.00000000	-0.01899356
H	0.62529418	0.00000000	-0.62118467

Phosphaethyne

H	0.00000000	0.00000000	-2.13306279
C	0.00000000	0.00000000	-1.06730620
P	0.00000000	0.00000000	0.48367655

Propynal

C	-0.41302871	0.00000000	-0.73502918
C	-0.09458282	0.00000000	0.67642536
C	0.12575216	0.00000000	1.86611591
O	0.42410376	0.00000000	-1.61202369
H	-1.48546959	0.00000000	-0.96717057
H	0.33881211	0.00000000	2.90440602

Pyrrole

C	0.00000000	1.12145687	0.33167012
C	0.00000000	-1.12145687	0.33167012
C	0.00000000	0.71210775	-0.98166028
C	0.00000000	-0.71210775	-0.98166028
N	0.00000000	0.00000000	1.11621673
H	0.00000000	0.00000000	2.11807071
H	0.00000000	2.10426476	0.76640842
H	0.00000000	-2.10426476	0.76640842
H	0.00000000	1.35853844	-1.84067536
H	0.00000000	-1.35853844	-1.84067536

Thiophene

C	0.00000000	1.23479556	-0.05216852
C	0.00000000	-1.23479556	-0.05216852
C	0.00000000	0.71106451	-1.31393498
C	0.00000000	-0.71106451	-1.31393498
S	0.00000000	0.00000000	1.14964115

H	0.00000000	2.27031849	0.23589289
H	0.00000000	-2.27031849	0.23589289
H	0.00000000	1.31638150	-2.20544335
H	0.00000000	-1.31638150	-2.20544335

3 Additional Datasets

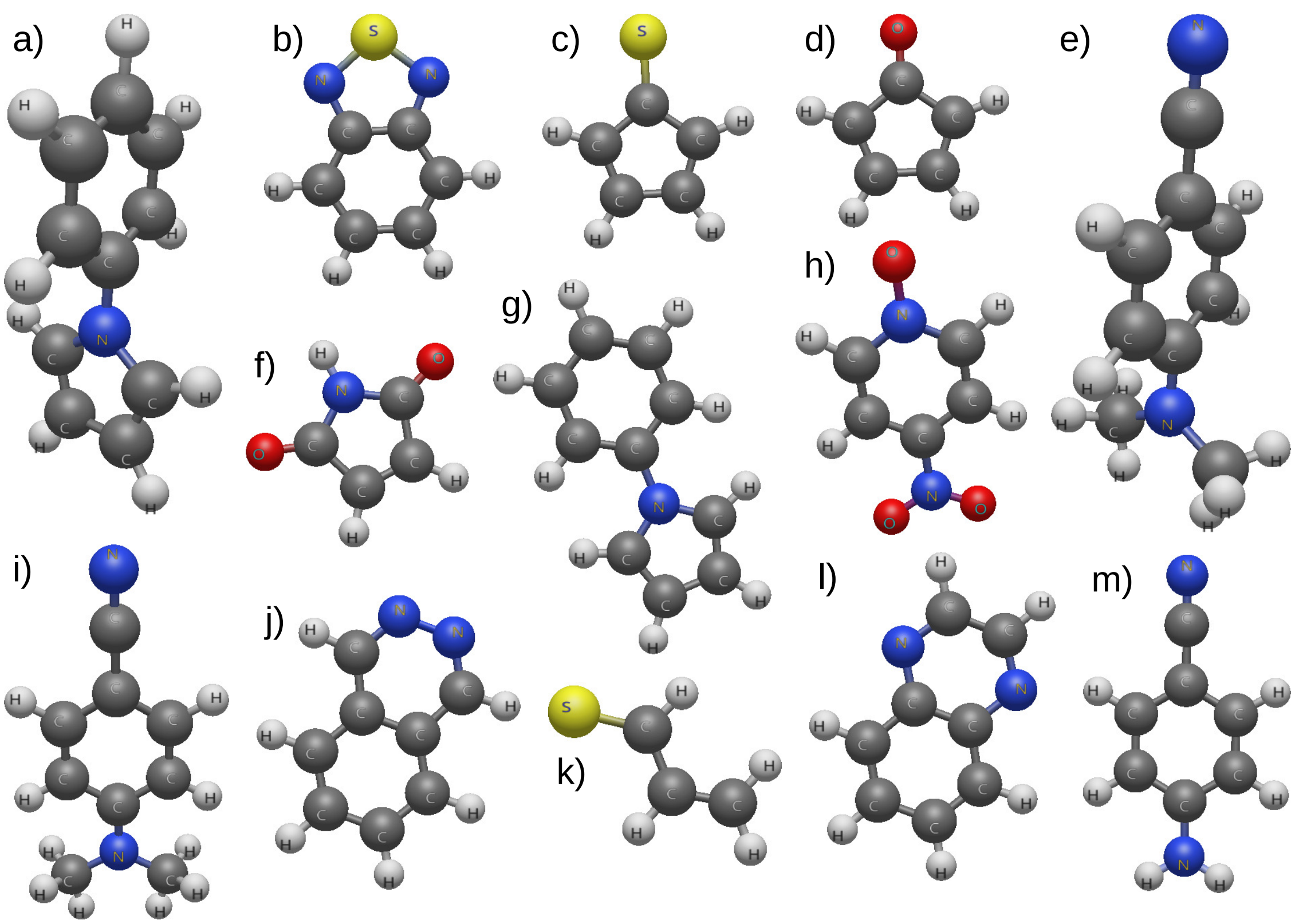

Figure S1: Molecules in the dataset of conjugated polar molecules, with geometries obtained from the website for the QUEST database. Structures are visualized with Avogadro2.[6] a) Twisted *N*-phenylpyrrole; b) benzothiadiazole; c) cyclopentadienethione; d) cyclopentadienone; e) twisted dimethylaminobenzonitrile; f) maleimide; g) *N*-phenylpyrrole; h) nitropyridine *N*-oxide; i) dimethylaminobenzonitrile; j) phthalazine; k) thioacrolein; l) quinoxaline; m) aminobenzonitrile

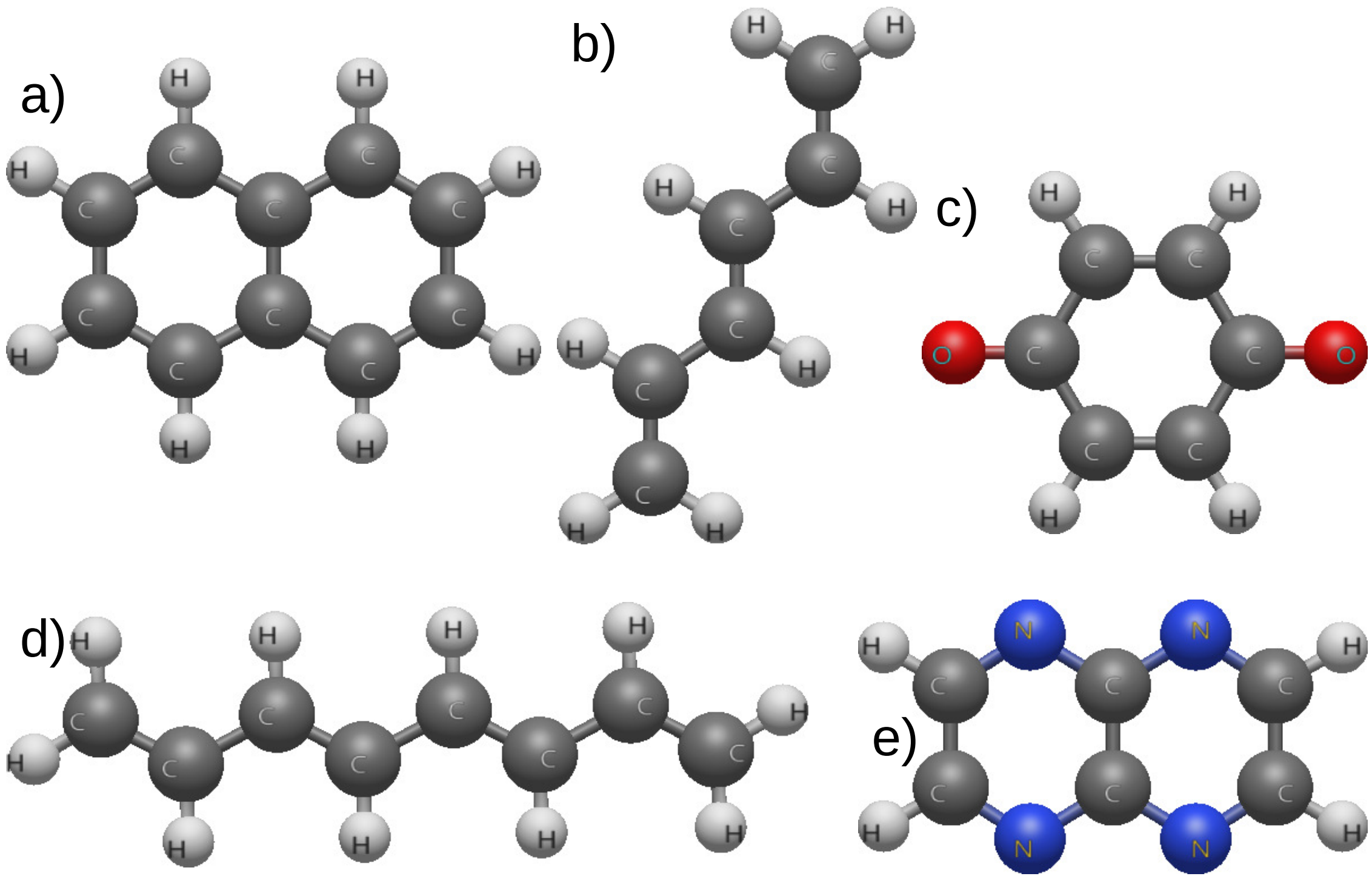

Figure S2: Molecules in the dataset of nonpolar molecules, with geometries obtained from the website for the QUEST database. Structures are visualized with Avogadro2.[6] a) Naphthalene; b) hexatriene; c) benzoquinine; d) octatetraene; e) aza-naphthalene.

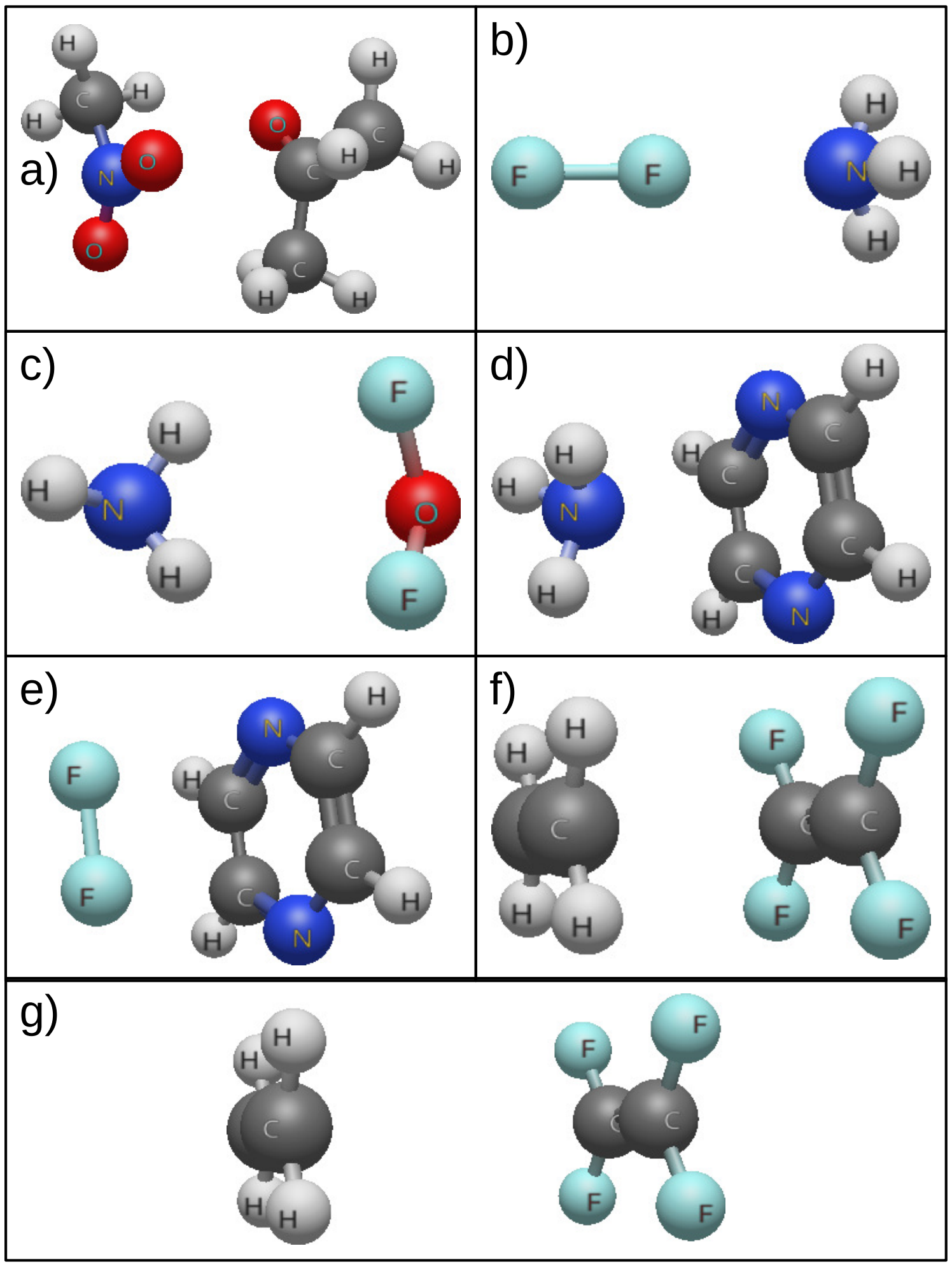

Figure S3: Molecules in the dataset of bimolecular complexes, with geometries obtained from the work of Kozma et al.[7] Structures are visualized with Avogadro2.[6] a) Acetone-nitromethane; b) ammonia-fluorine; c) ammonia-oxygen difluoride; d) ammonia-pyrazine; e) pyrazine-fluorine; f) tetrafluoroethylene-ethylene, 3.5 Å spacing; g) tetrafluoroethylene-ethylene, 5 Å spacing.

4 Benchmarking Starting Orbitals for CASCI

In order to establish the best starting orbitals to provide to CASCI for calculating the dipole moment, we compared dipole moments calculated with CASCI using various starting orbitals to reliable references. We accomplished this as follows:

1. Assemble a set of molecules with closed-shell, singlet, neutral ground states from the QUEST database[5] with nonzero dipole moments. This is shown in Figure S4.

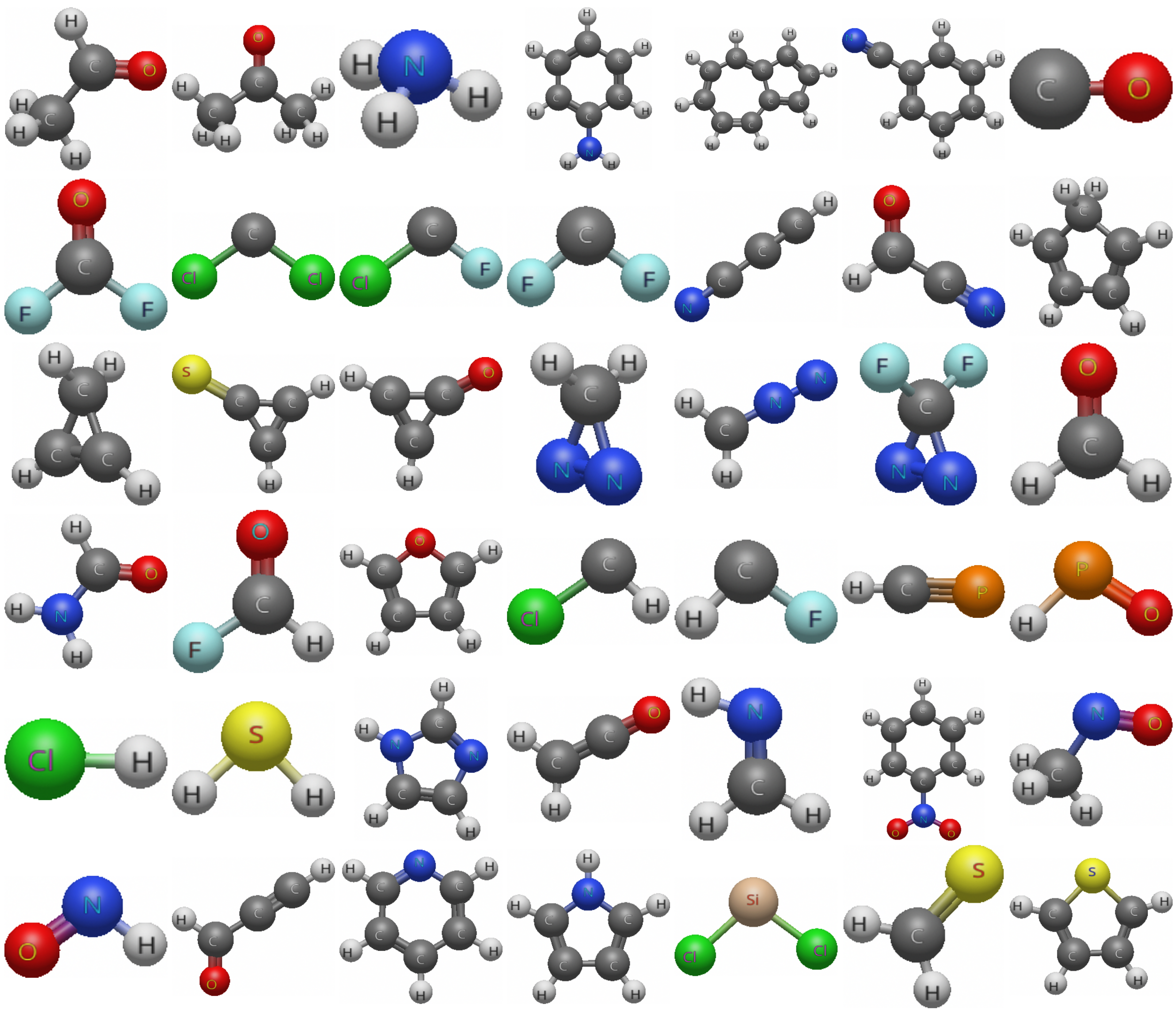

Figure S4: Dataset of QUEST molecules used for benchmarking starting orbitals for calculating dipole moments with CASCI.

2. For all of these molecules, carry out CASCI calculations...

   - ...where CASCI is preceded by restricted Hartree-Fock (RHF), unrestricted Hartree-Fock (UHF), restricted density functional theory with LSDA,[8] BLYP, B3LYP, PBE, PBE0, M06-L, M06-2X, or M06-HF density functionals, or the second-order Møller-Plesset perturbation theory (MP2), which produce the starting orbitals.
   - ...with all active spaces $(n_\mathrm{e}, n_\mathrm{o})$ such that $\frac{n_\mathrm{e}}{2} \in \mathbb{Z},\ 4 \leq n_\mathrm{e} \leq 14,\ n_\mathrm{o} \in \mathbb{Z},\ \frac{n_\mathrm{e}}{2} \leq n_\mathrm{o} \leq 14$.

3. Run calculations using the coupled cluster[9] with singles, doubles[10–13] and perturbative triples[14] (CCSD(T)) method and the *aug*-cc-pVTZ-DK[15–17] basis set to obtain reasonable accurate reference dipole moments for comparison. This is in addition to the reference dipole moments available in NIST.

4. For only those molecules in the dataset for which reference dipole moments were available in the NIST database, take absolute differences in total S_0 dipole moment value between CASCI with each type of starting orbital and NIST, averaged over all molecules and active spaces, excluding calculations which did not complete.

5. For only those molecules in the dataset for which CCSD(T)/*aug*-cc-pVTZ-DK dipole moments could be calculated, take absolute differences in total S_0 dipole moment value between CASCI with each type of starting orbital and CCSD(T)/*aug*-cc-pVTZ-DK, averaged over all molecules and active spaces, excluding calculations which did not complete.

Average dipole moment differences between CASCI for each type of starting orbital and the reference are shown in Figure S5.

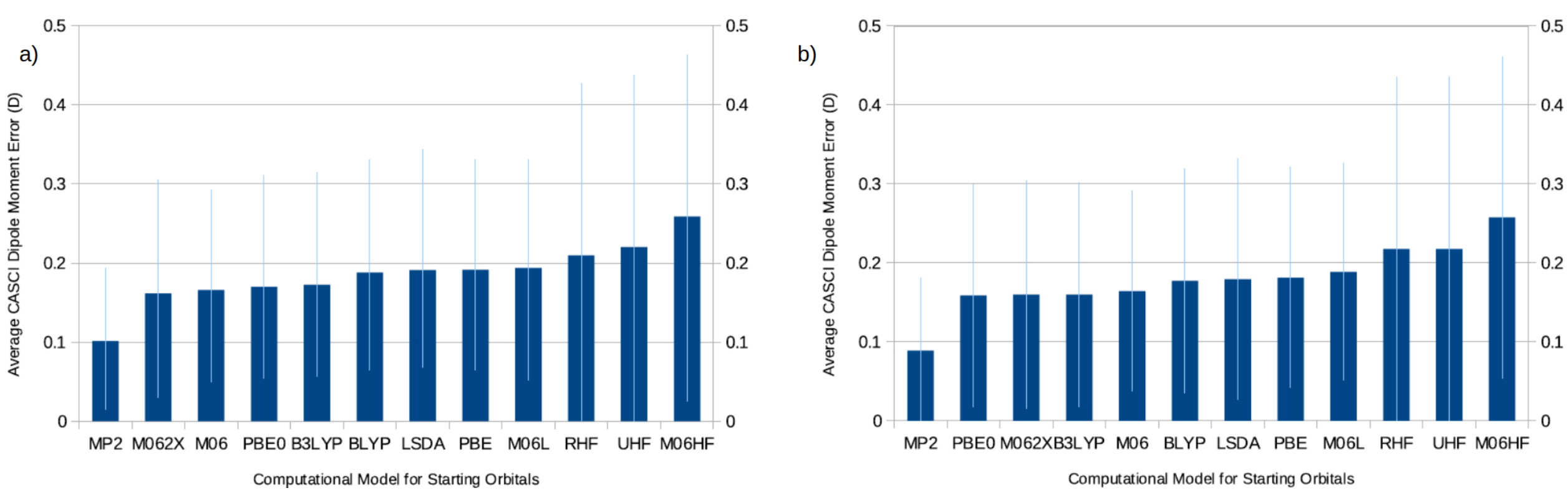

Figure S5: Average dipole moment differences between CASCI and a) NIST or b) CCSD(T)/*aug*-cc-pVTZ-DK for all molecules and active spaces in the test dataset with completed calculations. Thin vertical lines represent the standard deviation. Sorted in ascending order.

Additionally, we take average *relative* dipole moment differences between CASCI and

NIST or CCSD(T)/*aug*-cc-pVTZ-DK, where the difference is divided by the value of the reference dipole moment before averaging. These are shown in Figure S6.

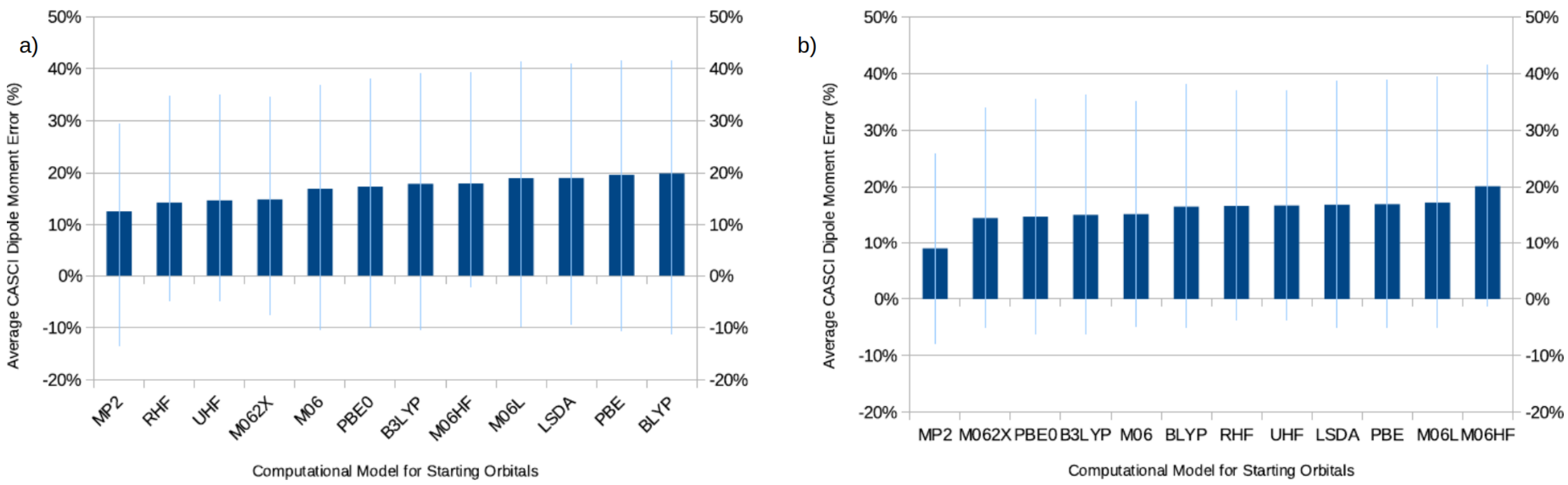

Figure S6: Average relative dipole moment differences between CASCI and a) NIST or b) CCSD(T)/*aug*-cc-pVTZ-DK for all molecules and active spaces in the test dataset with completed calculations. Thin vertical lines represent the standard deviation. Sorted in ascending order.

CASCI with MP2 starting orbitals always produces the most accurate dipole moments (smallest average difference from the reference) regardless of whether dipole moments are compared to NIST or CCSD(T)/*aug*-cc-pVTZ-DK, or whether absolute or relative dipole moment errors are averaged. As such, we take MP2 to be the best method for producing starting orbitals for calculating dipole moments with CASCI, and precede all CASCI dipole moment calculations discussed in the main text with MP2. We recognize that MP2 is more computationally expensive than other methods we have tested for generating starting orbitals for CASCI. Regardless, in the main text section “Comparison of Run Time” and Figure 11, we show that even when preceded by an MP2 calculation, CASCI is still substantially more efficient than CASSCF.

5 Excited States Chosen for Comparison with CASCI-D^2DM-AS

Table S2: Likelihood for each CASCI excited state to be chosen for comparison with reference dipole moments in CASCI-D^2DM-AS, for all molecules in NAQ and all active spaces (not only the ones eventually selected).

**Excited State**	**Probability of Choice (%)**
S_1	23.23%
S_2	22.48%
S_3	11.80%
S_4	17.89%
S_5	24.60%

5.1 Molecules with Reference Data for S_5 Excitation Energy

Table S3: Azulene CASCI excited states used in the CASCI-D^2DM-AS protocol for comparison with reference dipole moments, for each active space tested. Active spaces chosen with reference dipole moments from at least one density functional are shown in a box, along with the corresponding state. In QUEST6,[18] S_2 and S_3 are considered charge transfer states.

**Active Space**	**CASCI State (S_n)**	**Active Space**	**CASCI State (S_n)**
(6,6)	3	(10,8)	5
(6,7)	2	(10,9)	4
(6,8)	2	(10,10)	5
(6,9)	2	(10,11)	5
(6,10)	2	(10,12)	5
(6,11)	2	(10,13)	5
(6,12)	2	(10,14)	5
(6,13)	2	(12,9)	5
[(6,14)]	[2]	(12,10)	4
(8,7)	3	(12,11)	5
[(8,8)]	[2]	(12,12)	5
(8,9)	3	(12,13)	5
(8,10)	3	(12,14)	5
(8,11)	3	(14,10)	5
(8,12)	3	(14,11)	4
(8,13)	3	(14,12)	5
(8,14)	3	(14,13)	5
		[(14,14)]	[5]

Table S4: Benzonitrile CASCI excited states used in the CASCI-D^{2}DM-AS protocol for comparison with reference dipole moments, for each active space tested. Active spaces chosen with reference dipole moments from at least one density functional are shown in a box, along with the corresponding state. In QUEST6,[18] S_5 is considered the charge transfer state.

**Active Space**	**CASCI State (S_n)**	**Active Space**	**CASCI State (S_n)**
(6,6)	3	(10,8)	4
[(6,7)]	[2]	(10,9)	3
(6,8)	2	(10,10)	5
(6,9)	2	(10,11)	5
(6,10)	2	(10,12)	5
(6,11)	2	[(10,13)]	[5]
(6,12)	2	(10,14)	5
(6,13)	3	(12,9)	4
(6,14)	3	(12,10)	[3]
(8,7)	5	(12,11)	4
(8,8)	3	[(12,12)]	[4]
[(8,9)]	[3]	(12,13)	4
(8,10)	3	(12,14)	4
(8,11)	3	[(14,10)]	[5]
(8,12)	3	[(14,11)]	[3]
(8,13)	3	(14,12)	4
[(8,14)]	[3]	(14,13)	4
		(14,14)	4

Table S5: Carbon monoxide CASCI excited states used in the CASCI-D^{2}DM-AS protocol for comparison with reference dipole moments, for each active space tested. Active spaces chosen with reference dipole moments from at least one density functional are shown in a box, along with the corresponding state.

**Active Space**	**CASCI State (S_n)**	**Active Space**	**CASCI State (S_n)**
(6,6)	1	(10,8)	3
(6,7)	1	(10,9)	3
(6,8)	1	(10,10)	3
(6,9)	1	(10,11)	3
(6,10)	1	(10,12)	1
(6,11)	1	(10,13)	1
(6,12)	1	(10,14)	1
(6,13)	1	(12,9)	3
$\boxed{(6,14)}$	$\boxed{1}$	(12,10)	3
(8,7)	1	(12,11)	3
(8,8)	1	(12,12)	3
(8,9)	1	(12,13)	1
(8,10)	1	(12,14)	1
(8,11)	2	(14,10)	3
(8,12)	2	(14,11)	3
(8,13)	2	(14,12)	3
$\boxed{(8,14)}$	$\boxed{1}$	(14,13)	3
		(14,14)	1

Table S6: Formaldehyde CASCI excited states used in the CASCI-D^2DM-AS protocol for comparison with reference dipole moments, for each active space tested. Active spaces chosen with reference dipole moments from at least one density functional are shown in a box, along with the corresponding state.

**Active Space**	**CASCI State (S_n)**	**Active Space**	**CASCI State (S_n)**
(6,6)	4	(10,8)	2
(6,7)	5	(10,9)	2
(6,8)	5	(10,10)	2
(6,9)	5	[(10,11)]	[5]
(6,10)	5	(10,12)	2
(6,11)	5	(10,13)	2
(6,12)	5	[(10,14)]	[5]
(6,13)	5	(12,9)	3
(6,14)	5	(12,10)	2
[(8,7)]	[2]	(12,11)	2
(8,8)	2	(12,12)	5
(8,9)	2	(12,13)	5
(8,10)	5	(12,14)	5
(8,11)	5	(14,10)	3
(8,12)	2	(14,11)	2
(8,13)	5	(14,12)	2
(8,14)	5	[(14,13)]	[5]
		(14,14)	5

Table S7: Pyrrole CASCI excited states used in the CASCI-D^2DM-AS protocol for comparison with reference dipole moments, for each active space tested. Active spaces chosen with reference dipole moments from at least one density functional are shown in a box, along with the corresponding state.

**Active Space**	**CASCI State (S_n)**	**Active Space**	**CASCI State (S_n)**
(6,6)	5	(10,8)	5
$\boxed{(6,7)}$	$\boxed{5}$	(10,9)	5
(6,8)	5	(10,10)	5
(6,9)	5	(10,11)	5
(6,10)	5	(10,12)	5
(6,11)	5	(10,13)	5
(6,12)	3	(10,14)	1
(6,13)	3	(12,9)	5
(6,14)	5	(12,10)	5
(8,7)	5	(12,11)	5
(8,8)	5	(12,12)	5
(8,9)	5	(12,13)	5
(8,10)	5	(12,14)	5
(8,11)	5	(14,10)	5
(8,12)	5	(14,11)	5
(8,13)	5	(14,12)	5
(8,14)	5	(14,13)	5
		(14,14)	5

Table S8: Thiophene CASCI excited states used in the CASCI-D^{2}DM-AS protocol for comparison with reference dipole moments, for each active space tested. Active spaces chosen with reference dipole moments from at least one density functional are shown in a box, along with the corresponding state.

**Active Space**	**CASCI State (S_n)**	**Active Space**	**CASCI State (S_n)**
(6,6)	4	(10,8)	3
(6,7)	4	(10,9)	3
(6,8)	4	(10,10)	3
(6,9)	4	(10,11)	3
(6,10)	4	(10,12)	3
(6,11)	4	(10,13)	3
(6,12)	4	(10,14)	3
(6,13)	4	(12,9)	3
(6,14)	4	(12,10)	3
(8,7)	4	(12,11)	3
(8,8)	4	(12,12)	3
(8,9)	4	(12,13)	3
(8,10)	4	(12,14)	3
(8,11)	4	(14,10)	3
(8,12)	4	(14,11)	3
(8,13)	4	(14,12)	3
(8,14)	4	(14,13)	3
		(14,14)	3

5.2 Other Molecules in the NAQ Dataset

Table S9: Acetaldehyde CASCI excited states used in the CASCI-D^{2}DM-AS protocol for comparison with reference dipole moments, for each active space tested.

**Active Space**	**CASCI State ($\mathbf{S_n}$)**	**Active Space**	**CASCI State ($\mathbf{S_n}$)**
(6,6)	1	(10,8)	5
(6,7)	4	(10,9)	5
(6,8)	5	(10,10)	5
(6,9)	1	(10,11)	5
(6,10)	1	(10,12)	5
(6,11)	1	(10,13)	5
(6,12)	3	(10,14)	5
(6,13)	3	(12,8)	5
(6,14)	3	(12,10)	4
(8,7)	4	(12,11)	4
(8,8)	5	(12,12)	5
(8,9)	5	(12,13)	5
(8,10)	5	(12,14)	5
(8,11)	5	(14,10)	5
(8,12)	4	(14,11)	5
(8,13)	4	(14,12)	5
(8,14)	4	(14,13)	5
		(14,14)	5

Table S10: Aniline CASCI excited states used in the CASCI-D^2DM-AS protocol for comparison with reference dipole moments, for each active space tested. Active spaces chosen with reference dipole moments from at least one density functional are shown in a box, along with the corresponding state. In QUEST6,[18] S_3 is considered the charge transfer state.

**Active Space**	**CASCI State (S_n)**	**Active Space**	**CASCI State (S_n)**
(6,6)	2	$\boxed{(10,8)}$	$\boxed{2}$
(6,7)	2	(10,9)	2
(6,8)	2	(10,10)	2
(6,9)	2	(10,11)	2
(6,10)	2	(10,12)	2
(6,11)	2	(10,13)	2
(6,12)	2	(10,14)	2
(6,13)	2	(12,9)	2
(6,14)	2	(12,10)	2
(8,7)	2	(12,11)	2
(8,8)	2	(12,12)	2
(8,9)	2	(12,13)	2
(8,10)	2	(12,14)	2
(8,11)	2	(14,10)	2
(8,12)	2	(14,11)	2
(8,13)	2	(14,12)	2
(8,14)	2	(14,13)	2
		(14,14)	2

Table S11: Cyclopropene CASCI excited states used in the CASCI-D^2DM-AS protocol for comparison with reference dipole moments, for each active space tested.

**Active Space**	**CASCI State (S_n)**	**Active Space**	**CASCI State (S_n)**
(6,6)	4	(10,8)	5
(6,7)	4	(10,9)	5
(6,8)	4	(10,10)	5
(6,9)	4	(10,11)	5
(6,10)	4	(10,12)	5
(6,11)	4	(10,13)	5
(6,12)	4	(10,14)	5
(6,13)	4	(12,9)	5
(6,14)	4	(12,10)	5
(8,7)	4	(12,11)	5
(8,8)	4	(12,12)	5
(8,9)	4	(12,13)	5
(8,10)	4	(12,14)	5
(8,11)	4	(14,10)	5
(8,12)	4	(14,11)	5
(8,13)	4	(14,12)	5
(8,14)	4	(14,13)	5
		(14,14)	5

Table S12: Diazirine CASCI excited states used in the CASCI-D^{2}DM-AS protocol for comparison with reference dipole moments, for each active space tested.

**Active Space**	**CASCI State (S_n)**	**Active Space**	**CASCI State (S_n)**
(6,6)	1	(10,8)	3
(6,7)	4	(10,9)	2
(6,8)	4	(10,10)	2
(6,9)	5	(10,11)	2
(6,10)	5	(10,12)	2
(6,11)	5	(10,13)	2
(6,12)	5	(10,14)	2
(6,13)	5	(12,9)	3
(6,14)	5	(12,10)	3
(8,7)	1	(12,11)	3
(8,8)	1	(12,12)	2
(8,9)	1	(12,13)	2
(8,10)	1	(12,14)	3
(8,11)	1	(14,10)	3
(8,12)	1	(14,11)	3
(8,13)	1	(14,12)	3
(8,14)	1	(14,13)	3
		(14,14)	3

Table S13: Diazomethane CASCI excited states used in the CASCI-D^{2}DM-AS protocol for comparison with reference dipole moments, for each active space tested.

**Active Space**	**CASCI State (S_n)**	**Active Space**	**CASCI State (S_n)**
(6,6)	1	(10,8)	1
(6,7)	1	(10,9)	1
(6,8)	1	(10,10)	1
(6,9)	1	(10,11)	1
(6,10)	1	(10,12)	1
(6,11)	1	(10,13)	1
(6,12)	1	(10,14)	1
(6,13)	1	(12,9)	1
(6,14)	1	(12,10)	1
(8,7)	1	(12,11)	1
(8,8)	1	(12,12)	1
(8,9)	1	(12,13)	1
(8,10)	1	(12,14)	1
(8,11)	1	(14,10)	1
(8,12)	1	(14,11)	1
(8,13)	1	(14,12)	1
(8,14)	1	(14,13)	1
		(14,14)	1

Table S14: Formamide CASCI excited states used in the CASCI-D^2DM-AS protocol for comparison with reference dipole moments, for each active space tested.

**Active Space**	**CASCI State (S_n)**	**Active Space**	**CASCI State (S_n)**
(6,6)	3	(10,8)	5
(6,7)	5	(10,9)	2
(6,8)	5	(10,10)	2
(6,9)	4	(10,11)	2
(6,10)	4	(10,12)	2
(6,11)	5	(10,13)	2
(6,12)	5	(10,14)	2
(6,13)	4	(12,9)	5
(6,14)	4	(12,10)	2
(8,7)	2	(12,11)	2
(8,8)	2	(12,12)	1
(8,9)	2	(12,13)	2
(8,10)	2	(12,14)	2
(8,11)	2	(14,10)	2
(8,12)	2	(14,11)	2
(8,13)	2	(14,12)	2
(8,14)	2	(14,13)	1
		(14,14)	2

Table S15: Formyl fluoride CASCI excited states used in the CASCI-D^{2}DM-AS protocol for comparison with reference dipole moments, for each active space tested.

**Active Space**	**CASCI State (S_n)**	**Active Space**	**CASCI State (S_n)**
(6,6)	5	(10,8)	5
(6,7)	4	(10,9)	5
(6,8)	4	(10,10)	5
(6,9)	4	(10,11)	5
(6,10)	4	(10,12)	5
(6,11)	4	(10,13)	4
(6,12)	5	(10,14)	4
(6,13)	5	(12,9)	5
(6,14)	5	(12,10)	5
(8,7)	4	(12,11)	5
(8,8)	4	(12,12)	5
(8,9)	4	(12,13)	5
(8,10)	4	(12,14)	5
(8,11)	4	(14,10)	2
(8,12)	4	(14,11)	3
(8,13)	4	(14,12)	5
(8,14)	4	(14,13)	5
		(14,14)	5

Table S16: Hydrogen cyaphide CASCI excited states used in the CASCI-D^{2}DM-AS protocol for comparison with reference dipole moments, for each active space tested.

**Active Space**	**CASCI State (S_n)**	**Active Space**	**CASCI State (S_n)**
(6,6)	2	(10,8)	2
(6,7)	2	(10,9)	1
(6,8)	3	(10,10)	1
(6,9)	2	(10,11)	1
(6,10)	3	(10,12)	3
(6,11)	2	(10,13)	2
(6,12)	2	(10,14)	2
(6,13)	2	(12,9)	2
(6,14)	1	(12,10)	1
(8,7)	2	(12,11)	1
(8,8)	2	(12,12)	1
(8,9)	3	(12,13)	3
(8,10)	2	(12,14)	2
(8,11)	3	(14,10)	2
(8,12)	2	(14,11)	1
(8,13)	2	(14,12)	1
(8,14)	2	(14,13)	1
		(14,14)	3

Table S17: Hydrogen chloride CASCI excited states used in the CASCI-D^{2}DM-AS protocol for comparison with reference dipole moments, for each active space tested. Active spaces chosen with reference dipole moments from at least one density functional are shown in a box, along with the corresponding state. In QUEST6, S_1 and S_2 are considered charge transfer states.

**Active Space**	**CASCI State (S_n)**	**Active Space**	**CASCI State (S_n)**
(6,6)	1	(10,8)	1
(6,7)	1	(10,9)	1
(6,8)	1	(10,10)	1
(6,9)	1	(10,11)	1
(6,10)	2	(10,12)	2
(6,11)	1	(10,13)	1
(6,12)	1	(10,14)	1
(6,13)	1	(12,9)	1
(6,14)	1	(12,10)	1
(8,7)	1	(12,11)	1
(8,8)	1	(12,12)	1
(8,9)	1	(12,13)	2
(8,10)	1	(12,14)	1
(8,11)	2	(14,10)	1
(8,12)	1	(14,11)	1
(8,13)	1	[(14,12)]	[1]
(8,14)	1	(14,13)	1
		(14,14)	2

Table S18: Hydrogen sulfide CASCI excited states used in the CASCI-D^{2}DM-AS protocol for comparison with reference dipole moments, for each active space tested.

**Active Space**	**CASCI State (S_n)**	**Active Space**	**CASCI State (S_n)**
(6,6)	5	(10,8)	5
(6,7)	5	(10,9)	5
(6,8)	5	(10,10)	5
(6,9)	2	(10,11)	2
(6,10)	2	(10,12)	2
(6,11)	2	(10,13)	2
(6,12)	2	(10,14)	2
(6,13)	2	(12,9)	5
(6,14)	2	(12,10)	5
(8,7)	5	(12,11)	5
(8,8)	5	(12,12)	2
(8,9)	5	(12,13)	2
(8,10)	2	(12,14)	2
(8,11)	2	(14,10)	5
(8,12)	2	(14,11)	5
(8,13)	2	(14,12)	5
(8,14)	2	(14,13)	2
		(14,14)	2

Table S19: Imidazole CASCI excited states used in the CASCI-D^2DM-AS protocol for comparison with reference dipole moments, for each active space tested.

**Active Space**	**CASCI State (S_n)**	**Active Space**	**CASCI State (S_n)**
(6,6)	4	(10,8)	2
(6,7)	4	(10,9)	4
(6,8)	4	(10,10)	4
(6,9)	4	(10,11)	4
(6,10)	4	(10,12)	4
(6,11)	4	(10,13)	4
(6,12)	4	(10,14)	4
(6,13)	4	(12,9)	2
(6,14)	4	(12,10)	3
(8,7)	4	(12,11)	3
(8,8)	4	(12,12)	4
(8,9)	4	(12,13)	4
(8,10)	4	(12,14)	4
(8,11)	4	(14,10)	3
(8,12)	5	(14,11)	3
(8,13)	5	(14,12)	3
(8,14)	5	(14,13)	3
		(14,14)	4

Table S20: Ketene CASCI excited states used in the CASCI-D^{2}DM-AS protocol for comparison with reference dipole moments, for each active space tested.

**Active Space**	**CASCI State (S_n)**	**Active Space**	**CASCI State (S_n)**
(6,6)	1	(10,8)	1
(6,7)	1	(10,9)	1
(6,8)	1	(10,10)	1
(6,9)	1	(10,11)	1
(6,10)	1	(10,12)	1
(6,11)	1	(10,13)	1
(6,12)	1	(10,14)	1
(6,13)	1	(12,9)	1
(6,14)	1	(12,10)	1
(8,7)	1	(12,11)	1
(8,8)	1	(12,12)	1
(8,9)	1	(12,13)	1
(8,10)	1	(12,14)	1
(8,11)	1	(14,10)	1
(8,12)	1	(14,11)	1
(8,13)	1	(14,12)	1
(8,14)	1	(14,13)	1
		(14,14)	1

Table S21: Methanimine CASCI excited states used in the CASCI-D^{2}DM-AS protocol for comparison with reference dipole moments, for each active space tested.

**Active Space**	**CASCI State (S_n)**	**Active Space**	**CASCI State (S_n)**
(6,6)	4	(10,8)	5
(6,7)	4	(10,9)	1
(6,8)	5	(10,10)	5
(6,9)	4	(10,11)	5
(6,10)	5	(10,12)	5
(6,11)	4	(10,13)	5
(6,12)	4	(10,14)	5
(6,13)	4	(12,9)	4
(6,14)	5	(12,10)	1
(8,7)	1	(12,11)	1
(8,8)	5	(12,12)	1
(8,9)	1	(12,13)	1
(8,10)	5	(12,14)	1
(8,11)	5	(14,10)	4
(8,12)	5	(14,11)	1
(8,13)	5	(14,12)	1
(8,14)	5	(14,13)	1
		(14,14)	1

Table S22: Nitrobenzene CASCI excited states used in the CASCI-D^{2}DM-AS protocol for comparison with reference dipole moments, for each active space tested. Active spaces chosen with reference dipole moments from at least one density functional are shown in a box, along with the corresponding state. In QUEST6, S_4 is considered the charge transfer state.

**Active Space**	**CASCI State (S_n)**	**Active Space**	**CASCI State (S_n)**
(6,6)	4	(10,8)	4
(6,7)	4	(10,9)	4
(6,8)	4	(10,10)	4
(6,9)	4	(10,11)	4
(6,10)	4	$\boxed{(10,12)}$	$\boxed{2}$
(6,11)	4	(10,13)	2
(6,12)	4	(10,14)	2
(6,13)	4	(12,9)	4
(6,14)	4	(12,10)	4
(8,7)	4	(12,11)	4
(8,8)	4	(12,12)	4
(8,9)	4	(12,13)	2
(8,10)	4	(12,14)	2
(8,11)	2	(14,10)	4
(8,12)	2	(14,11)	4
(8,13)	2	(14,12)	4
(8,14)	2	(14,13)	4
		(14,14)	2

Table S23: Nitrosomethane CASCI excited states used in the CASCI-D^{2}DM-AS protocol for comparison with reference dipole moments, for each active space tested.

**Active Space**	**CASCI State (S_n)**	**Active Space**	**CASCI State (S_n)**
(6,6)	5	(10,8)	3
(6,7)	5	(10,9)	3
(6,8)	5	(10,10)	3
(6,9)	4	(10,11)	3
(6,10)	4	(10,12)	3
(6,11)	5	(10,13)	3
(6,12)	5	(10,14)	3
(6,13)	5	(12,9)	4
(6,14)	5	(12,10)	4
(8,7)	2	(12,11)	4
(8,8)	2	(12,12)	2
(8,9)	2	(12,13)	2
(8,10)	2	(12,14)	2
(8,11)	2	(14,10)	5
(8,12)	2	(14,11)	5
(8,13)	2	(14,12)	4
(8,14)	2	(14,13)	5
		(14,14)	4

Table S24: Nitrosyl hydride CASCI excited states used in the CASCI-D^{2}DM-AS protocol for comparison with reference dipole moments, for each active space tested.

**Active Space**	**CASCI State (S_n)**	**Active Space**	**CASCI State (S_n)**
(6,6)	2	(10,8)	2
(6,7)	3	(10,9)	5
(6,8)	3	(10,10)	5
(6,9)	3	(10,11)	5
(6,10)	3	(10,12)	5
(6,11)	3	(10,13)	5
(6,12)	2	(10,14)	5
(6,13)	2	(12,9)	2
(6,14)	2	(12,10)	2
(8,7)	2	(12,11)	2
(8,8)	4	(12,12)	2
(8,9)	4	(12,13)	2
(8,10)	4	(12,14)	2
(8,11)	4	(14,10)	2
(8,12)	4	(14,11)	2
(8,13)	5	(14,12)	2
(8,14)	5	(14,13)	2
		(14,14)	2

Table S25: Propynal CASCI excited states used in the CASCI-D^{2}DM-AS protocol for comparison with reference dipole moments, for each active space tested.

**Active Space**	**CASCI State (S_n)**	**Active Space**	**CASCI State (S_n)**
(6,6)	1	(10,8)	1
(6,7)	1	(10,9)	1
(6,8)	1	(10,10)	1
(6,9)	1	(10,11)	1
(6,10)	1	(10,12)	1
(6,11)	1	(10,13)	1
(6,12)	1	(10,14)	1
(6,13)	1	(12,9)	2
(6,14)	1	(12,10)	5
(8,7)	1	(12,11)	5
(8,8)	1	(12,12)	5
(8,9)	1	(12,13)	5
(8,10)	1	(12,14)	5
(8,11)	1	(14,10)	1
(8,12)	1	(14,11)	1
(8,13)	1	(14,12)	1
(8,14)	1	(14,13)	1
		(14,14)	1

6 Protocol Performance with Dark States

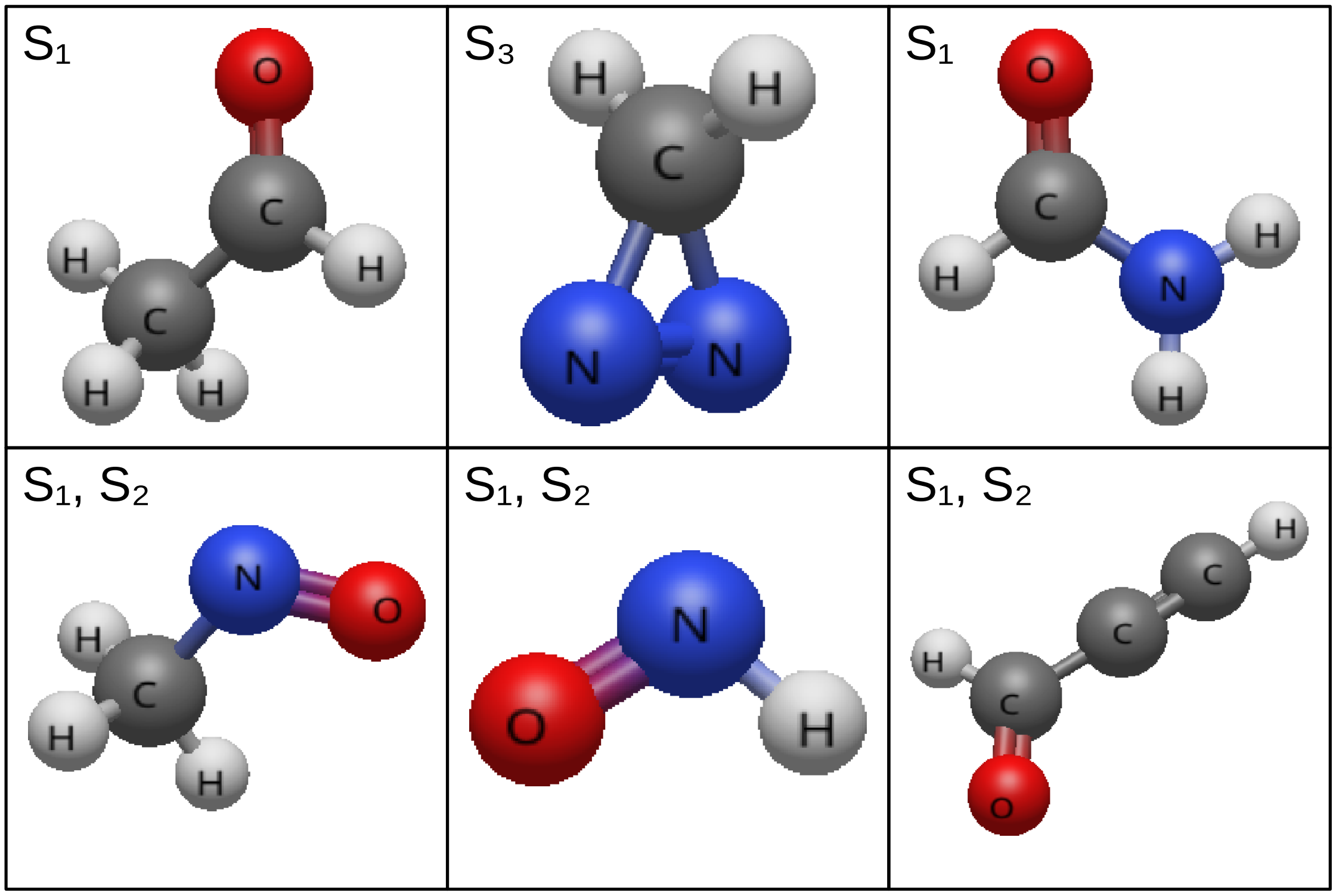

Figure S7: Molecules in the NAQ dataset with excitations listed as having oscillator strengths of 0.000 in QUEST. For each molecule, the excitations included for analysis are indicated in the top left. Structures are visualized with Avogadro2.[6]

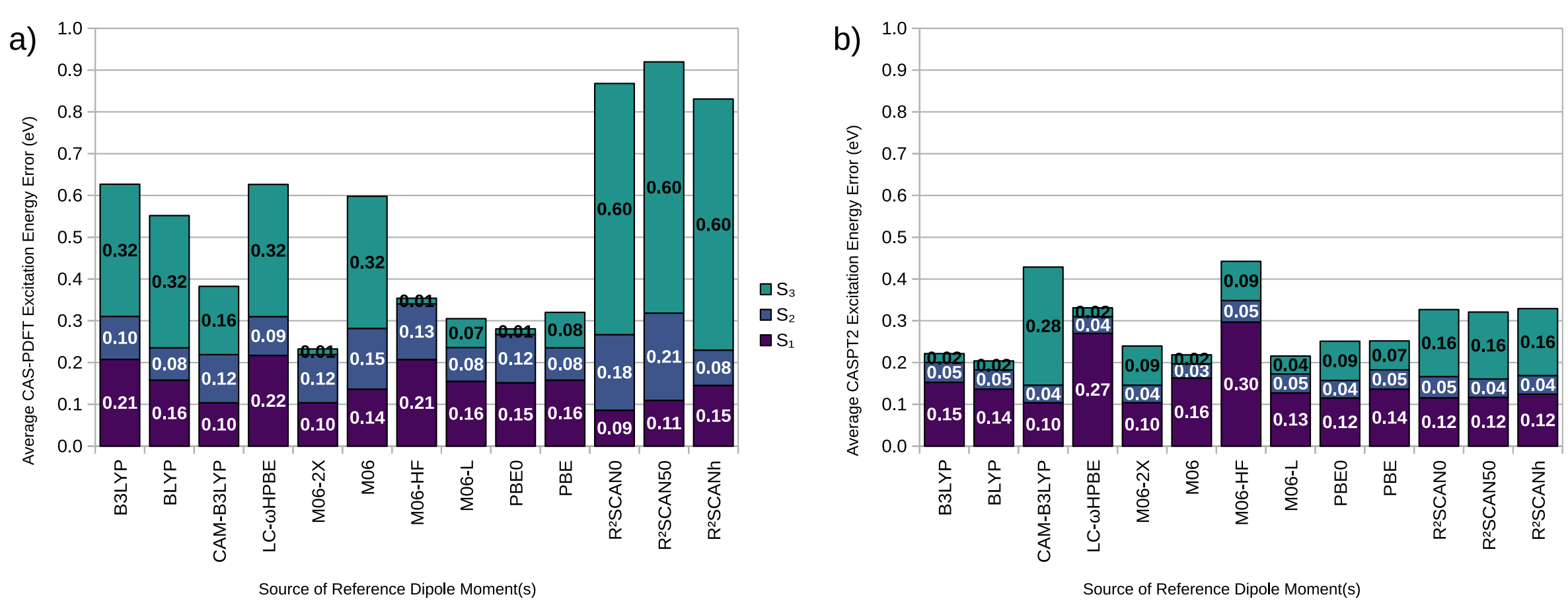

Figure S8: Low-lying a) CAS-PDFT and b) CASPT2 excitation energy errors calculated with active spaces chosen by CASCI-EDM-AS, averaged over all dark excitations.

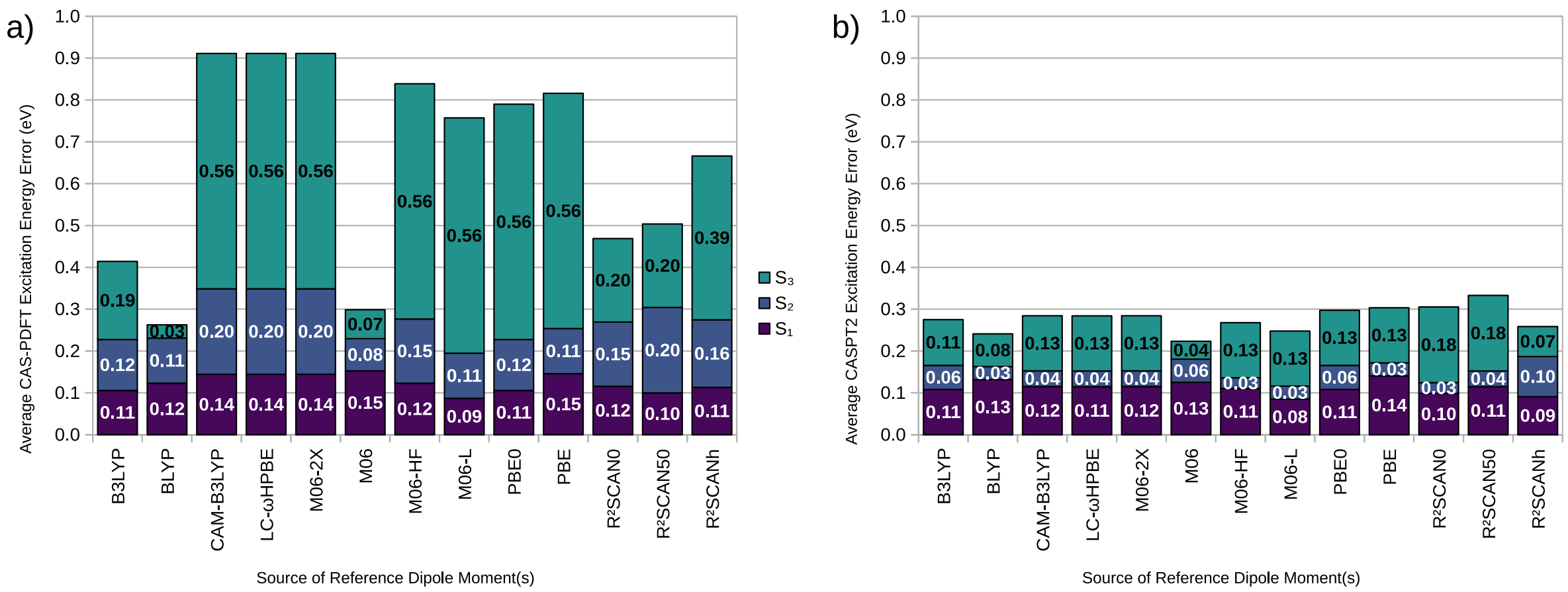

Figure S9: Low-lying a) CAS-PDFT and b) CASPT2 excitation energy errors calculated with active spaces chosen by CASCI-vEDM-AS, averaged over all molecules in the nonpolar dataset.

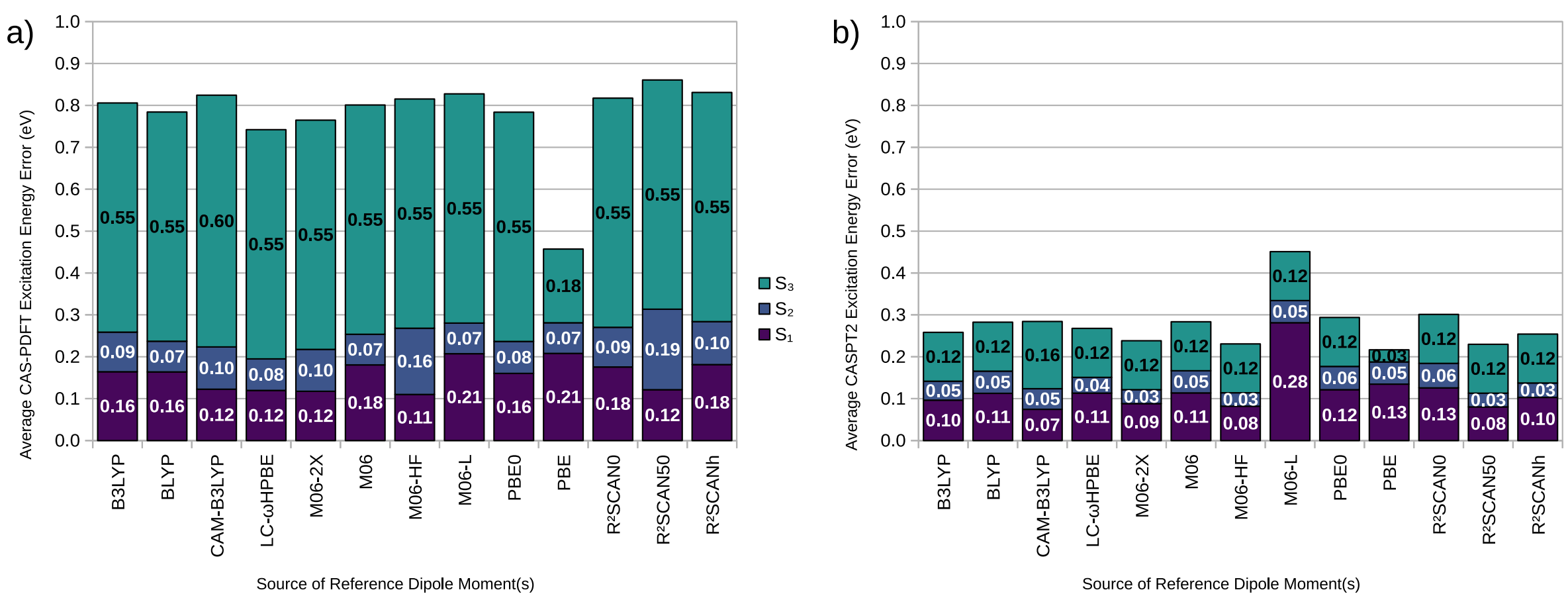

Figure S10: Low-lying a) CAS-PDFT and b) CASPT2 excitation energy errors calculated with active spaces chosen by CASCI-D^{2}DM-AS, averaged over all dark excitations.

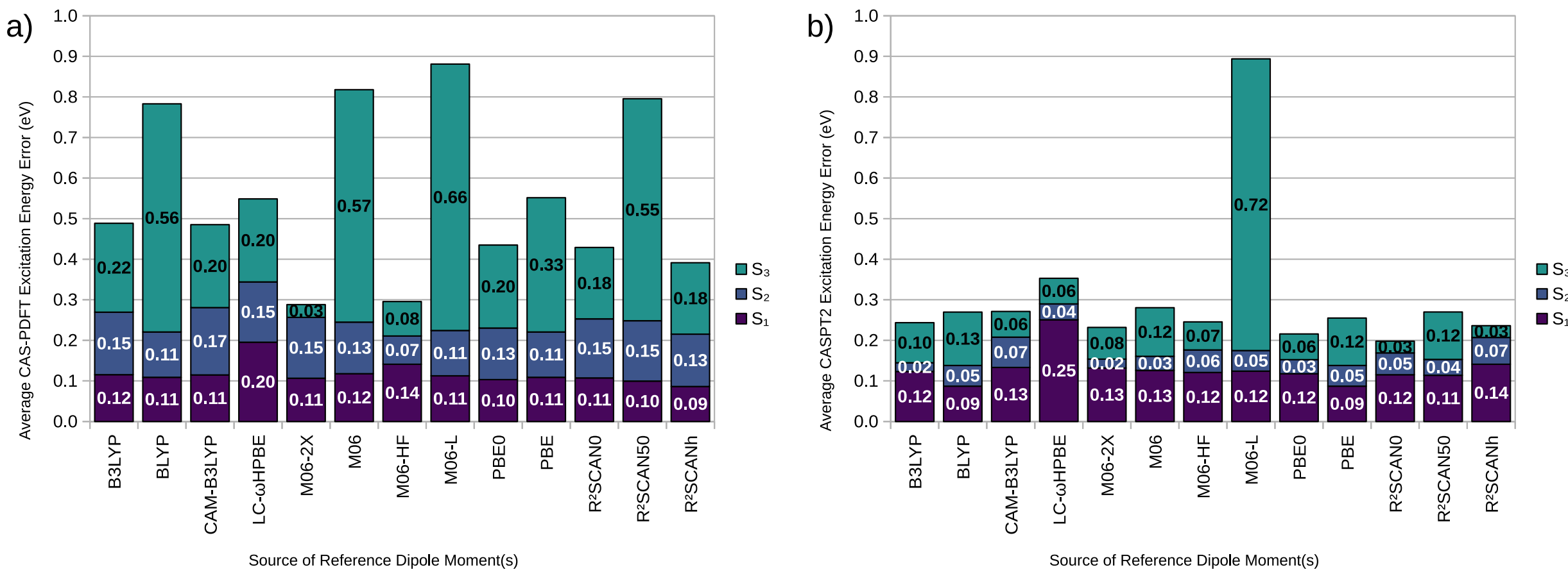

Figure S11: Low-lying a) CAS-PDFT and b) CASPT2 excitation energy errors calculated with active spaces chosen by CASCI-vD^{2}DM-AS, averaged over all dark excitations.

7 Protocol Performance with Conjugated Polar Molecules

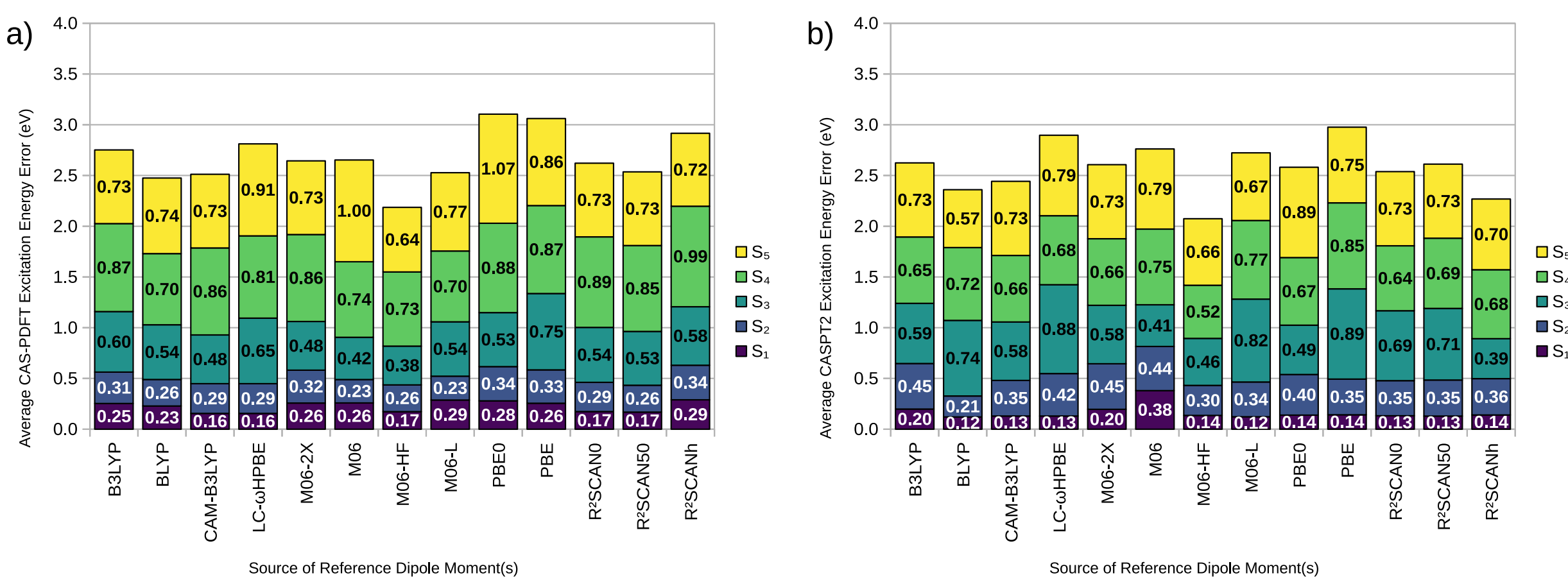

Figure S12: Low-lying a) CAS-PDFT and b) CASPT2 excitation energy errors calculated with active spaces chosen by CASCI-GDM-AS, averaged over all tested conjugated polar molecules.

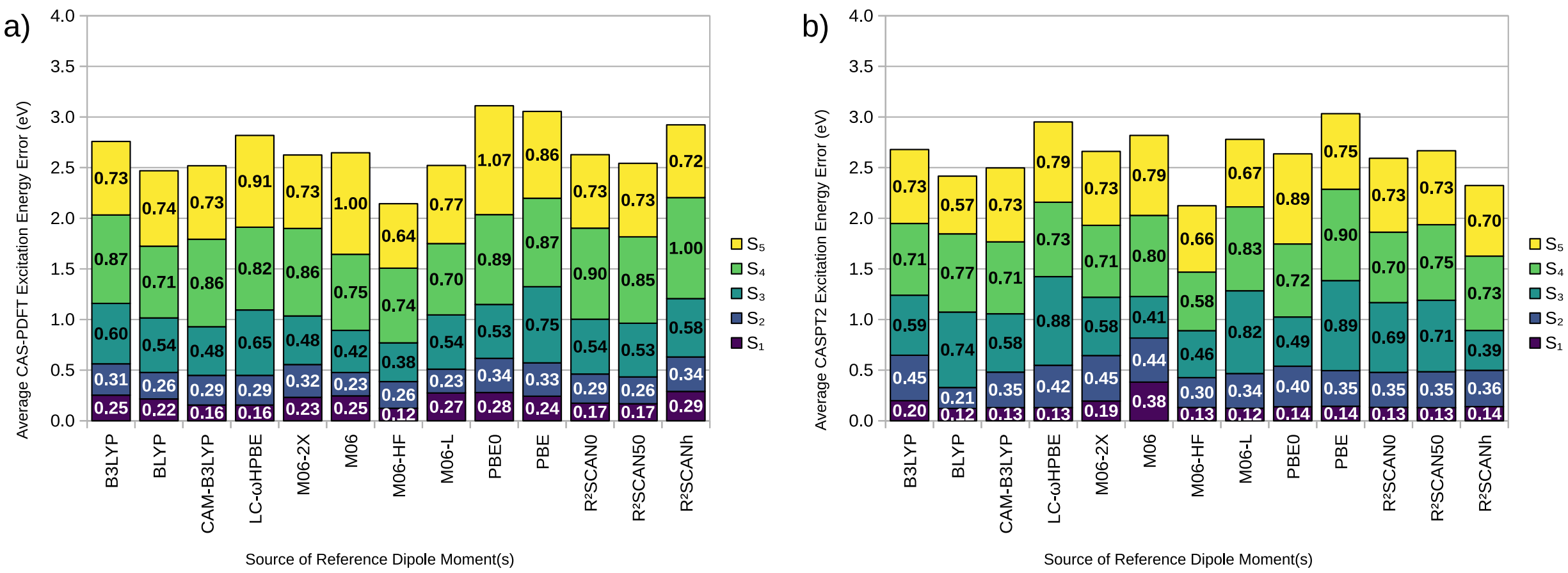

Figure S13: Low-lying a) CAS-PDFT and b) CASPT2 excitation energy errors calculated with active spaces chosen by CASCI-vGDM-AS, averaged over all tested conjugated polar molecules.

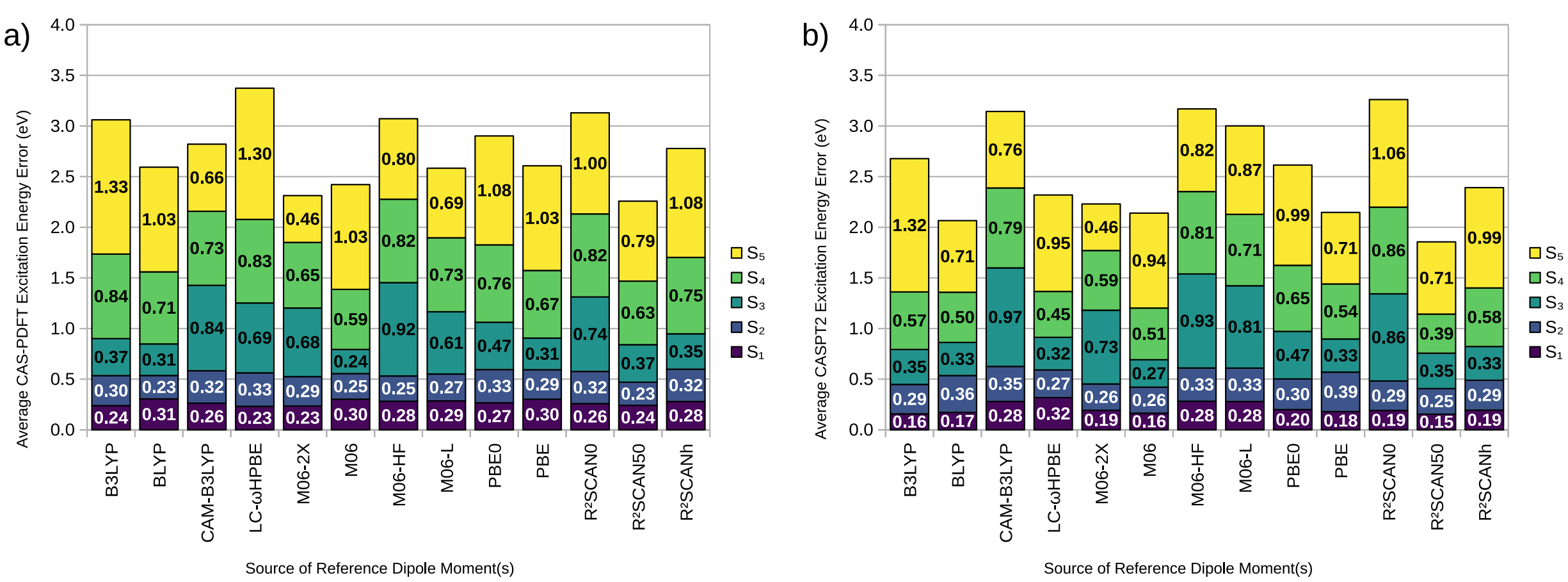

Figure S14: Low-lying a) CAS-PDFT and b) CASPT2 excitation energy errors calculated with active spaces chosen by CASCI-EDM-AS, averaged over all tested conjugated polar molecules.

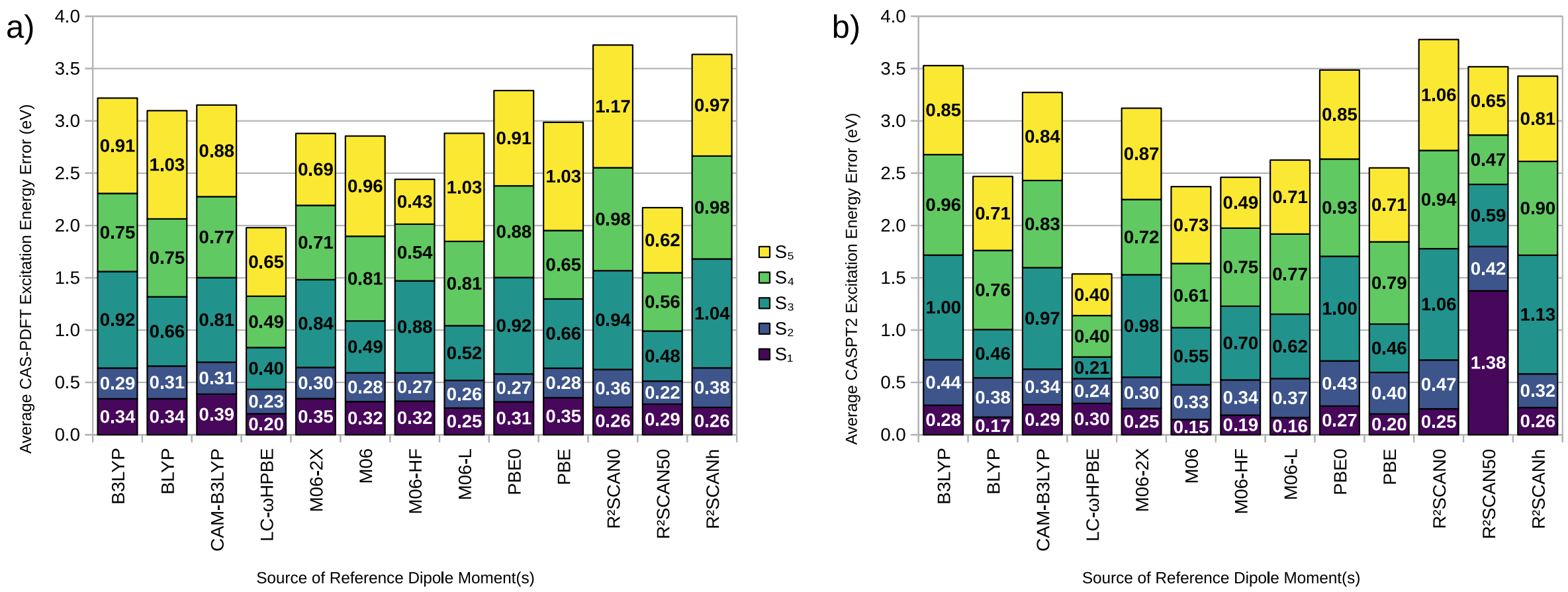

Figure S15: Low-lying a) CAS-PDFT and b) CASPT2 excitation energy errors calculated with active spaces chosen by CASCI-vEDM-AS, averaged over all tested conjugated polar molecules.

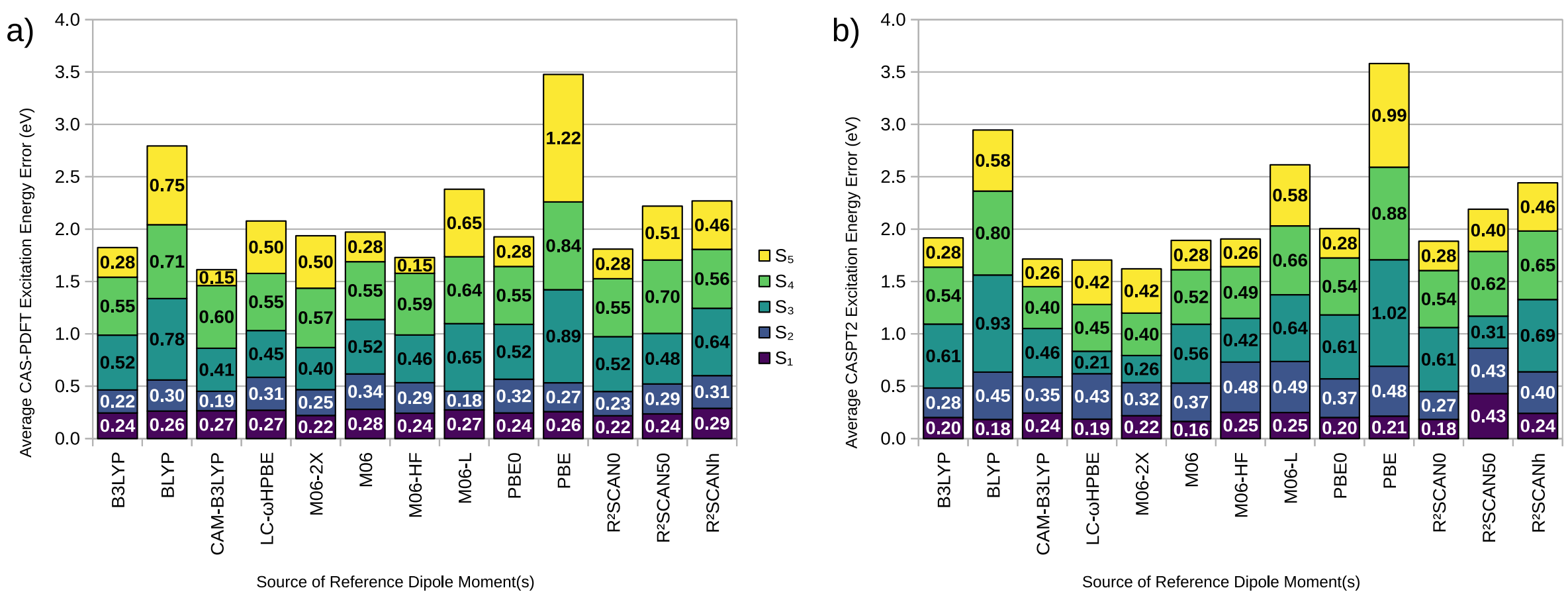

Figure S16: Low-lying a) CAS-PDFT and b) CASPT2 excitation energy errors calculated with active spaces chosen by CASCI-D^{2}DM-AS, averaged over all tested conjugated polar molecules.

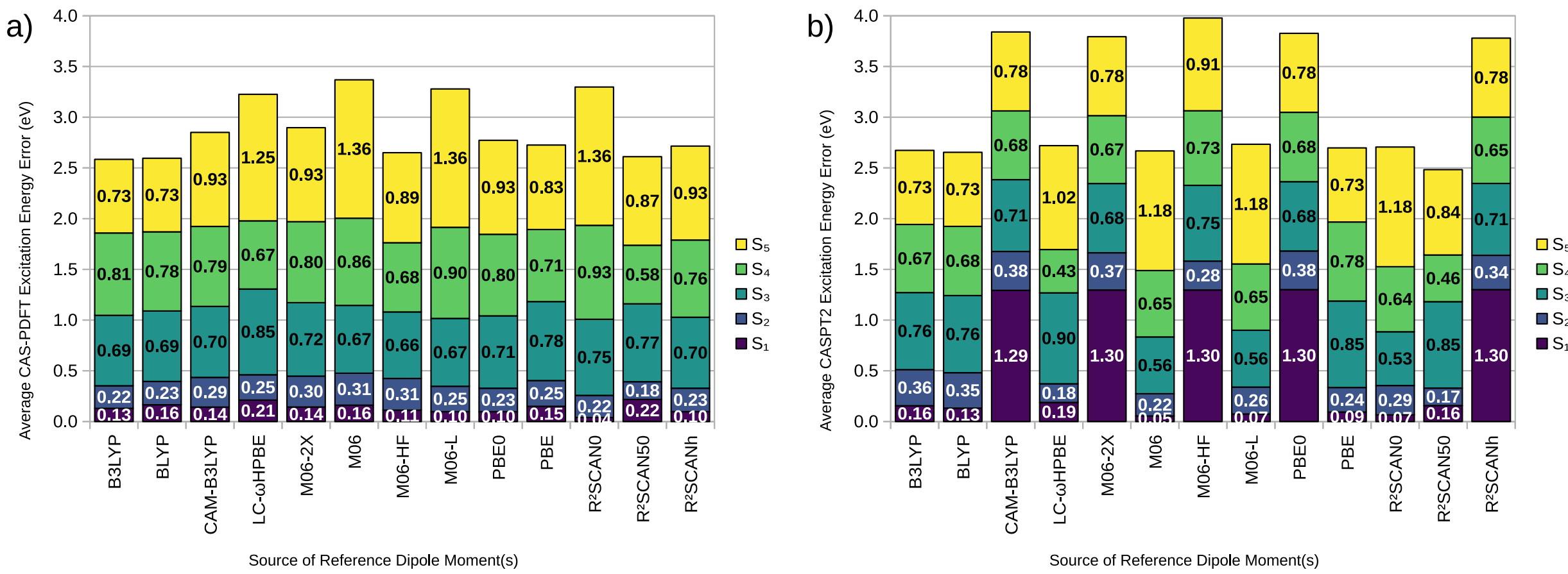

Figure S17: Low-lying a) CAS-PDFT and b) CASPT2 excitation energy errors calculated with active spaces chosen by CASCI-vD^{2}DM-AS, averaged over all tested conjugated polar molecules.

8 Protocol Performance with Nonpolar Molecules

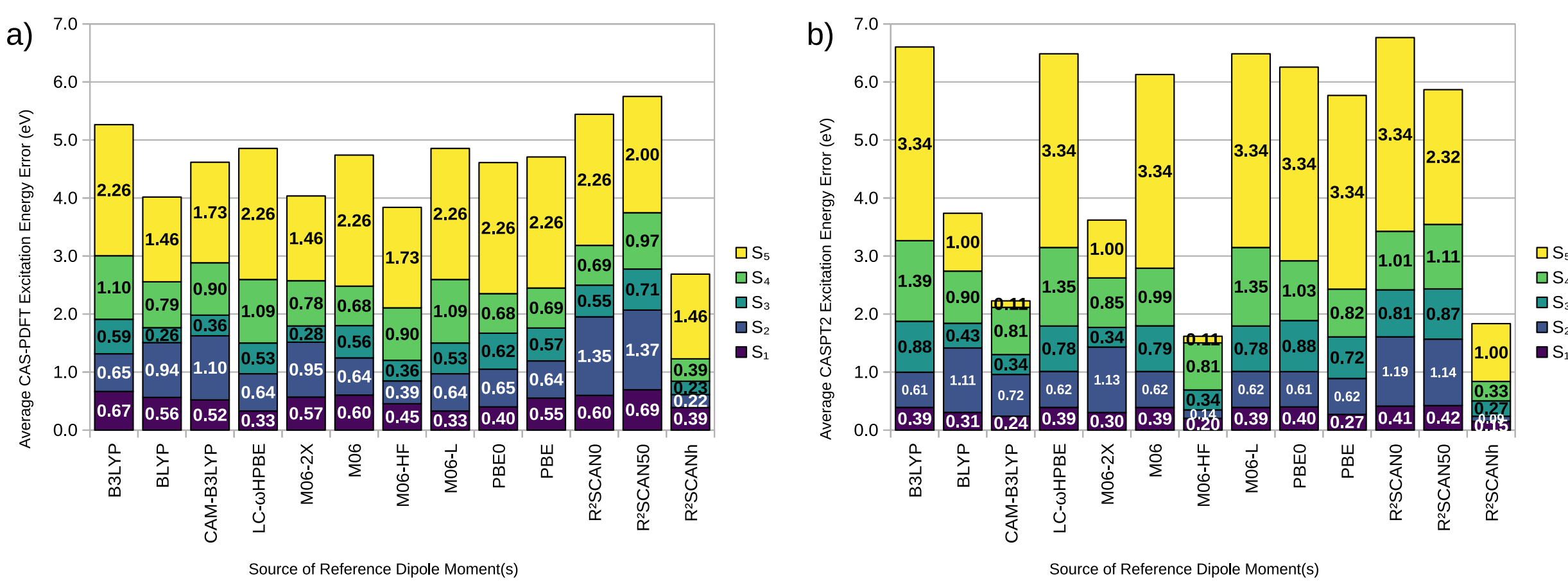

Figure S18: Low-lying a) CAS-PDFT and b) CASPT2 excitation energy errors calculated with active spaces chosen by CASCI-EDM-AS, averaged over all molecules in the nonpolar dataset.

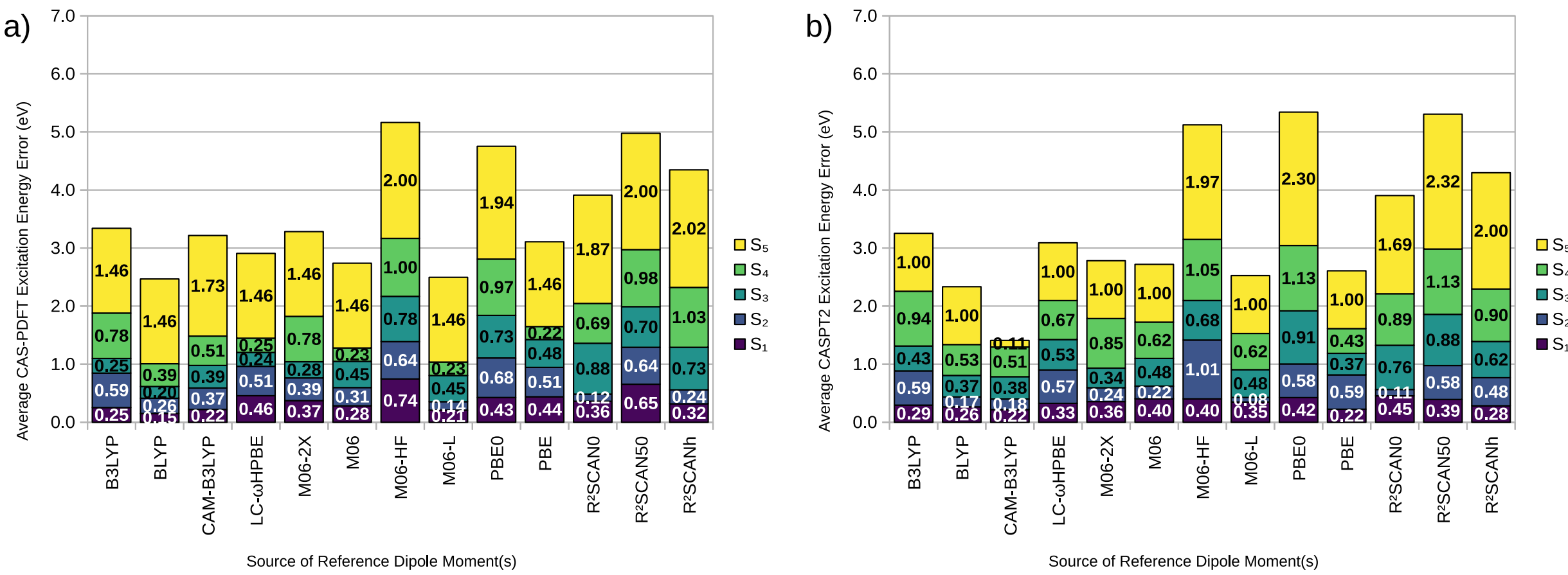

Figure S19: Low-lying a) CAS-PDFT and b) CASPT2 excitation energy errors calculated with active spaces chosen by CASCI-vEDM-AS, averaged over all molecules in the nonpolar dataset.

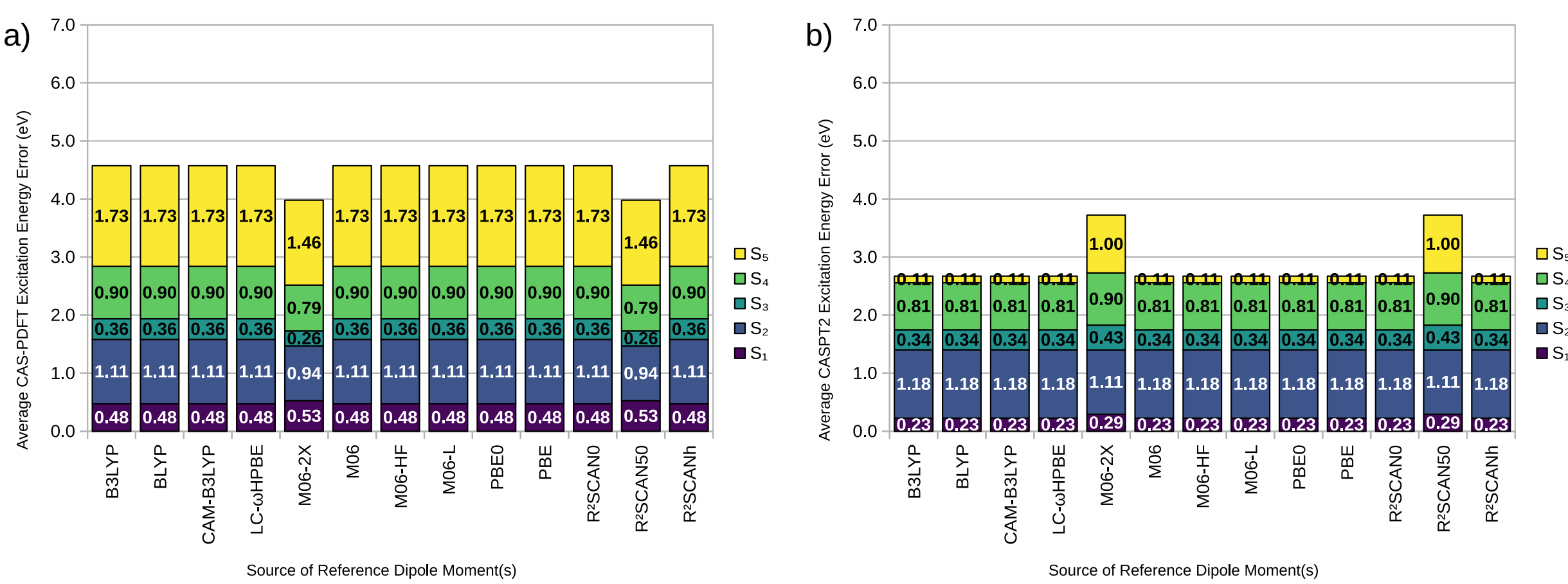

Figure S20: Low-lying a) CAS-PDFT and b) CASPT2 excitation energy errors calculated with active spaces chosen by CASCI-D^2DM-AS, averaged over all molecules in the nonpolar dataset.

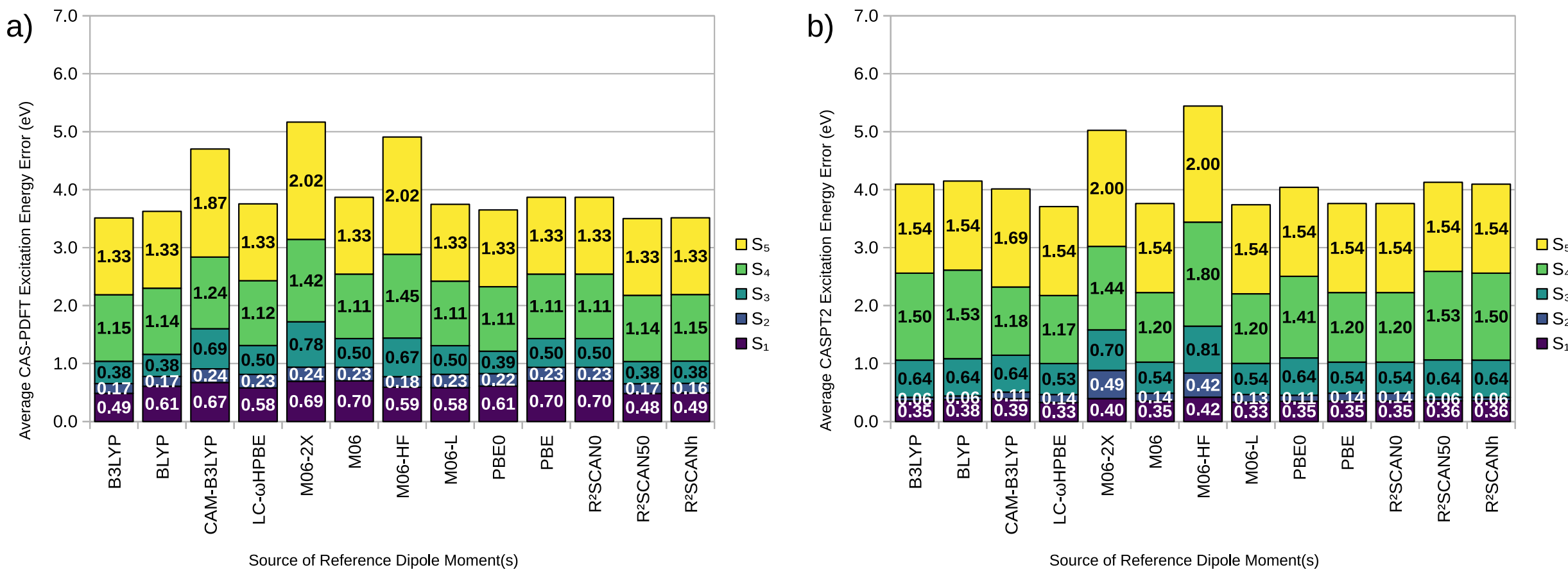

Figure S21: Low-lying a) CAS-PDFT and b) CASPT2 excitation energy errors calculated with active spaces chosen by CASCI-vD^{2}DM-AS, averaged over all molecules in the nonpolar dataset.

9 Protocol Performance with Transition Metal Monoxides

Table S26: CASSCF S_0 energies of transition metal monoxides at various spin states in eV. Energies are relative, with the lowest-energy state set to have an energy of 0. Two active spaces are shown for MnO as they tied for having the lowest dipole moment difference from the reference. Blank: No valid wavefunction for given active space and spin. †: UHF or CASSCF failed to converge.

Complex	Active Space	Relative Energy at Each Multiplicity (eV)							
		1	2	3	4	5	6	7	8
ScO	(7,11)		0		3.88		11.29		†
TiO	(8,14)	0.51		0		5.16		†	
VO	(5,11)		0.70		0		2.70		
CrO	(6,14)	2.17		0.79		0		1.02	
MnO	(7,13)		3.02		0.82		0		1.07
	(7,9)		3.49		1.31		0		1.79

Table S27: CASPT2 S_0 energies of transition metal monoxides at various spin states in eV. Energies are relative, with the lowest-energy state set to have an energy of 0. Two active spaces are shown for MnO as they tied for having the lowest dipole moment difference from the reference. Blank: No valid wavefunction for given active space and spin. †: UHF or CASSCF failed to converge.

Complex	Active Space	Relative Energy at Each Multiplicity (eV)							
		1	2	3	4	5	6	7	8
ScO	(7,11)		0		4.21		11.66		†
TiO	(8,14)	0.43		0		4.64		†	
VO	(5,11)		0.76		0		4.09		
CrO	(6,14)	1.84		0.79		0		1.87	
MnO	(7,13)		2.38		0.55		0		1.05
	(7,9)		4.13		0.99		0		1.77

Table S28: CAS-PDFT S_0 energies of transition metal monoxides at various spin states in eV. Energies are relative, with the lowest-energy state set to have an energy of 0. Two active spaces are shown for MnO as they tied for having the lowest dipole moment difference from the reference. Blank: No valid wavefunction for given active space and spin. †: UHF or CASSCF failed to converge.

Complex	Active Space	Relative Energy at Each Multiplicity (eV)							
		1	2	3	4	5	6	7	8
ScO	(7,11)		0		4.18		11.89		†
TiO	(8,14)	0.60		0		4.67		†	
VO	(5,11)		0.83		0		4.26		
CrO	(6,14)	1.57		0.90		0		1.85	
MnO	(7,13)		1.60		0.45		0		1.25
	(7,9)		3.27		1.18		0		2.02

10 Protocol Performance with Bimolecular Complexes

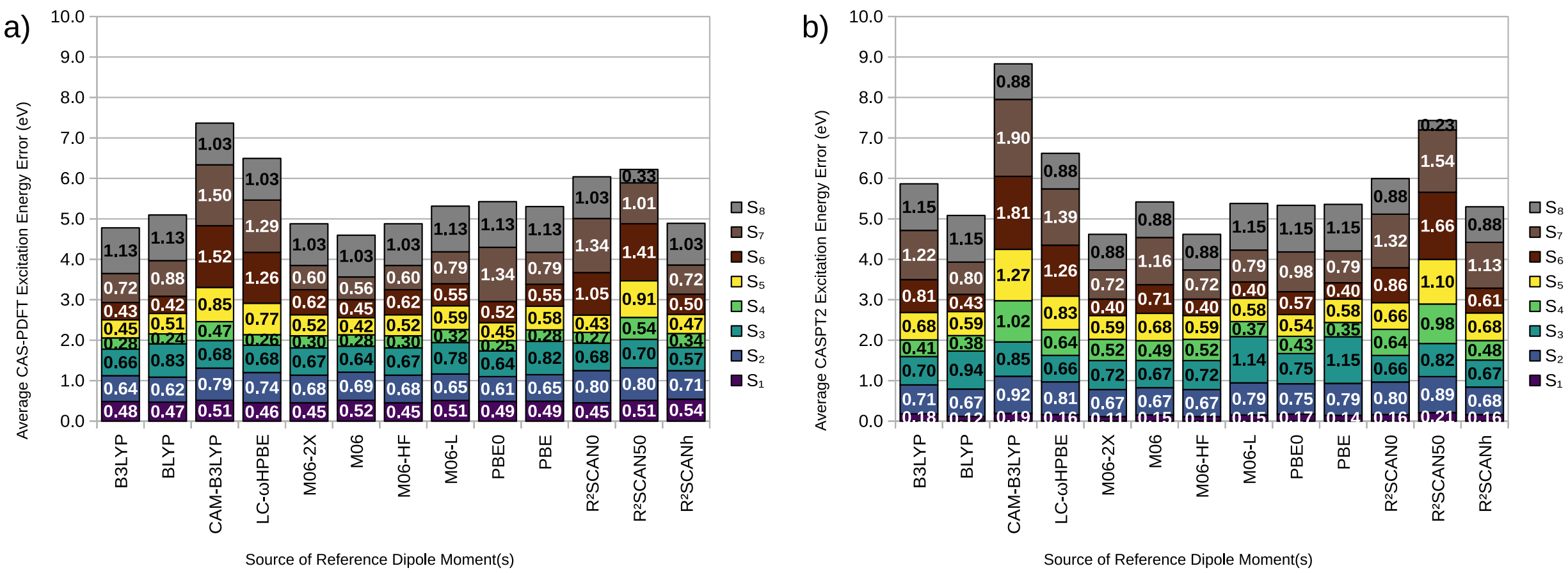

Figure S22: Low-lying a) CAS-PDFT and b) CASPT2 excitation energy errors calculated with active spaces chosen by CASCI-GDM-AS, averaged over all tested bimolecular complexes.

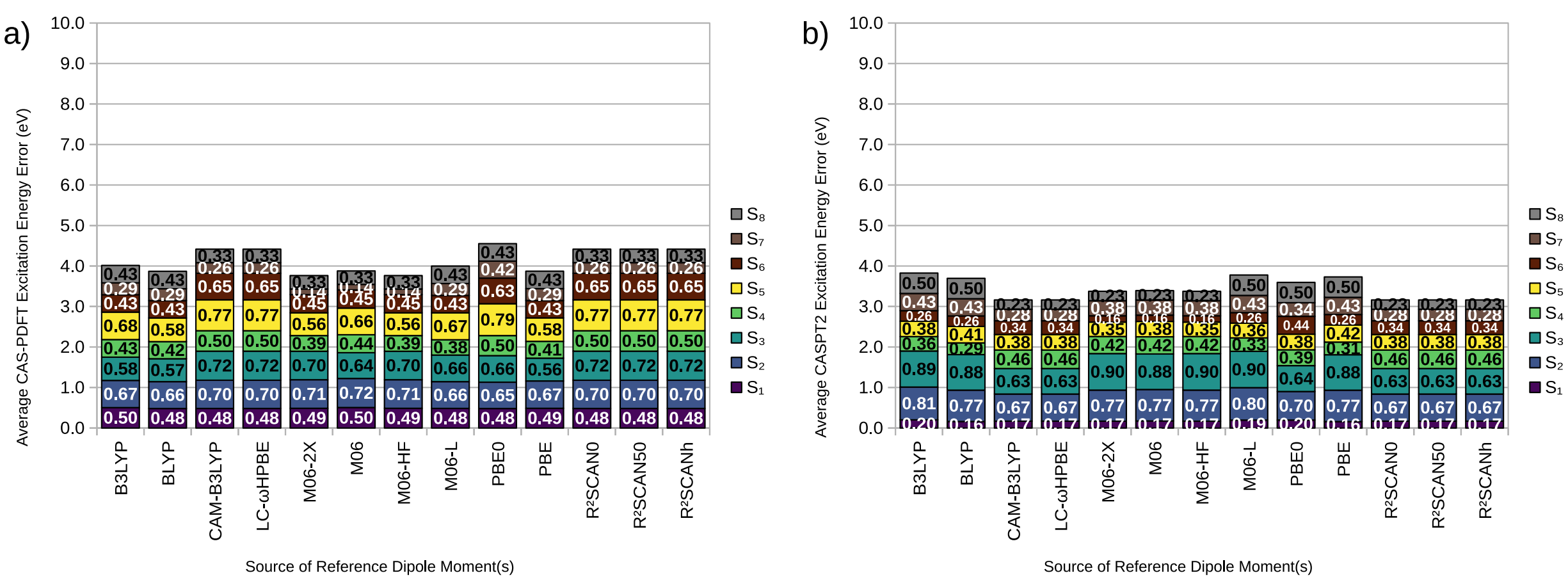

Figure S23: Low-lying a) CAS-PDFT and b) CASPT2 excitation energy errors calculated with active spaces chosen by CASCI-vGDM-AS, averaged over all tested bimolecular complexes.

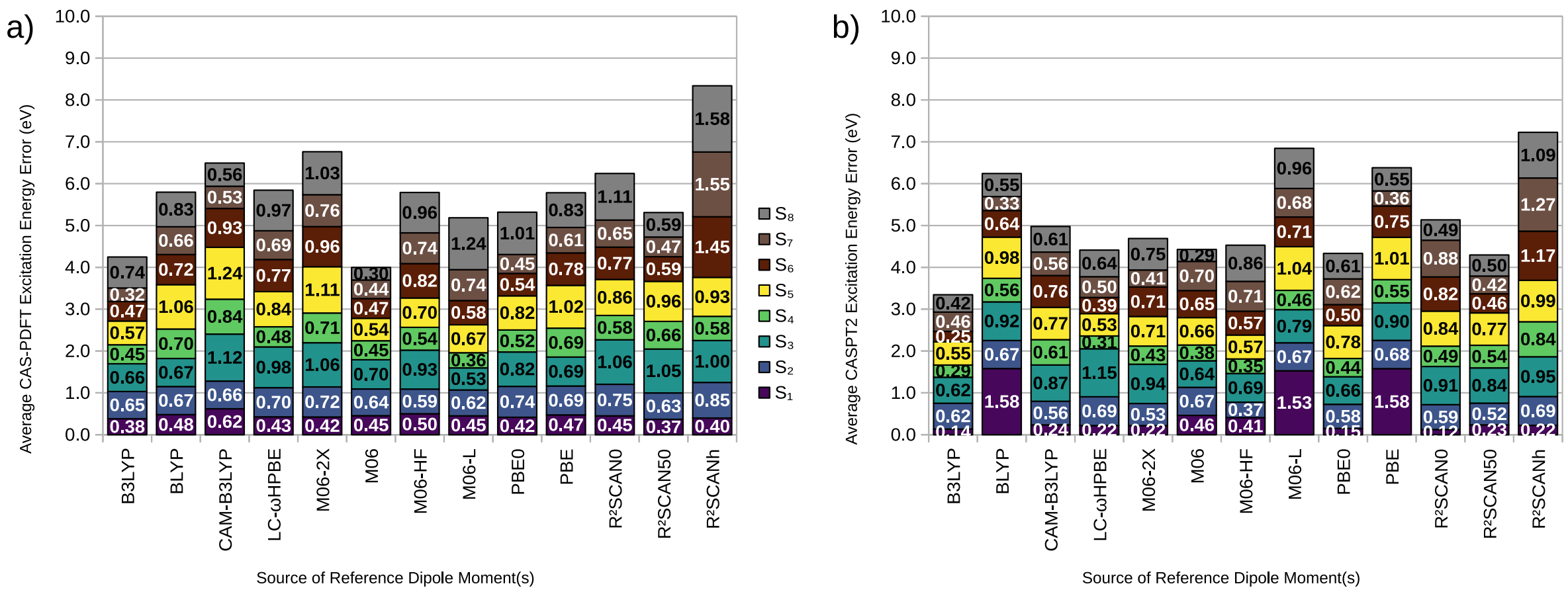

Figure S24: Low-lying a) CAS-PDFT and b) CASPT2 excitation energy errors calculated with active spaces chosen by CASCI-EDM-AS, averaged over all tested bimolecular complexes.

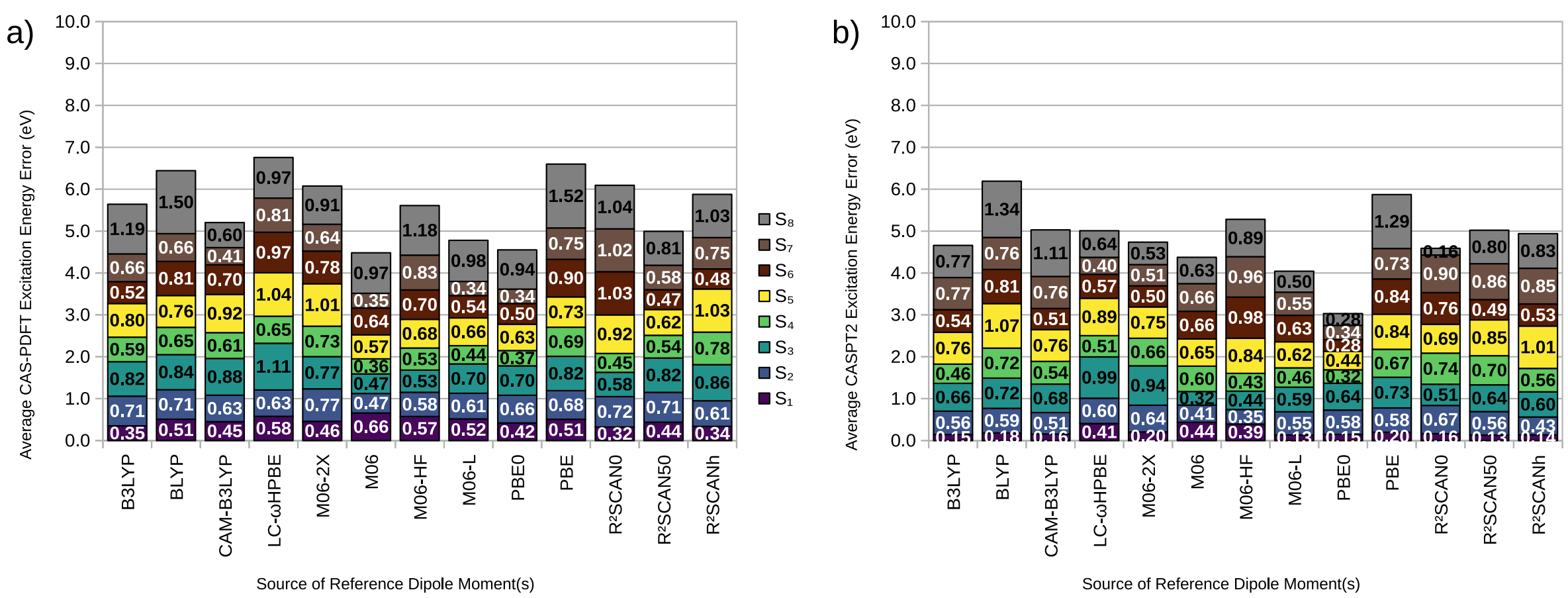

Figure S25: Low-lying a) CAS-PDFT and b) CASPT2 excitation energy errors calculated with active spaces chosen by CASCI-vEDM-AS, averaged over all tested bimolecular complexes.

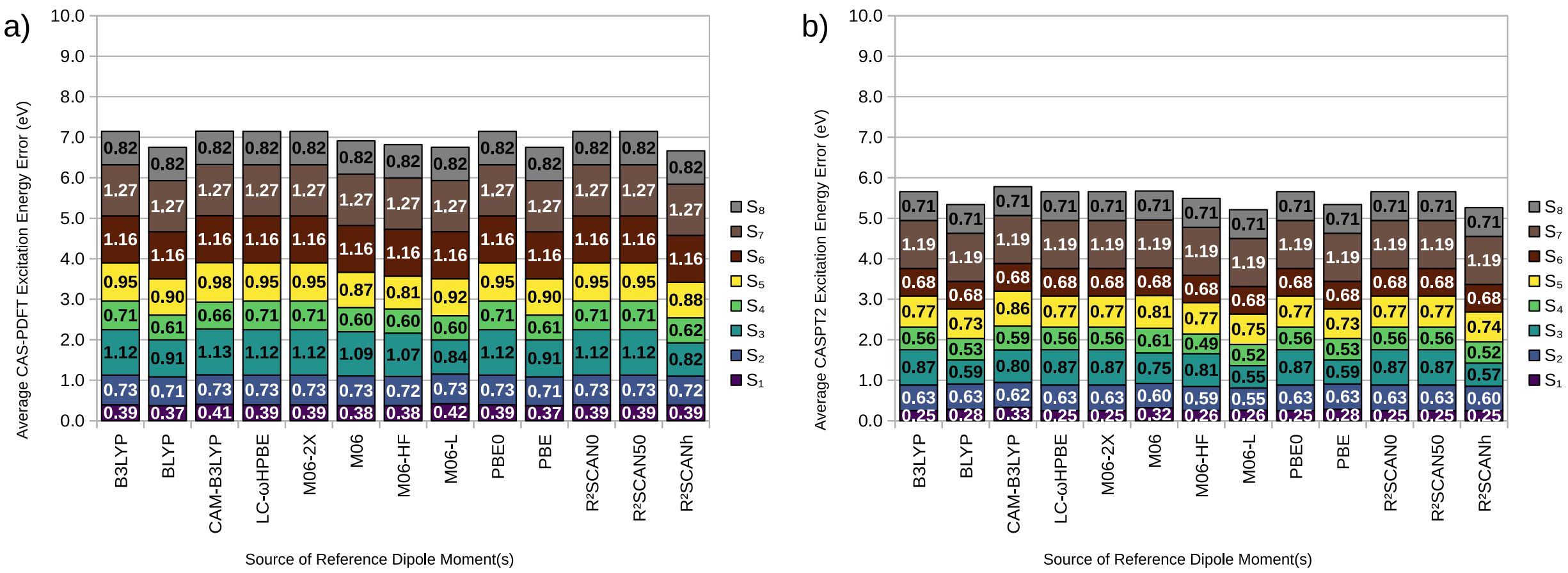

Figure S26: Low-lying a) CAS-PDFT and b) CASPT2 excitation energy errors calculated with active spaces chosen by CASCI-D^{2}DM-AS, averaged over all tested bimolecular complexes.

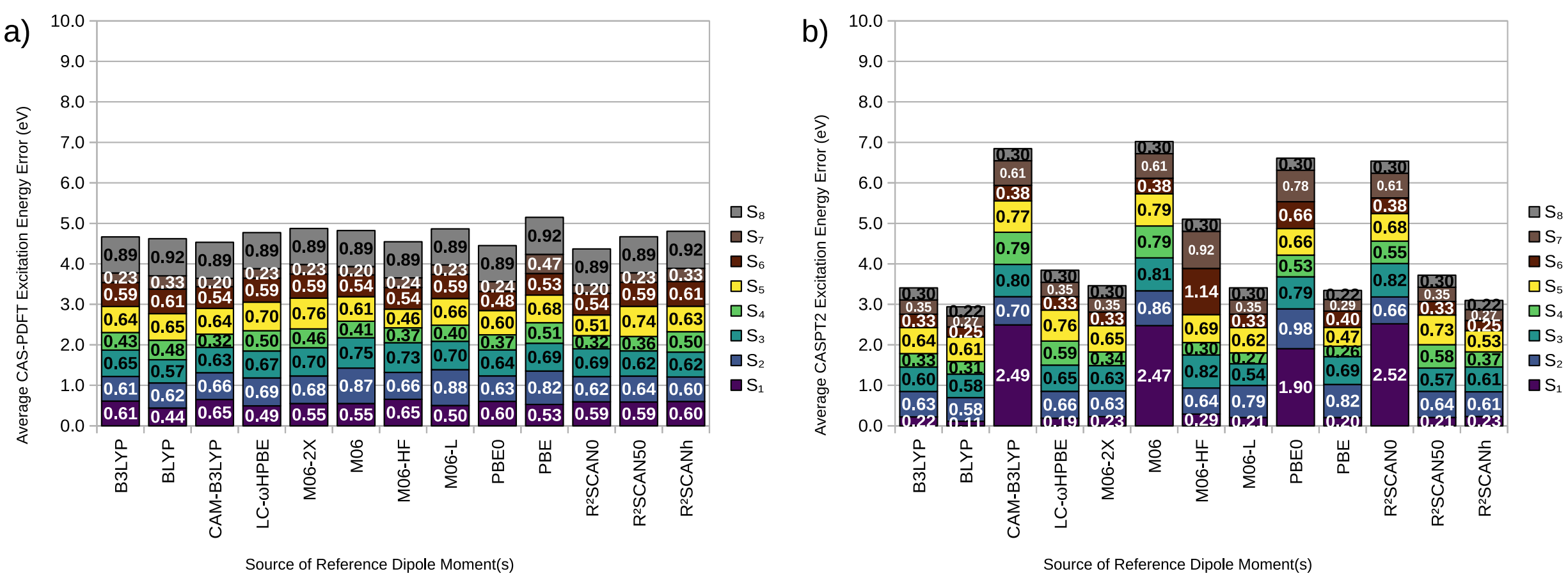

Figure S27: Low-lying a) CAS-PDFT and b) CASPT2 excitation energy errors calculated with active spaces chosen by CASCI-vD^{2}DM-AS, averaged over all tested bimolecular complexes.

11 Ratio of Run Times

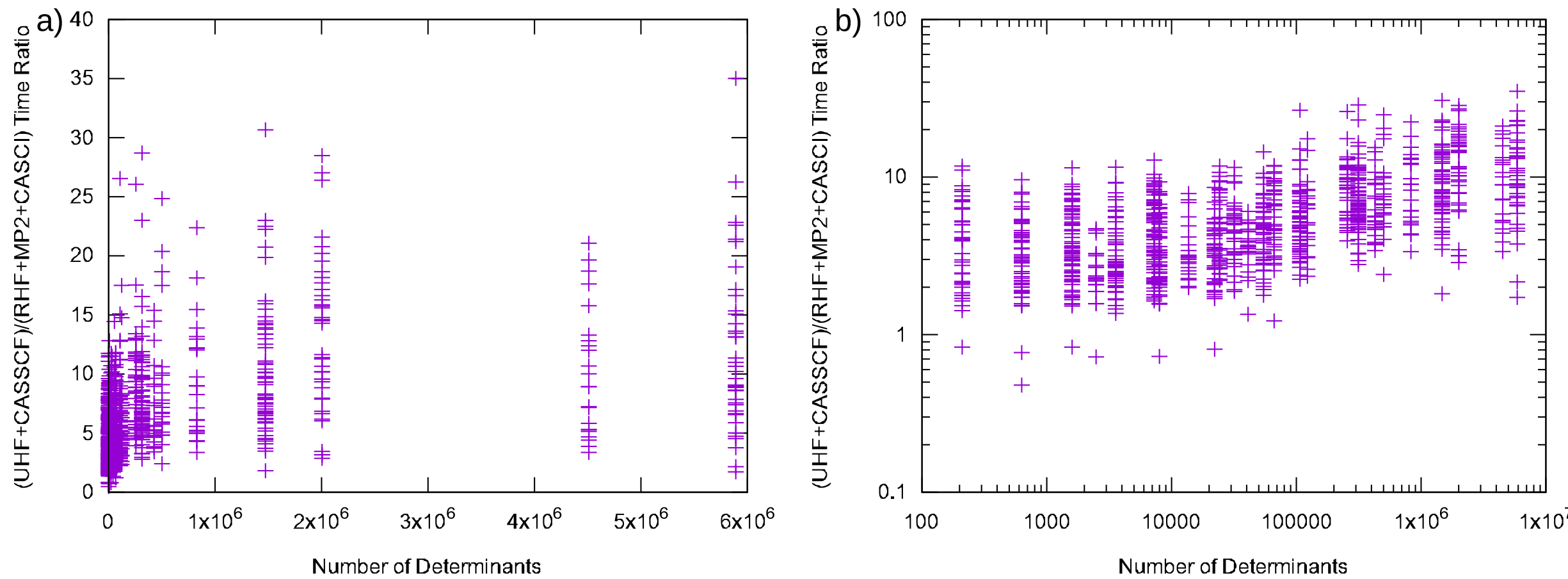

Figure S28: Ratio of combined run times of UHF + CASSCF, and MP2 + CASCI (this work), for all molecules in NAQ, the set of conjugated polar molecules, and the set of nonpolar molecules, and active spaces tested in this work. a) Linear axes; b) log-log axes.

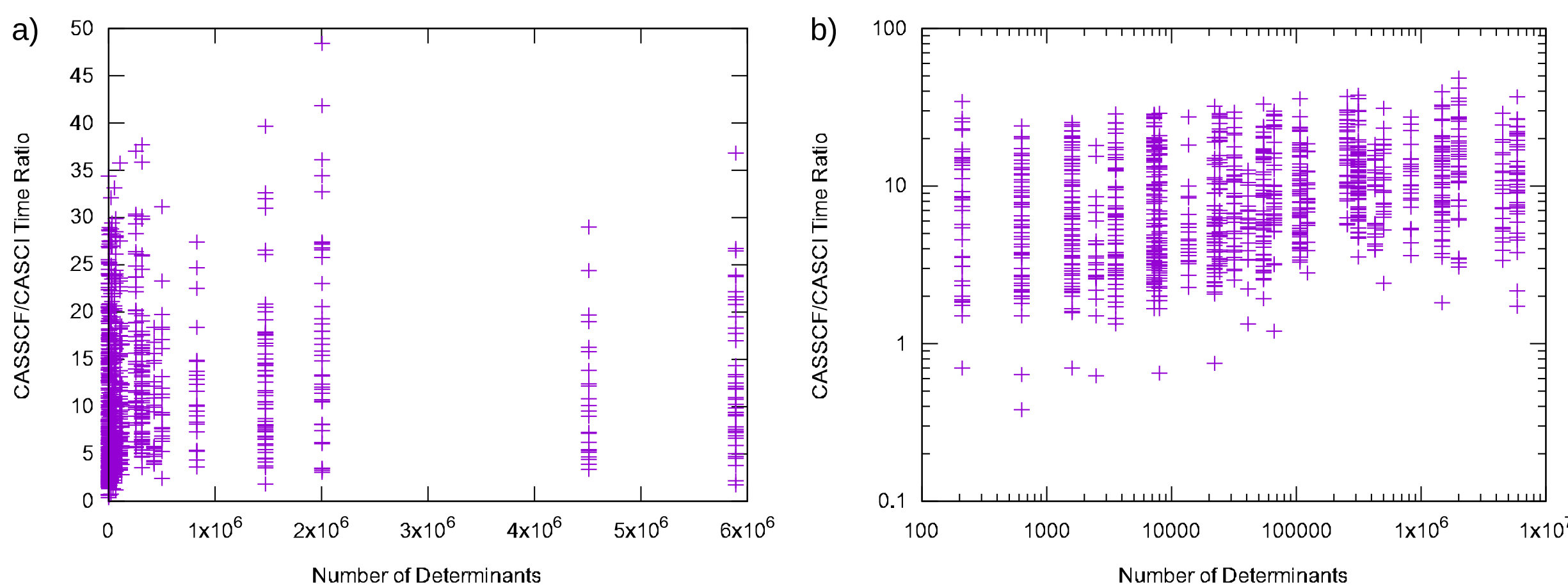

Figure S29: Ratio of run times for CASSCF and CASCI (this work), for all molecules in NAQ, the set of conjugated polar molecules, and the set of nonpolar molecules, and all active spaces tested in this work. a) Linear axes; b) log-log axes.

12 Run Time Data for Other Datasets

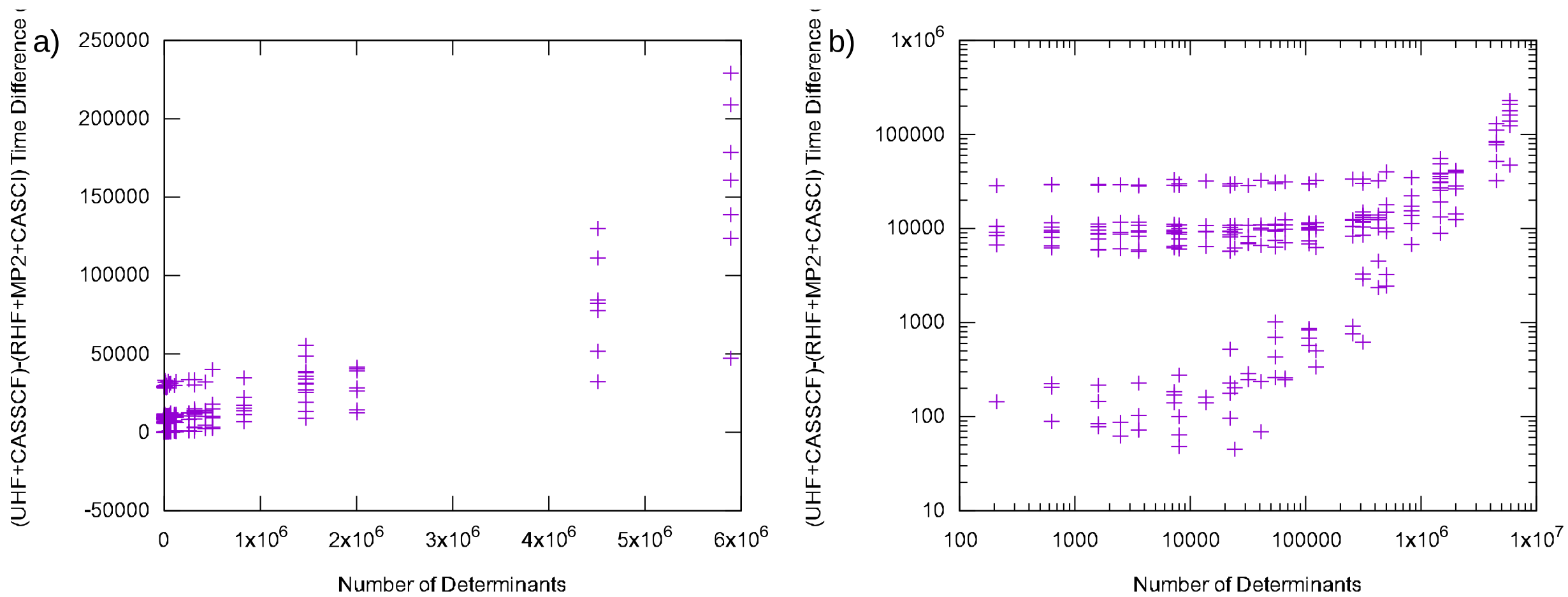

Figure S30: Difference in combined run times of UHF + CASSCF, and MP2 + CASCI (this work), for bimolecular complexes with all active spaces tested in this work. a) Linear axes; b) log-log axes.

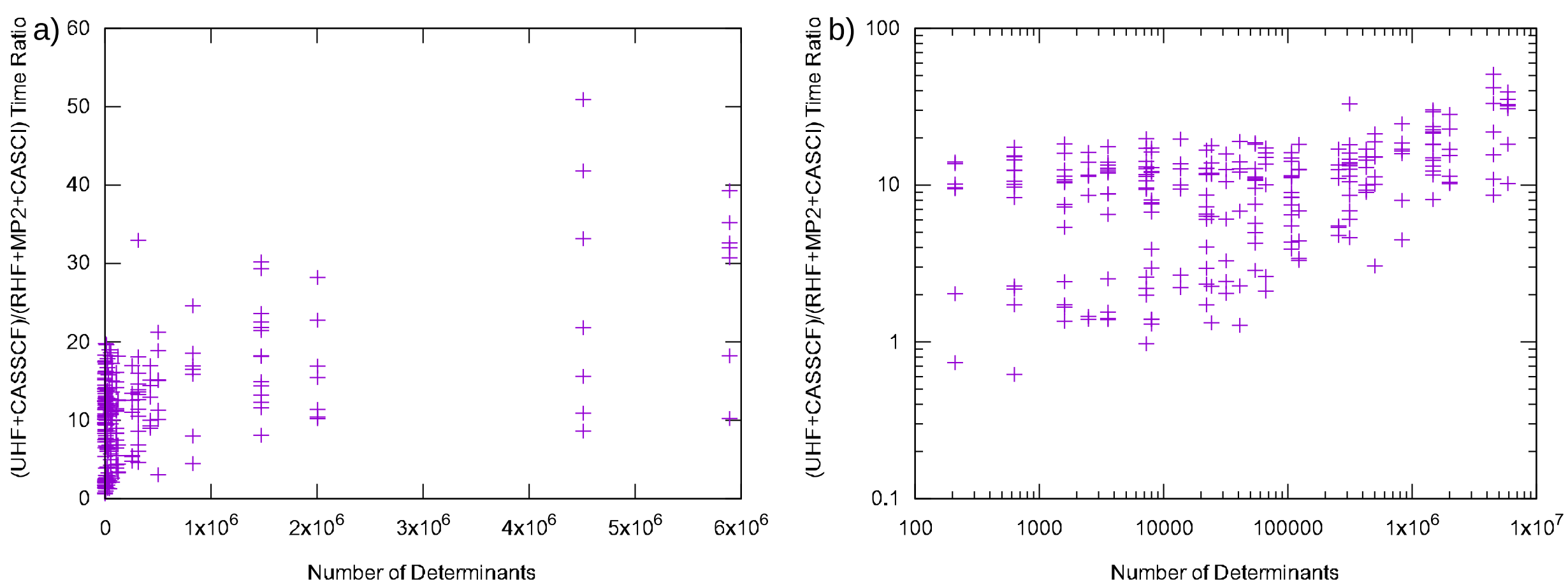

Figure S31: Ratio of combined run times of UHF + CASSCF, and MP2 + CASCI (this work), for bimolecular complexes with all active spaces tested in this work. a) Linear axes; b) log-log axes.

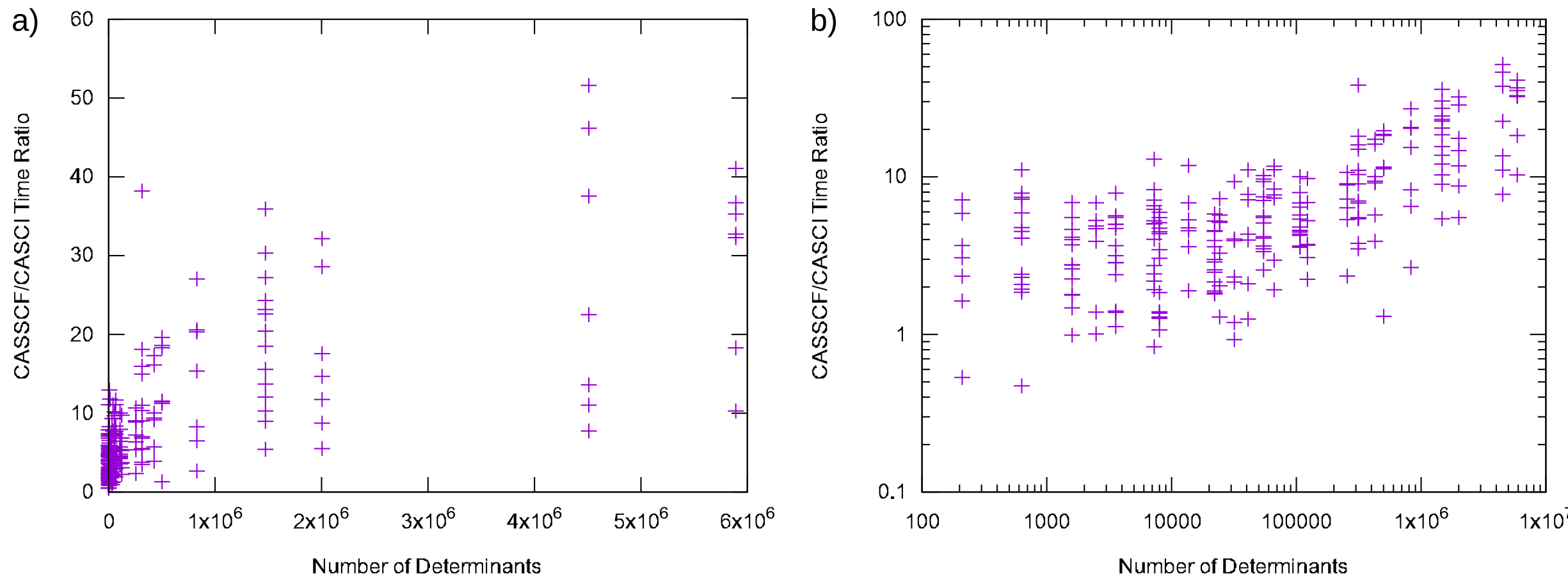

Figure S32: Ratio of run times for CASSCF and CASCI (this work), for bimolecular complexes with all active spaces tested in this work. a) Linear axes; b) log-log axes.

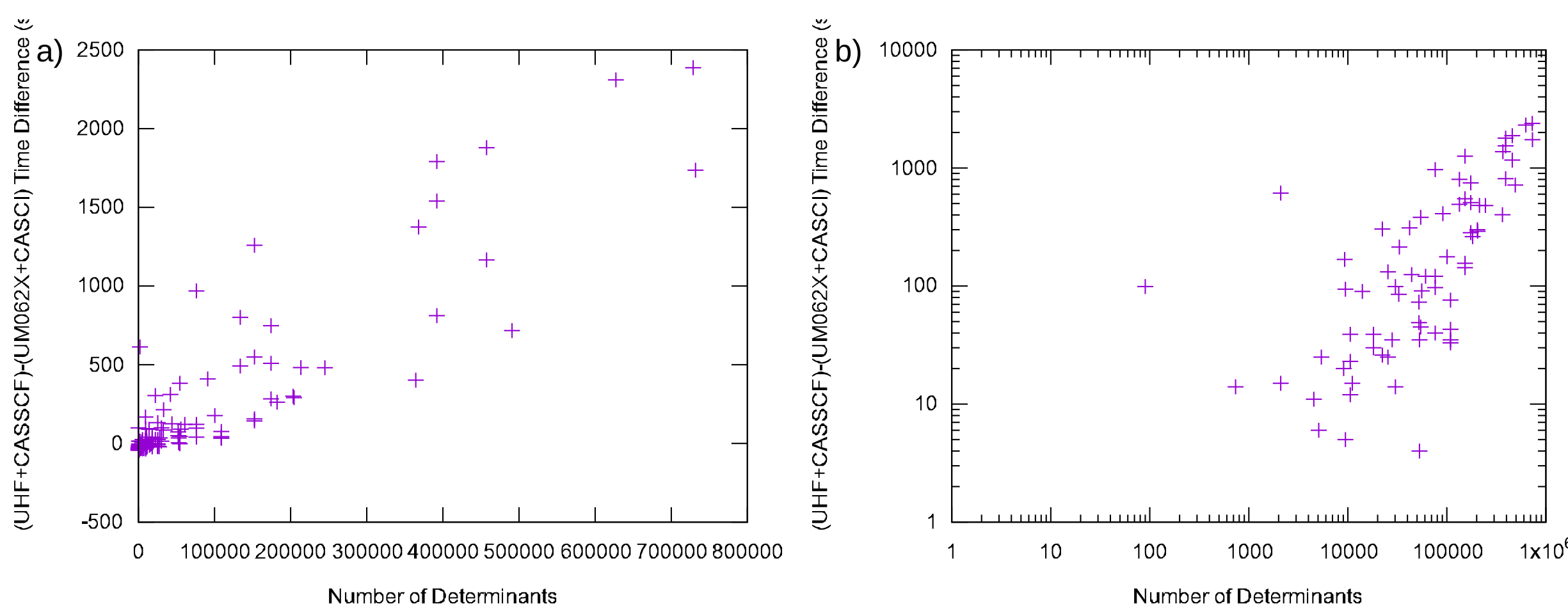

Figure S33: Difference in combined run times of UHF + CASSCF, and UM06-2X + CASCI (this work), for transition metal monoxides with all active spaces tested in this work. a) Linear axes; b) log-log axes.

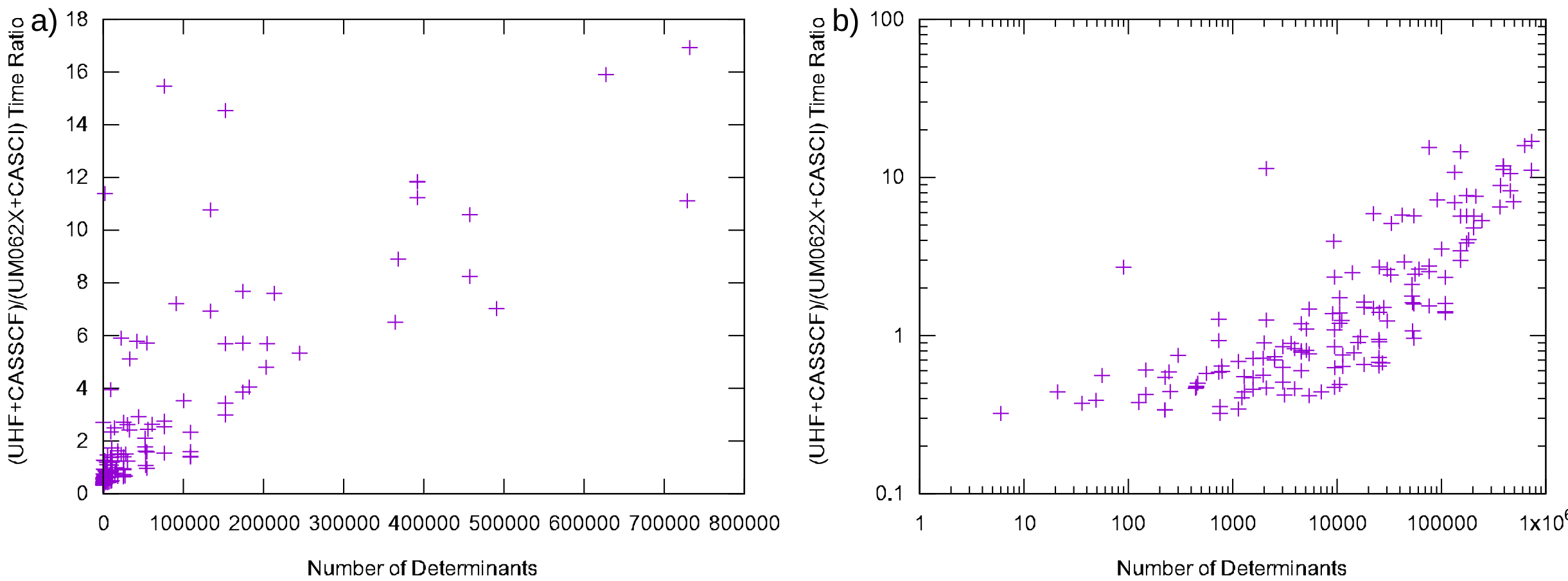

Figure S34: Ratio of combined run times of UHF + CASSCF, and UM06-2X + CASCI (this work), for transition metal monoxides with all active spaces tested in this work. a) Linear axes; b) log-log axes.

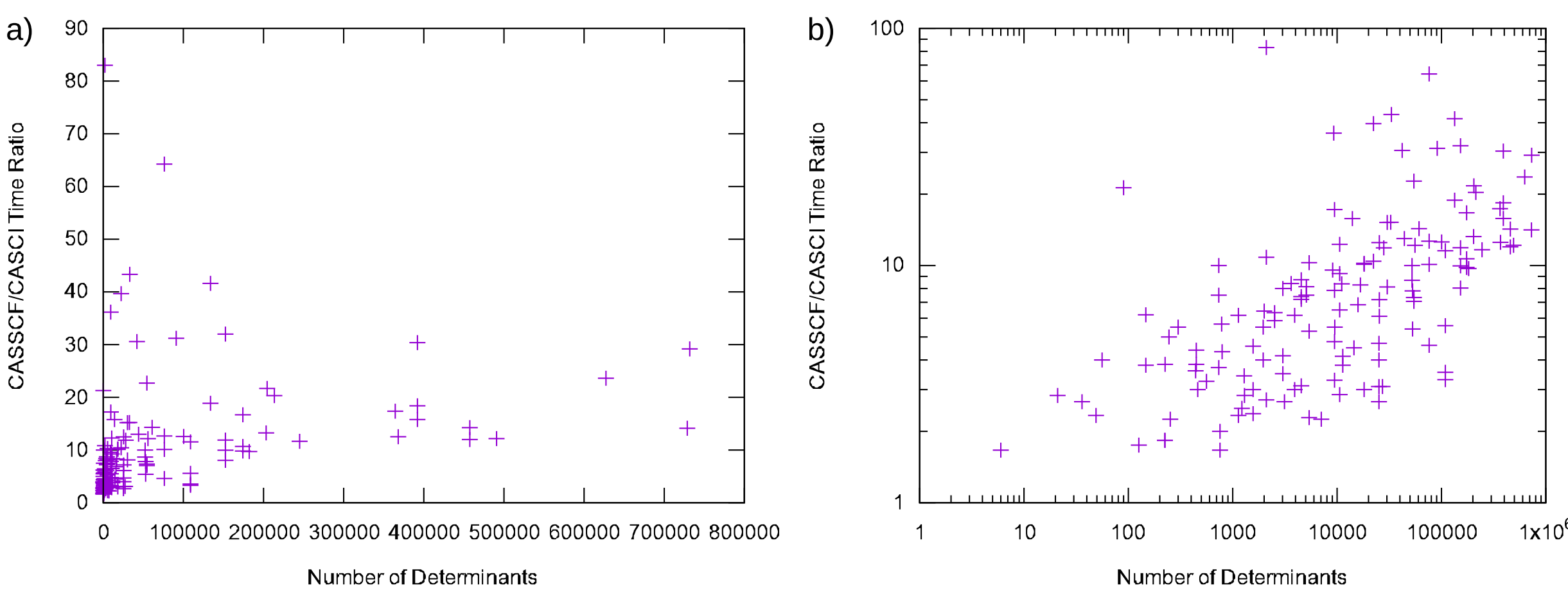

Figure S35: Ratio of run times for CASSCF and CASCI (this work), for for transition metal monoxides with all active spaces tested in this work. a) Linear axes; b) log-log axes.

13 Potential Energy Curve of CO Calculated with Selected Active Spaces

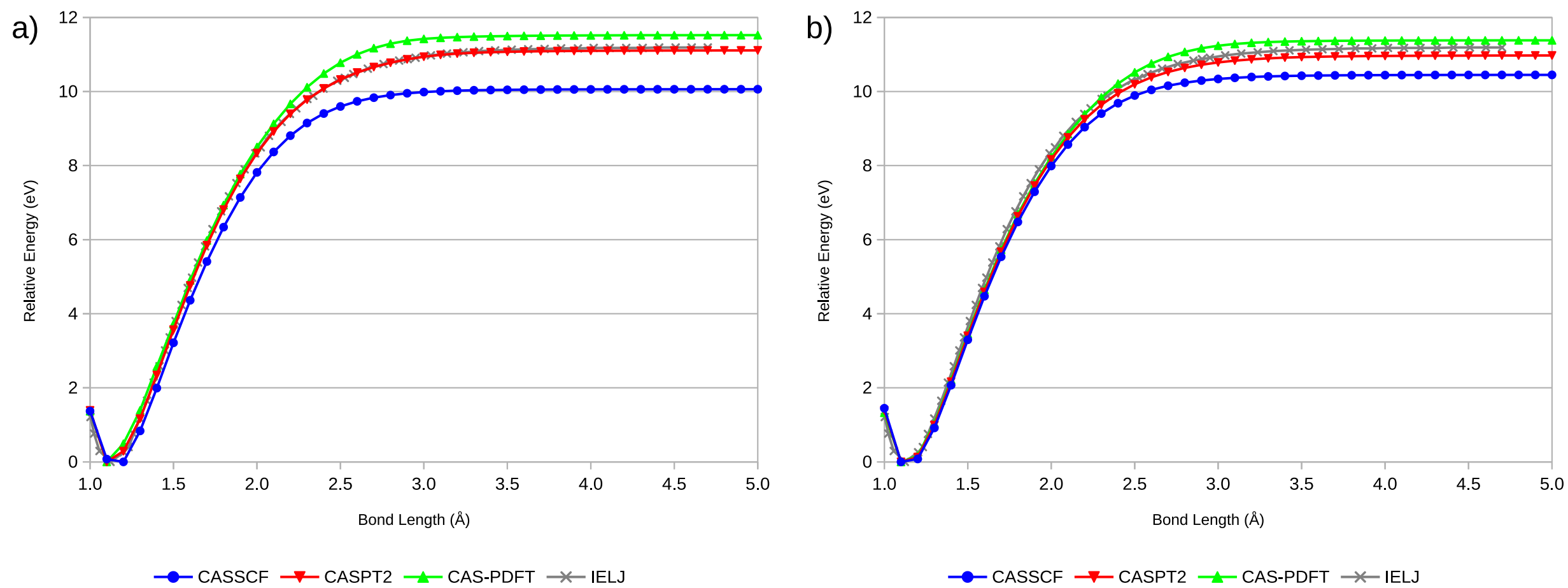

Figure S36: Potential energy curve of carbon monoxide bond dissociation calculated with the a) (6,10) and b) (8,10) active spaces, at the CASSCF, CASPT2 and CAS-PDFT levels. For comparison, the Improved Extended-Lennard Jones (IELJ) potential energy curve, which was digitized from the work of Araújo et al.[19] with WebPlotDigitizer,[20] is also included.

References

(1) Neese, F. The ORCA program system. *WIREs Comput. Mol. Sci.* **2012**, *2*, 73–78.

(2) Neese, F. Software update: the ORCA program system, version 4.0. *WIREs Comput. Mol. Sci.* **2018**, *8*, e1327.

(3) Neese, F.; Wennmohs, F.; Becker, U.; Riplinger, C. The ORCA quantum chemistry program package. *J. Chem. Phys.* **2020**, *152*, 224108.

(4) Roos, B. O.; Lindh, R.; Malmqvist, P.-Å.; Veryazov, V.; Widmark, P.-O. Main Group Atoms and Dimers Studied with a New Relativistic ANO Basis Set. *J. Phys. Chem. A* **2004**, *108*, 2851–2858.

(5) Véril, M.; Scemama, A.; Caffarel, M.; Lipparini, F.; Boggio-Pasqua, M.; Jacquemin, D.; Loos, P.-F. QUESTDB: A database of highly accurate excitation energies for the electronic structure community. *WIRES Comput. Mol. Sci.* **2021**, *11*, e1517.

(6) Hanwell, M. D.; Curtis, D. E.; Lonie, D. C.; Vandermeersch, T.; Zurek, E.; Hutchison, G. R. Avogadro: an advanced semantic chemical editor, visualization, and analysis platform. *J. Cheminform.* **2012**, *4*, 17.

(7) Kozma, B.; Tajti, A.; Demoulin, B.; Izsák, R.; Nooijen, M.; Szalay, P. G. A New Benchmark Set for Excitation Energy of Charge Transfer States: Systematic Investigation of Coupled Cluster Type Methods. *J. Chem. Theory Comput.* **2020**, *16*, 4213–4225.

(8) Vosko, S. H.; Wilk, L.; Nusair, M. Accurate spin-dependent electron liquid correlation energies for local spin density calculations: a critical analysis. *Can. J. Phys.* **1980**, *58*, 1200–1211.

(9) Bartlett, R. J.; Purvis, G. D. Many-body perturbation theory, coupled-pair many-electron theory, and the importance of quadruple excitations for the correlation problem. *Int. J. Quantum Chem.* **1978**, *14*, 561–581.

(10) On the Use of the Cluster Expansion and the Technique of Diagrams in Calculations of Correlation Effects in Atoms and Molecules - Čížek - 1969 - Advances in Chemical Physics - Wiley Online Library. `https://onlinelibrary.wiley.com/doi/10.1002/9780470143599.ch2`.

(11) Purvis, G. D.; Bartlett, R. J. A full coupled-cluster singles and doubles model: The inclusion of disconnected triples. *J. Chem. Phys.* **1982**, *76*, 1910–1918.

(12) Scuseria, G. E.; Janssen, C. L.; Schaefer, H. F. An efficient reformulation of the closed-shell coupled cluster single and double excitation (CCSD) equations. *J. Chem. Phys.* **1988**, *89*, 7382–7387.

(13) Scuseria, G. E.; Schaefer, H. F. Is coupled cluster singles and doubles (CCSD) more computationally intensive than quadratic configuration interaction (QCISD)? *J. Chem. Phys.* **1989**, *90*, 3700–3703.

(14) Pople, J. A.; Head-Gordon, M.; Raghavachari, K. Quadratic configuration interaction. A general technique for determining electron correlation energies. *J. Chem. Phys.* **1987**, *87*, 5968–5975.

(15) Dunning, T. H., Jr. Gaussian basis sets for use in correlated molecular calculations. I. The atoms boron through neon and hydrogen. *J. Chem. Phys.* **1989**, *90*, 1007–1023.

(16) Kendall, R. A.; Dunning, T. H., Jr.; Harrison, R. J. Electron affinities of the first-row atoms revisited. Systematic basis sets and wave functions. *J. Chem. Phys.* **1992**, *96*, 6796–6806.

(17) Woon, D. E.; Dunning, T. H., Jr. Gaussian basis sets for use in correlated molecular calculations. III. The atoms aluminum through argon. *J. Chem. Phys.* **1993**, *98*, 1358–1371.

(18) Loos, P.-F.; Comin, M.; Blase, X.; Jacquemin, D. Reference Energies for Intramolecular Charge-Transfer Excitations. *J. Chem. Theory Comput.* **2021**, *17*, 3666–3686.

(19) Araújo, J. P.; Lugão, I. G.; Silva, R. P.; Martins, M. P.; Okon, I. B.; Onate, C. A. A comparative study of analytical representations of potential energy curves for CO in its ground electronic state. *Int. J. Quantum Chem.* **2024**, *124*, e27429.

(20) Rohatgi, A. WebPlotDigitizer. `https://automeris.io`.
